# Increased Vascular Permeability in the Bone Marrow Microenvironment Contributes to Disease Progression and Drug Response in Acute Myeloid Leukemia

**DOI:** 10.1016/j.ccell.2017.08.001

**Published:** 2017-09-11

**Authors:** Diana Passaro, Alessandro Di Tullio, Ander Abarrategi, Kevin Rouault-Pierre, Katie Foster, Linda Ariza-McNaughton, Beatriz Montaner, Probir Chakravarty, Leena Bhaw, Giovanni Diana, François Lassailly, John Gribben, Dominique Bonnet

**Affiliations:** 1 Haematopoietic Stem Cell Laboratory, The Francis Crick Institute, 1 Midland Road, London NW1 1AT, UK; 2 Bioinformatic Core Unit, The Francis Crick Institute, 1 Midland Road, London NW1 1AT, UK; 3 Advanced Sequencing Unit, The Francis Crick Institute, 1 Midland Road, London NW1 1AT, UK; 4 MRC Centre for Developmental Neurobiology, King's College London, London, UK; 5 Department of Haemato-Oncology, Barts Cancer Institute, Queen Mary University of London, London EC1M 6BQ, UK

**Keywords:** acute myeloid leukemia, hematopoietic stem cells, endothelial cells, vascular permeability, nitric oxide, hypoxia, microenvironment, intravital 2P microscopy, NOS inhibitors, chemotherapy

## Abstract

The biological and clinical behaviors of hematological malignancies can be influenced by the active crosstalk with an altered bone marrow (BM) microenvironment. In the present study, we provide a detailed picture of the BM vasculature in acute myeloid leukemia using intravital two-photon microscopy. We found several abnormalities in the vascular architecture and function in patient-derived xenografts (PDX), such as vascular leakiness and increased hypoxia. Transcriptomic analysis in endothelial cells identified nitric oxide (NO) as major mediator of this phenotype in PDX and in patient-derived biopsies. Moreover, induction chemotherapy failing to restore normal vasculature was associated with a poor prognosis. Inhibition of NO production reduced vascular permeability, preserved normal hematopoietic stem cell function, and improved treatment response in PDX.

## Highlights

• Functional imaging of the BM vascular niche using intravital 2P microscopy
• Increased vascular permeability is caused by robust molecular alterations in ECs
• Persistent high NO after chemotherapy is associated with poor prognosis in patients
• Inhibition of EC-derived NO normalizes vasculature and improve response to Ara.C

## Graphical Abstract

**Figure:**
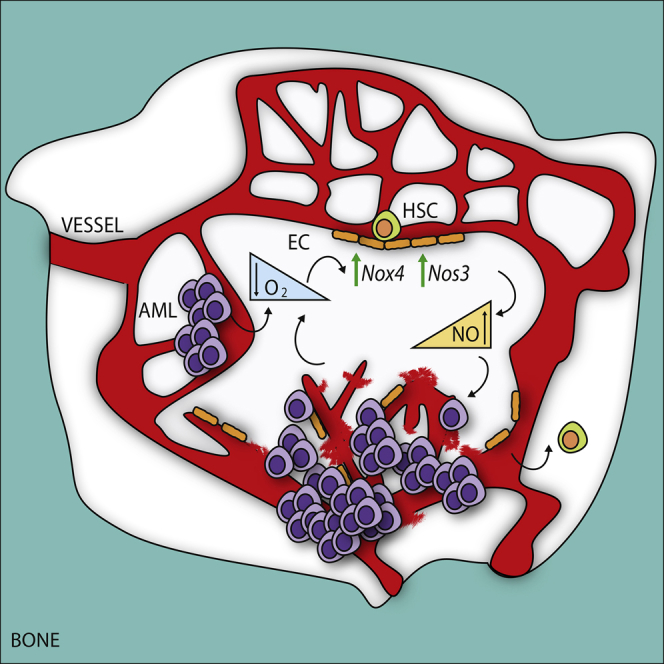

## Teaser

Passaro et al. show that acute myeloid leukemia, mainly via nitric oxide (NO), causes bone marrow vascular abnormalities and that failure to restore normal vasculature after induction chemotherapy is associated with a poor prognosis. Importantly, inhibition of NO production improves treatment response *in vivo*.

## Significance

**Acute myeloid leukemia (AML) represents the most common acute leukemia affecting adults. Recent findings suggest that AML cells alter their microenvironment, which represents an intriguing source of potential therapeutic targets. A thorough characterization of the vasculature of the bone marrow with AML reveals a severe functional abnormality. Vascular wall barriers responsible for oxygen, nutrients, and drug delivery appear severely damaged, with increased permeability, altered perfusion, and release of normal hematopoietic stem cells to the periphery. At a molecular level, endothelial cell signature is altered, resulting in increased reactive oxygen species and nitric oxide. In our preclinical models, the successful combination of nitric oxide synthase inhibitors and chemotherapy restore normal vasculature and delay leukemia, leading the way to combine leukemia-niche therapies in clinical trials.**

## Introduction

Acute myeloid leukemia (AML) is the most common acute leukemia in adults (Sant et al., 2010). AML is a hematological malignancy arising from the occurrence of genetic mutations in hematopoietic progenitors, which cause a blockage in the maturation and an uncontrolled growth of leukemic blasts in the bone marrow (BM). While the clinical presentation of AML is quite uniform, it is a highly heterogeneous disease at the molecular level. There is a consistent effort aimed to characterize each molecular subgroup and design the best therapeutic strategy (Grimwade et al., 2010). Besides a few cases (Wang and Chen, 2008), the common clinical practice remains induction therapy with cytarabine (AraC) (Buchner et al., 2012, Mayer et al., 1994). However, therapy resistance and relapse remain a major clinical challenge.

Another approach aims to identify and target common features within this complex disease. One possibility could be the BM microenvironment, which is the site where leukemic cells arise, expand, and eventually develop resistance to therapy. The BM microenvironment is constituted by different types of cells, with a predominant vascular component responsible for nutrient and metabolite turnover, the ingress and egress of different cells, and the regulation of normal hematopoietic stem cell (HSC) function (Boulais and Frenette, 2015, Mendez-Ferrer et al., 2015). The introduction and development of intravital two-photon (2P) microscopy has allowed direct, high-resolution, dynamic imaging of the calvarium BM (Colmone et al., 2008, Foster et al., 2015, Lassailly et al., 2013, Lo Celso et al., 2009, Sipkins et al., 2005), providing a powerful tool for the functional characterization of the BM microenvironment.

Recent findings indicate that myeloid malignancies also affect the function of the BM niche, pointing to the existence of an active crosstalk between leukemic cells and the microenvironment (Dong et al., 2016, Frisch et al., 2012, Hanoun et al., 2014, Kim et al., 2008, Kode et al., 2014, Krause et al., 2013, Raaijmakers et al., 2010, Schepers et al., 2013, Schmidt et al., 2011, Zhang et al., 2012). Similar to what has been observed in solid cancers, AML has been associated with an increase in microvascular density (MVD) and production of pro-angiogenic factors, notably vascular endothelial growth factor (VEGF) (Chand et al., 2016, Hussong et al., 2000, Kampen et al., 2013). However, whether increased MVD and VEGF constituted a prognostic factor for AML treatment response remains unclear (Aref et al., 2005, Padro et al., 2000, Reddy and Moreb, 2000). Moreover, despite promising preclinical studies (Zhu et al., 2003), clinical trials incorporating anti-VEGF inhibitors have not produced encouraging results (Fiedler et al., 2003, Fiedler et al., 2005, Giles et al., 2006, Mattison et al., 2015, Roboz et al., 2006, Zahiragic et al., 2007), suggesting that targeting pro-angiogenic cytokines may not be the best strategy for disrupting the crosstalk between AML and the vascular niche. A thorough analysis of the status of the vasculature in the BM is required to dissect the complexity of the vascular phenotype associated with AML. Thus, we aim to study the *in vivo* picture of the abnormalities associated with the BM vasculature induced by AML engraftment and unravel common pathologic processes, which could represent potential targets in AML.

## Results

### AML Engraftment Alters Vascular Architecture and Function

**Figure 1:**
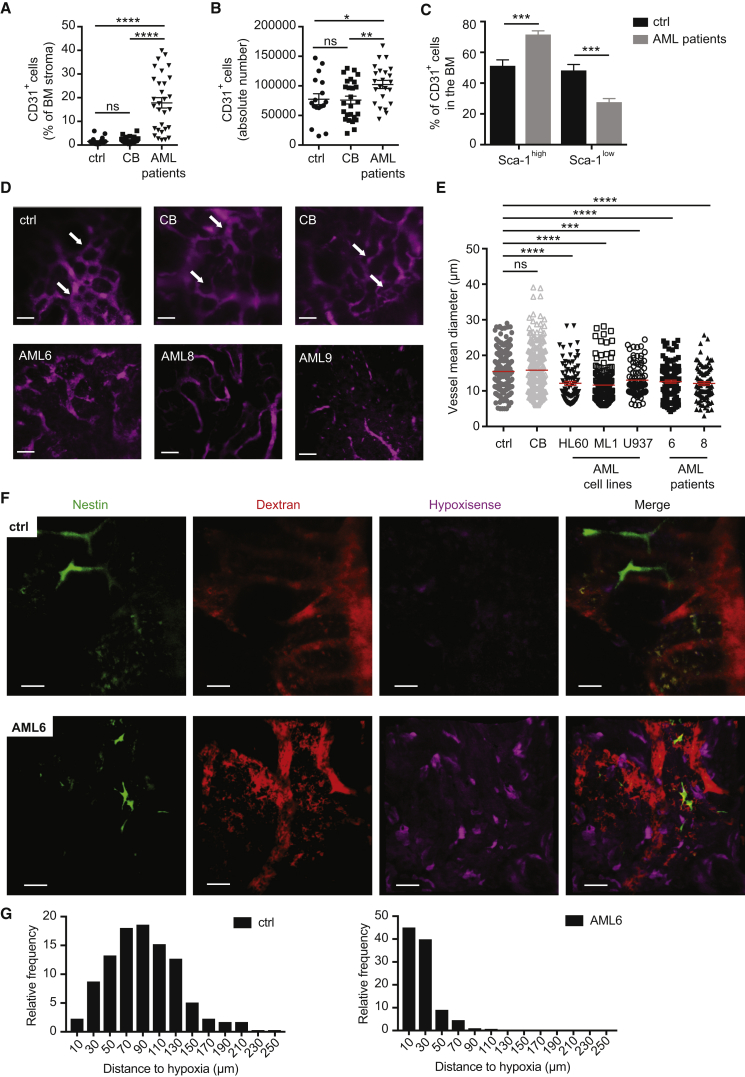
AML-Derived Alteration of the BM Vascular Architecture and Function (A) Quantification of CD31^+^ ECs in the BM (shown as percentage of CD45^-^Ter119^-^ BM cells) of non-transplanted mice (ctrl) and mice transplanted with HSPCs (CB) or AML patient-derived samples, as depicted. Each dot represents an individual mouse. Ctrl, n = 22; CB, n = 29; AML patients (AML1, 2, 3, 5, 6, 7, 8, 9), n = 30. Data are shown as mean ± SEM. (B) Absolute number of CD31^+^ ECs in the BM (2 femurs, 2 tibias, and 2 iliac crests) of non-transplanted mice (ctrl) and mice transplanted with CB-derived HSPCs (CB) or AML patient-derived samples, as depicted. Each dot represents an individual mouse. Ctrl, n = 17; CB, n = 26; AML patients (AML1, 2, 6, 7, 8, 9), n = 23. Data are shown as mean ± SEM. (C) Frequency of Sca-1^high^ and Sca-1^low^ CD31^+^ cells in the BM of non-transplanted mice (ctrl) and mice transplanted with AML patient-derived samples. Ctrl, n = 19; AML patients (AML 2, 3, 6, 8, 9), n = 16. Data are shown as mean ± SEM. (D) Representative 3D reconstruction of BM vasculature of the calvarium of non-transplanted mice (ctrl) and mice transplanted with CB-derived HSPCs (CB) or AML patient-derived samples, as depicted, imaged via 2P microscopy 1 min after injection of 655-conjugated NT-Qtracker as vessel-pooling agent. White arrows pointing at sinusoids. Data are representative of triplicates in 4 independent experiments. Scale bars represent 70 μm. (E) Quantification of vascular mean diameter in the calvarium BM of non-transplanted mice (ctrl) and mice transplanted with CB-derived HSPCs (CB) or human AML-derived samples, as depicted, using IMARIS filament tool. Dots represent the diameter of vascular fragments in the z stack of the calvarium of at least 3 mice per group. Ctrl, n = 235; CB, n = 232; HL60, n = 91; ML1, n = 148; U937, n = 85; AML6, n = 130; AML8, n = 94. Red lines represent the mean ± SEM. (F) Representative 3D reconstruction of BM hypoxia imaged via intravital microscopy using the HypoxiSense probe together with vasculature (dextran) and Nestin^+^ cells in non-transplanted mice or mice transplanted with AML6 patient-derived cells, as depicted. Scale bars represent 50 μm. (G) Distribution and relative frequency of vessel distances to hypoxic areas in the BM of non-transplanted mice (ctrl) or mice transplanted with AML6 patient-derived cells, as depicted. ns, not significant; ^∗^p < 0.05; ^∗∗^p < 0.01, ^∗∗∗^p < 0.001, ^∗∗∗∗^p < 0.0001. See also Figure S1.

To provide a detailed picture of the BM vasculature in AML, we studied the status of the vascular niche in human AML patient-derived xenografts (PDX). Recipient mice were left unconditioned, given the toxic effect derived from the irradiation or myelosuppressive treatment on the vasculature (Hooper et al., 2009, Kopp et al., 2005, Shirota and Tavassoli, 1991; and data not shown). We observed an expansion of the endothelial compartment among the non-hematopoietic stroma upon human AML engraftment (Figure 1A and Table 1). Importantly, this effect was specific to AML, as no such expansion was observed in mice engrafted with normal human hematopoietic stem/progenitor cells (HSPCs) derived from umbilical cord blood (CB) (Figure 1A). Moreover, the percentage of endothelial cells (ECs) was positively correlated to the leukemic engraftment of human AML cell lines and patient-derived samples (Figure S1A), suggesting a gradual pathologic process. Not only did the percentage of ECs increase, but there was also a real expansion of the endothelial compartment in terms of absolute number, specifically upon human AML engraftment (Figure 1B). We also observed an increased MVD, as shown by the higher number of vessel sprouts quantified by immunofluorescence (Figures S1B and S1C), similar to what is observed in patient-derived trephines (Chand et al., 2016, Padro et al., 2000). The existence of specific endothelial cell markers defining distinct BM vascular niches has recently been highlighted (Itkin et al., 2016). We thus analyzed the expression of these markers in the context of AML disease in PDX. We observed a significant loss of ECs associated with sinusoids (CD31^+^Sca1^low^) as well as an increased number of ECs associated with arterioles (CD31^+^Sca1^high^) (Figures 1C and S1D). We next analyzed the architecture of the BM vasculature by 2P microscopy using a vessel-pooling agent to visualize the vascular tree in the calvarium BM. Although vascular architecture appeared highly heterogeneous among different PDX (Figures 1D and S1E), we noticed some common abnormalities. First, the regularity of sinusoidal structures, which are preserved with normal human engraftment, was lost in human AML xenografts (Figure 1D, white arrows pointing at sinusoids). Second, the mean vascular diameter of vessels was reduced (Figure 1E), a pathologic phenotype previously reported in tumor angiogenesis as a result of solid stress applied to vessels by overgrowing tumor cells (Padera et al., 2004, Stylianopoulos and Jain, 2013). Vessel compression was also highlighted by H&E staining in long bones (Figure S1F, dashed circles indicating vessel lumen). To study BM perfusion, we injected isolectin B4 (IB4), a pan-endothelial marker (Lassailly et al., 2013), and analyzed its distribution on the BM vasculature by 2P microscopy. In control mice, we observed a homogeneous IB4 perfusion rate, allowing the visualization of ECs surrounding the arteriolar and sinusoidal vasculature (Figure S1G, ctrl). In contrast, we observed the presence of many poorly perfused areas in the BM of AML xenografts (Figure S1G). We next tested whether AML engraftment also affected BM oxygenation, by measuring the BM hypoxia. While in non-transplanted mice we observed a heterogeneous staining with Hypoxyprobe, indicating a physiological spread distribution of hypoxic areas, human AML engraftment increased the hypoxia homogeneously throughout the bones (Figure S1H). Quantification of Hypoxyprobe staining in BM cells by flow cytometry confirmed the significant increase of BM hypoxia upon human AML engraftment compared with normal human engraftment (Figure S1I). Of note, at early stage of engraftment hypoxia was localized in close proximity to AML cells (Figures S1J–S1M), whereas at high engraftment the BM was overall hypoxic (Figures S1N–S1P). We next visualized the hypoxic state of the BM via intravital microscopy using HypoxiSense probe (Bao et al., 2012). Similar to what was observed with Hypoxyprobe, we observed increased hypoxia in the BM upon AML engraftment with this alternative method (Figures 1F, S1Q, and S1R). To evaluate the hypoxic state of the vasculature, we measured the distance of each vessel to hypoxic areas. Whereas in control BM this distance was widely distributed, in the presence of AML most of the vessels were close to hypoxic areas (Figures 1G and S1S). Together these results showed that AML is associated with a structurally and functionally abnormal vasculature in the BM.

**Table 1:** Patient-Derived Samples Used for Xenograft Studies

AML ID	Cytogenetics	Cyto Risk	NPM	FLT3
AML1	ND^a^	ND	mutant	ND
AML2	t(9,6)	intermediate	ND	ND
AML3	normal	intermediate	WT	ITD^b^
AML4	11q23/MLL	ND	ND	ND
AML5	complex	poor	WT	ND
AML6	normal	intermediate	mutant	ITD
AML7	normal	intermediate	mutant	ITD
AML8	Del 7q+8 + 1	poor	WT	WT
AML9	normal	intermediate	mutant	ITD
AML10	complex	poor	ND	ND

a Not determined.

b Internal tandem duplication.

### AML Engraftment Increases Vascular Permeability in the BM

**Figure 2:**
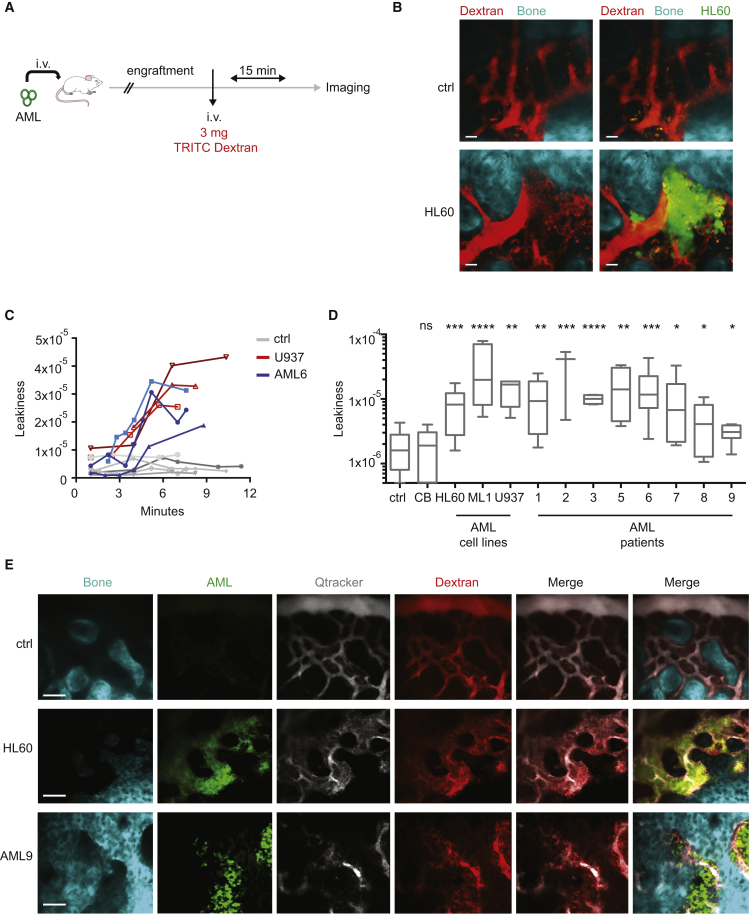
AML Engraftment Increases Vascular Permeability in the BM (A) Schematic of the experiment. Human AML cells engineered to express GFP and luciferase were injected intravenously into NSG mice and engraftment was monitored via bioluminescence and BM aspirate. Once engraftment was confirmed, 3 mg of 65–85 kDa TRITC-dextran was injected intravenously and 15 min later, calvaria were imaged via 2P microscopy. (B) Representative z stack of the TRITC-dextran-labeled BM vasculature in the calvarium of non-transplanted control or from mice transplanted with GFP-HL60 cells. Left: TRITC-dextran signal alone. Right: merge of TRITC-dextran and GFP signals. Data are representative of triplicates. Scale bars represent 30 μm. (C) Vascular leakiness quantified *in vivo* at different time points after dextran injection in non-transplanted mice (ctrl) and U937 or AML6 patient cells-engrafted mice. (D) Vascular leakiness quantification in the BM of non-transplanted mice or mice transplanted with different human AML cell lines and patient-derived samples, as depicted, and imaged 10 min after administration of vascular dyes. Ctrl, n = 11; CB, n = 19; HL60, n = 12; ML1, n = 7; U937, n = 4; AML9, n = 6; AML6, n = 14; AML5, n = 4; AML1, n = 12; AML3, n = 4; AML2, n = 3; AML8, n = 7; AML7, n = 6. Data are shown as whiskers minimum-to-maximum plots, the line inside the box representing the mean, the top and the bottom lines representing the 75% and 25% percentiles, respectively, and the lines above and below the box representing the SD. (E) Representative z stacks of BM vasculature in the calvarium of non-transplanted mice (ctrl) or mice engrafted with HL60 or AML9 cells, as depicted. Scale bars represent 80 μm. ns, not significant; ^∗^p < 0.05, ^∗∗^p < 0.01, ^∗∗∗^p < 0.001, ^∗∗∗∗^p < 0.0001. See also Figure S2.

To study the vascular barrier function in the BM of human AML xenografts, we used intravital 2P microscopy to image the calvarium of unconditioned NSG mice engrafted with different human AML cell lines or patient-derived samples. Human AML cell lines were engineered to express GFP and luciferase to facilitate the monitoring of BM engraftment via bioluminescence (Figure S2A) and BM aspirate (Figure S2B). Prior to imaging, tetramethylrhodamine isothiocyanate (TRITC)-dextran was injected intravenously to label the vasculature (Egawa et al., 2013, Fukumura and Jain, 2008) (Figure 2A). Strikingly, we observed an important leakage of the dextran outside the vasculature in the BM of mice engrafted with human AML cell lines compared with control non-transplanted mice (Figures 2B and S2C). This observation was corroborated by H&E staining of long bones showing the loss of vascular barriers and release of erythrocytes in the BM parenchyma (Figure S2D, red arrows pointing at erythrocytes). To quantify the vascular leakiness, we used a strategy allowing reliable measurement independent of the fluorescence intensity of the probe (detailed in Figure S2E and STAR Methods). The intravital observation of the calvarium BM vasculature at different time points after injection of the vessel-pooling agents showed an increased vascular leakiness starting between 6 and 9 min post injection (Figure 2C). We quantified vascular permeability in xenografts of different human AML samples 10 min after injection of the vessel-pooling agents (Figures 2D and 2E). All the samples used in our study, which represented different molecular subtypes of human AML, induced increased vascular leakiness in the BM. A different method using *in vivo* Hoechst permeability in BM cells (Wong et al., 2015) was used to confirm the AML-associated vascular phenotype in the BM (Figures S2F and S2G). Of note, AML-engrafted areas appeared to be much more leaky than non-engrafted areas (Figures S2H and S2I). In normal BM, Nestin-associated vessels, characterized by higher expression of Sca-1 (Itkin et al., 2016), show less permeability than Nestin^−^ vessels (Figure S2J). On the contrary, in the presence of leukemic engraftment, both Nestin^+^ and Nestin^−^ vessels were associated with increased leakiness (Figures S2J and S2K), highlighting a substantial functional difference between normal and leukemic Nestin-associated Sca-1^+^ vessels. Of note, reduction of normal pericyte coverage was also observed in the BM of AML PDX (Figure S2L). These results show an altered vascular permeability in the BM associated with AML engraftment, observed commonly throughout different AML subtypes.

### Chemotherapy Does Not Rescue Normal Vascular Permeability in the BM

**Figure 3:**
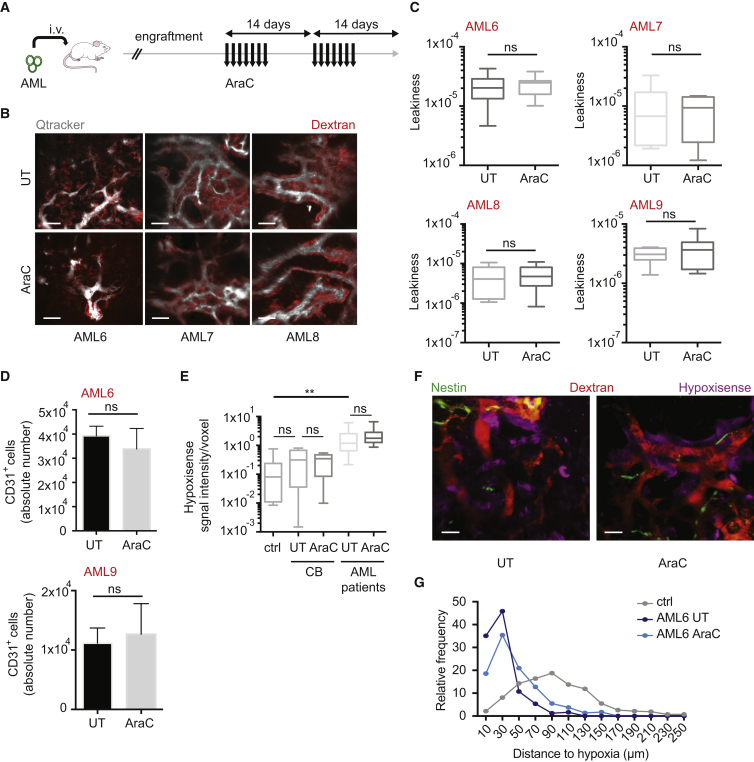
Persistence of Increased Vascular Permeability after Chemotherapy (A) Schematic of the experiment. Patient-derived AML cells were injected intravenously into NSG mice and engraftment was monitored via BM aspirate. Once BM engraftment (>40%) assessed, 10 mg/kg/day of AraC or solvent was administrated subcutaneously for one or two alternate weeks, depending on the patient sample. Seven days after the last administration of AraC, vessel-pooling agents were administrated intravenously. Mice were euthanized 10 min later and calvaria imaged via 2P microscopy. (B) Representative 3D reconstruction of the BM vasculature in the calvarium of mice engrafted with different patient-derived samples treated with solvent (top) or AraC (bottom). Scale bars represent 70 μm. (C) Vascular leakiness in the BM of mice transplanted with different human AML patient-derived samples, treated with solvent or AraC, as depicted. Three or more replicates were used for each condition. Data are shown as whiskers minimum-to-maximum plots, the line inside the box representing the mean, the top and the bottom lines representing the 75% and 25% percentiles, respectively, and the lines above and below the box representing the SD. (D) Absolute number of CD31^+^ ECs in the BM (n = 2 femurs) of mice transplanted with AML6 (top) or AML9 (bottom) patient-derived cells, treated with solvent or AraC, as depicted; n = 3 in each condition. Data are shown as mean ± SEM. (E) Hypoxia represented as HypoxiSense signal intensity/voxel in the calvaria BM of non-transplanted mice (ctrl) and mice transplanted with CB-derived HSPCs (CB) or AML patient-derived samples, treated or not with AraC, as depicted. Ctrl, n = 9; CB UT, n = 7; CB AraC, n = 8; AML patients (AML6 and 9) UT, n = 16; AraC, n = 16. Data are shown as whiskers minimum-to-maximum plots, the line inside the box representing the mean, the top and the bottom line representing the 75% and 25% percentiles, respectively, and the lines above and below the box representing the SD. (F) Representative 3D reconstruction of BM hypoxia imaged via intravital microscopy using the HypoxiSense probe together with vasculature (dextran) and Nestin^+^ cells in mice transplanted with AML6 patient-derived cells and treated or not with AraC, as depicted. Scale bars represent 50 μm. (G) Distribution and relative frequency of vessel distances to hypoxic areas in the BM of mice transplanted with AML6 patient-derived cells, and treated or not with AraC, as depicted. ns, not significant; ^∗∗^p < 0.01. See also Figure S3; Tables S1 and S2.

It is still debated whether AML-derived angiogenesis, in terms of MVD in the BM and VEGF quantification, is reduced in patients upon chemotherapy (Aref et al., 2005, Chand et al., 2016, Padro et al., 2000, Reddy and Moreb, 2000). Thus, we analyzed the level of VEGF in BM biopsies obtained at diagnosis and post treatment from a cohort of AML patients (Tables S1 and S2). We found an increase in VEGF in the majority of patients' post-treatment biopsies (Figures S3A and S3B). This suggests a persistent pro-angiogenic environment after chemotherapy. We thus studied the vascular niche state in AML PDX after chemotherapy. We set up a preclinical treatment protocol, which closely followed the clinical practice, by treating AML xenotransplanted mice daily with 10 mg/kg of AraC during one or two alternate weeks, depending on xenografts' response (Figure 3A). This treatment significantly reduced the leukemic burden in the BM (Figure S3C) and induced a “remission-like” phase, during which the remaining leukemic cells persist and give rise to relapse after few weeks (not shown). Notably, residual AML cells after AraC treatment localized in close proximity to the vasculature (Figure S3D). We observed the BM vasculature with 2P microscopy during the “remission-like” phase in different cohorts of AML PDX treated with this protocol. Although the leukemic engraftment was significantly reduced, the vascular permeability was still very high, similar to mice treated with the control solvent, which were highly engrafted with leukemic cells (Figures 3B and 3C). Moreover, the number of ECs was not reduced in AML PDX after AraC treatment (Figure 3D), suggesting that the abnormal vascular phenotype was maintained. This effect was not due to an unspecific toxicity of the chemotherapy on the vascular niche, as non-transplanted mice or mice engrafted with normal HSPCs and subjected to the same treatment did not show abnormal vascular permeability (Figures S3E and S3F), frequency, or number of ECs in the BM (Figures S3G and S3H). These results show that in AML disease, the vascular barrier functionality is still compromised after chemotherapeutic treatment. Furthermore, we observed that BM hypoxia was not normalized upon removal of leukemic cells (Figures 3E and 3F). Notably, similarly to what was observed in presence of high leukemic infiltration, most of the vessels were still close to hypoxic areas upon AraC treatment (Figure 3G). These data highlight a persistent poorly functional vasculature in the BM of AML PDX after chemotherapy.

### AML-Induced Molecular Signature in Vascular ECs

**Figure 4:**
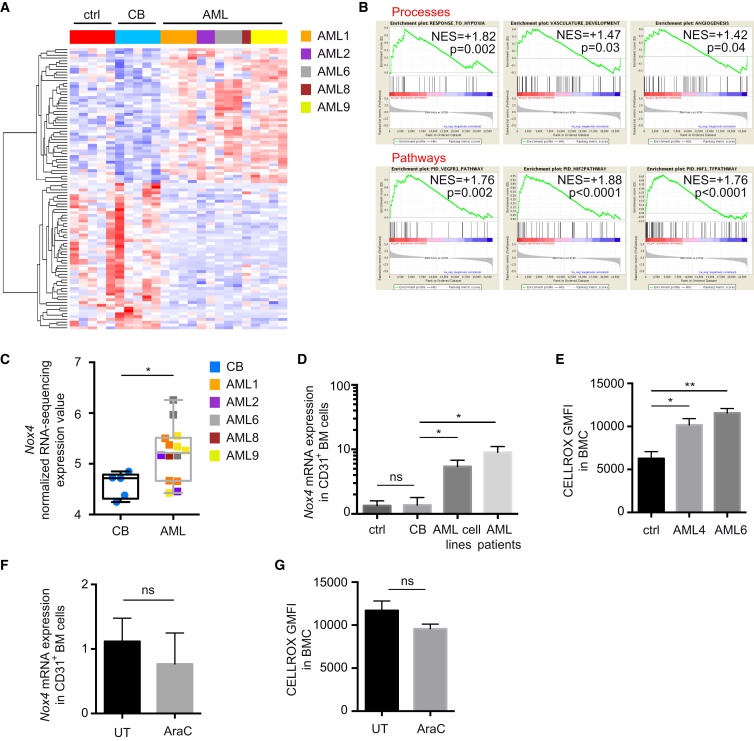
AML-Induced Transcriptional Signature in BM ECs (A) Heatmap from hierarchical clustering of GSEA top 100 ranked genes between untransplanted mice (ctrl group), mice engrafted with human CB-derived HSPCs (CB group), and mice engrafted with different patient-derived samples (AML group) derived BM ECs, using RNA-seq data. Red indicates higher expression values and blue indicates lower expression values (log_2_ FPKM [fragments per kilobase per million mapped] scale). (B) Enrichment plots for Processes (top) and Pathways (bottom) from GSEA between CB and AML groups using RNA-seq gene expression data indicate enrichment of these pathways in AML group. Normalized enrichment score (NES) and nominal p value are shown. (C) Normalized expression values of *Nox4* in CB versus AML groups analyzed via RNA-seq. Data are shown as whiskers minimum-to-maximum plots, the line inside the box representing the mean, the top and the bottom lines representing the 75% and 25% percentiles, respectively, and the lines above and below the box representing the SD. (D) *Nox4* expression analyzed by qRT-PCR in CD31^+^ BM cells retrieved from the depicted groups of mice. CB, n = 5; AML cell lines, n = 8; AML patients (AML1, 2, 9), n = 13. Data are shown as mean ± SEM. (E) Cellular ROS levels in BMC retrieved from mice of depicted groups; n = 3 per group. Data are shown as mean ± SEM. (F) *Nox4* expression analyzed by qRT-PCR in CD31^+^ BM cells retrieved from mice engrafted with AML6 and treated or not with AraC. n = 3 per group. Data are shown as mean ± SEM. (G) Cellular ROS levels in BMC retrieved from mice engrafted with AML6 and treated or not with AraC; n = 3 per group. Data are shown as mean ± SEM. ns, not significant; ^∗^p < 0.05, ^∗∗^p < 0.01. See also Figure S4.

AML cells have been extensively studied at the transcriptional level, with many reports assessing the production of angiogenic factors (Aref et al., 2002, Hatfield et al., 2009, Ribatti et al., 2007). To identify common downstream effectors of this angiogenic stimulation in the vascular niche, we studied the transcriptome of BM-derived ECs retrieved from mice engrafted with different AML patient-derived cells via RNA sequencing (RNA-seq), and compared it with ECs retrieved from non-engrafted mice or mice engrafted with normal HSPCs. As shown by the hierarchical clustering, despite the heterogeneity found among the groups, we identified a common deregulated signature associated with human AML engraftment (Figure 4A). Gene set enrichment analysis (GSEA) highlighted several altered processes directly associated with the abnormal vascular phenotype observed above, including vasculature development, angiogenesis, and response to hypoxia (Figure 4B). Several deregulated pathways associated with angiogenic and hypoxic stimulation were identified (Figures 4B and S4A). Interestingly, the expression of adhesion molecules changed dramatically upon AML engraftment, with upregulation of integrins associated with Fak pathway activation in ECs (Figure S4B) inducing migration, growth, and survival of newly forming vessels during angiogenesis (Muether et al., 2007, Stenzel et al., 2011, Weis and Cheresh, 2011), as well as initiation of VEGF-mediated vascular permeability (Chen et al., 2012, Eliceiri et al., 2002, Rodrigues and Granger, 2015). We noticed also reduced expression of tight junction components, e.g., *Cldn1* responsible for maintaining endothelial layer integrity (Rodrigues and Granger, 2015) or *Vcam1* required for HSC homing and retention to the niche (Lewandowski et al., 2010, Ulyanova et al., 2007). Interestingly, we found among the common highest upregulated genes in ECs upon AML engraftment *Nox4*, a NAPDH oxidase expressed in the vasculature and involved in the response to hypoxia via production of reactive oxygen species (ROS), activation of nitric oxide synthase 3 (NOS3), and release of nitric oxide (NO) (Craige et al., 2011) (Figure 4C). Of note, in response to AML engraftment, *Nox4* expression in ECs (Figure 4D) and ROS production in the BM (Figure 4E) were both increased. Moreover, the upregulated expression of *Nox4* in ECs and the high production of ROS in the BM were maintained after AraC treatment (Figures 4F and 4G). These data show that the AML-induced hypoxic environment alters the molecular signature of vascular ECs, activating several pro-angiogenic pathways and positively regulating *Nox4* expression and the response to hypoxia.

### AML Engraftment Is Associated with Increased Nitric Oxide Levels in the BM

**Figure 5:**
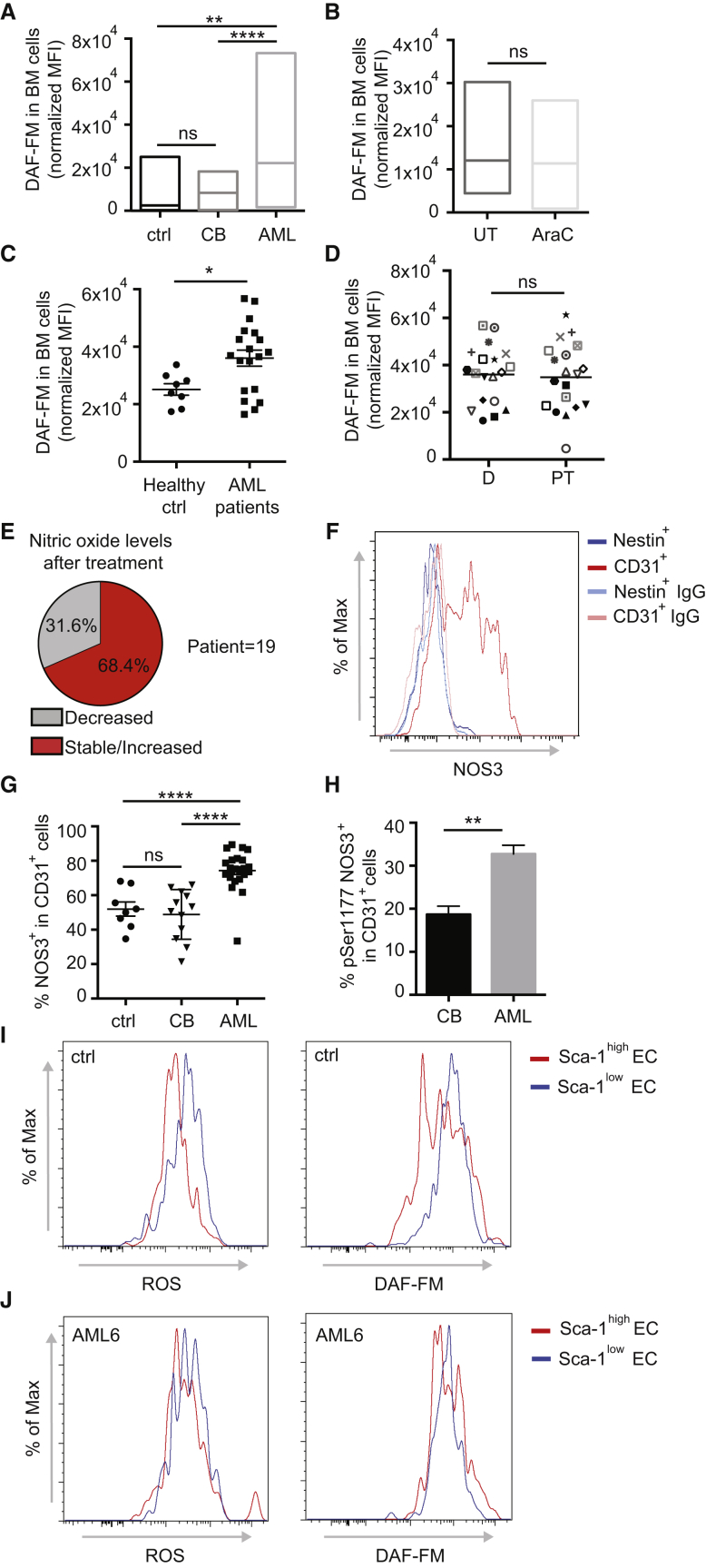
AML Engraftment Is Associated with Increased Nitric Oxide Levels in the BM (A) Nitric oxide levels shown as DAF fluorescence intensity (normalized mean fluorescence intensity [MFI]) in BM cells of non-transplanted mice (ctrl) and mice transplanted with CB-derived HSPCs (CB) or AML patient-derived samples, as depicted. Ctrl, n = 13; CB, n = 24; AML patients (AML1, 2, 6, 8, 9), n = 35. Data are shown as minimum-to-maximum box plots, the line inside the box representing the mean. (B) Nitric oxide levels (normalized MFI) in BM cells of mice transplanted with AML patient-derived cells, treated with solvent or AraC, as depicted. AML patients: AML6 and 9. UT, n = 7; AraC, n = 7. Data are shown as minimum-to-maximum box plots, the line inside the box representing the mean. (C) Nitric oxide levels in BM cells of healthy donors or AML patients at diagnosis. Healthy ctrl, n = 8; AML patients, n = 19. Error bars represent mean ± SEM. (D) Nitric oxide levels in BM cells of AML patients at diagnosis (D) and post treatment (PT); n = 18. Error bars represent mean. (E) Pie chart showing the percentage of patients with decreased or stable/increased levels of nitric oxide after treatment. (F) NOS3 expression measured via flow cytometry in Nestin^+^ and CD31^+^ cells, as depicted. Data are representative of triplicates. (G) Percentage of NOS3^+^ ECs measured by flow cytometry in the BM of non-transplanted mice and mice engrafted with CB-derived HSPCs (CB) or AML patient-derived samples, as depicted. Ctrl, n = 8; CB, n = 12; AML patients (AML2, 6, 8, 9), n = 24. Data are shown as mean ± SEM. (H) Activated NOS3 (measured by flow cytometry with an anti-phosphoSer1177 antibody) in CD31^+^ ECs of the BM of mice engrafted with CB-derived CD34^+^ cells (CB) or AML patient-derived samples, as depicted. CB, n = 4; AML patients (AML2, 6, 9), n = 9. Data are shown as mean ± SEM. (I) Cellular ROS (left) and nitric oxide (right) levels in different populations of BM-derived ECs, as depicted, in non-transplanted mice (ctrl). Data are representative of triplicates. (J) Cellular ROS (left) and nitric oxide (right) levels in different populations of BM-derived ECs, as depicted, in mice engrafted with AML6 patient-derived cells. Data are representative of triplicates. ns, not significant; ^∗^p < 0.05, ^∗∗^p < 0.01, ^∗∗∗∗^p < 0.0001. See also Figure S5.

As NO is the major mediator of vascular permeability in both physiological and several pathological conditions (Aramoto et al., 2004, Fukumura et al., 2001, Fukumura et al., 2006, Mayhan, 1999), we investigated whether the AML-derived upregulation of *Nox4* in ECs influenced NO production in the BM vascular niche. We found increased levels of NO in the BM of AML xenografts compared with non-transplanted mice or mice engrafted with normal HSPCs (Figure 5A). A slight but significant increase of NO levels was also observed in BM of PDX with low engraftment (not shown). This effect seemed to be specific to BM (Figure S5A). Of note, as already observed for vascular permeability and *Nox4* expression in ECs, chemotherapy failed to normalize the NO levels in the BM of AML PDX (Figure 5B).

To translate our observations to the human disease, we analyzed the NO levels in our cohort of AML patient-derived BM biopsies and compared them with those of healthy donors (Tables S1 and S2). We observed a significant increase in the NO level in BM aspirates of patients at diagnosis, which were similar to those observed in xenografts, compared with healthy donors (Figure 5C). NO levels were higher in the BM compared with the blood of the same patient at diagnosis (Figure S5B), similar to what was observed in mice with AML xenografts. Importantly, also similar to what was observed in AML xenografts, the NO levels remained elevated in the majority of post-treatment BM samples analyzed (Figures 5D and 5E). Moreover, failure in reducing NO levels appeared to be associated with a higher incidence of treatment failure (Figure S5C), with a probability of 73% in the cohort of patients studied herein (Figure S5D). This probability increases to 100% if we restrict the analysis to the intermediate-risk group samples, characterized by a more controversial prognostic assessment (Figure S5E). Our data suggest that persistence of high NO levels in the BM of patients after therapy could represent a biomarker for poor prognosis, particularly in patients with intermediate-risk AML. The upregulation of *Nox4* in ECs, indicating an overactivation of NOS3 in this compartment, likely is responsible for the increased NO levels in the BM. We therefore analyzed the expression of NOS3 in the two major components of the vascular niche, endothelial (CD31^+^) and mesenchymal perivascular (Nestin^+^) cells (Figure S5F). We observed that ECs represented the major compartment expressing NOS3 both at mRNA (Figure S5G) and protein (Figure 5F) levels. Moreover, we observed that NOS3 protein expression was significantly increased in ECs retrieved from mice engrafted with human AML patient-derived samples (Figure 5G). Of note, not only the total NOS3 protein level but also its activation was increased, as shown by increased phosphorylation of Ser1177 (Figure 5H (Kolluru et al., 2010)). In contrast, NOS3 was not increased in the mesenchymal compartment upon AML engraftment (Figure S5H). NOS2 protein expression is not changed in either the endothelial or the mesenchymal compartment upon AML engraftment (Figure S5I). Analysis of different endothelial subpopulations revealed that in normal BM, CD31^+^Sca-1^high^ ECs exhibit lower ROS and NO levels than CD31^+^Sca-1^low^ ECs (Figure 5I), and AML engraftment caused an increase in both ROS and NO levels in the CD31^+^Sca-1^high^ population (Figure 5J). We investigated the consequences of long-term higher production of NO and ROS in ECs, and observed that in heavily engrafted AML xenografts ECs displayed increased apoptosis in both Sca-1^high^ and Sca-1^low^ ECs (Figure S5J), whereas cell-cycle arrest was restricted to the Sca-1^high^ EC fraction (Figure S5K). We next evaluated whether the leukemic compartment might also contribute to the NO overproduction. Human AML cells do not express higher mRNA levels of *NOS2* or *NOS3* compared with normal hematopoietic cells residing in the BM, as reported in publicly available datasets (HemaExplorer/BloodSpot, not shown). Moreover, the mRNA levels of these enzymes in AML patient-derived samples were similar to those from healthy donors (Figures S5L and S5M). There was no significant upregulation of these enzymes after chemotherapy (Figures S5N and S5O), or in AML xenografts (P1) compared with their primary counterpart (P0) (Figure S5P). The NOS3 protein level in AML was much lower than that in the endothelial compartment (Figure S5Q). Our results show an overproduction of NO in the BM in the presence of AML at diagnosis as well as after therapy, produced by an abnormally activated NOS3 in BM ECs.

### Targeting Vascular Permeability Cooperates with Chemotherapy to Improve AML Treatment Response

**Figure 6:**
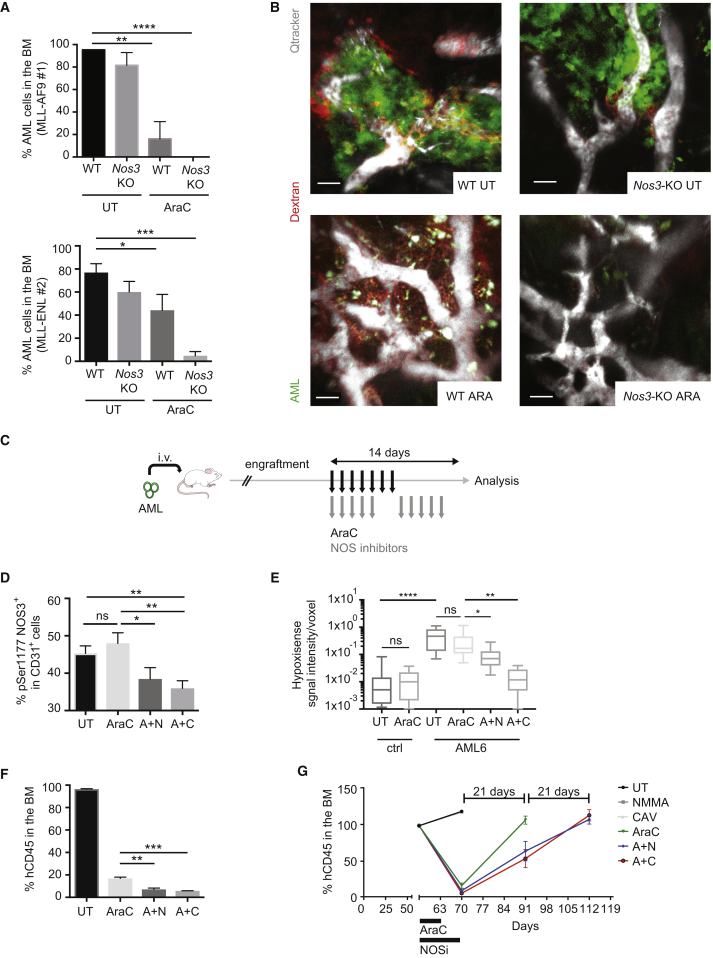
Targeting Vascular Permeability Cooperates with Chemotherapy to Improve AML Treatment (A) Percentage of murine GFP^+^ MLL-AF9 #1 or tomato^+^ MLL-ENL #2 AML cells in the BM of secondary mice of depicted genotypes, treated with solvent or AraC; n = 3 or more replicates. Data are shown as mean ± SEM. (B) Representative 3D reconstruction of calvarium BM of mice of depicted genotypes transplanted with murine MLL-AF9 #1 AML cells, treated with solvent or AraC. Scale bars represent 30 μm. (C) Schematic of the experiment. Mice engrafted with human AML patients' samples were treated with AraC or solvent for 1 week, and with or without NOS inhibitors for 2 weeks. (D) Activated NOS3 protein (measured by flow cytometry with an anti-phosphoSer1177 antibody) in CD31^+^ endothelial cells of the BM of mice transplanted with AML6 and AML9 patient-derived cells, treated as described in (C); n = 6. Data are shown as mean ± SEM. (E) Hypoxia represented as HypoxiSense signal intensity/voxel in the calvaria BM of non-transplanted mice (ctrl) and mice transplanted with AML6 patient-derived samples, treated or not with AraC, as depicted. Ctrl UT, n = 13; ctrl AraC, n = 10; AML6 UT, n = 18; AML6 AraC, n = 20; AML6 A + N, n = 9; AML6 A + C, n = 9. Data are shown as whiskers minimum-to-maximum plots, the line inside the box representing the mean, the top and the bottom lines representing the 75% and 25% percentiles, respectively, and the lines above and below the box representing the SD. (F) Percentage of human CD45^+^ engraftment in the BM of mice engrafted with patient-derived sample AML6 and treated as in (C); n = 3 per condition. Data are shown as mean ± SEM. (G) Percentage of human CD45^+^ engraftment measured via BM aspirate in mice engrafted with AML 6 and treated as in (C). Data are shown as mean ± SEM. ns, not significant; ^∗^p < 0.05, ^∗∗^p < 0.01, ^∗∗∗^p < 0.001, ^∗∗∗∗^p < 0.0001. See also Figure S6.

To test whether the exogenous production of NO has a role in leukemia transplantation and progression, we transplanted leukemic cells from two models of murine AML (MLL-AF9 and MLL-ENL) in *Nos3*-knockout (*Nos3*-KO) recipient mice and studied the status of the vasculature and disease progression. Interestingly, MLL-AF9 leukemia occurrence was significantly delayed in *Nos3*-KO mice (Figure S6A, top), *Nos3-KO* mice were active and healthy at the time wild-type (WT) mice showed clear signs of disease and were euthanized. Similar results were obtained using two distinct clones of MLL-ENL AML transplanted in WT or *Nos3*-KO recipient mice, showing significantly reduced disease penetrance (Figure S6A, bottom), reduced leukemic engraftment in the BM and spleen (Figures S6B and S6C), and amelioration of the anemic phenotype caused by AML (Figure S6D). Moreover, *Nos3* inactivation cooperated with AraC treatment to reduce leukemic engraftment in both MLL-AF9 and MLL-ENL models (Figure 6A) and reduced the vascular leakiness in the BM of AML-engrafted mice (Figure 6B). Together these data suggest that exogenous NO production by NOS3 has a role in AML engraftment. We next investigated whether the observed difference was likely to be attributed to a delay in engraftment or also to a reduction in disease progression. For that we used the PDX model and allowed AML cells to engraft the animals before inhibiting NO production with NOS inhibitors. We set up a similar treatment strategy to the one used before, including NOS inhibitors during and after the chemotherapy, to normalize the vascular permeability (Figure 6C). Treatment of mice xenografted with human ML1 cell line with NOS inhibitors in combination with chemotherapy significantly inhibited leukemic progression, as shown by reduced number of leukemic cells in the BM and the spleen (Figures S6E and S6F). The sole administration of NO inhibitors in heavily engrafted leukemic mice did not produce any significant decrease of leukemic load in the BM (Figure S6E), likely due to the absence of a specific effect of NO on leukemic cells. Indeed, treatment of leukemic cells (ML1 or primary patient samples) *in vitro* with NO inhibitor or NO donor did not affect their survival or proliferation ability (Figures S6G and S6H). NO inhibition combined with AraC treatment reduced NOS3 activation (Figure 6D), vascular leakiness (Figure S6I), and the BM hypoxia (Figure 6E). Importantly, the combined treatment was more powerful than the chemotherapy alone in reducing leukemic progression in the BM (Figures 6F and S6J) and the spleen (Figure S6K) of mice engrafted with two different patient-derived samples (Figure S6L). The combined treatment also significantly extended the “remission-like” phase of the disease compared with chemotherapy alone (Figure 6G). These data show that increased NO levels contribute to the vascular leakiness in AML-engrafted mice, and restoring normal vascular function in the BM by NOS inhibition improves the therapeutic response compared with standard chemotherapy.

### NOS Inhibition Potentiates Normal HSPC Function

**Figure 7:**
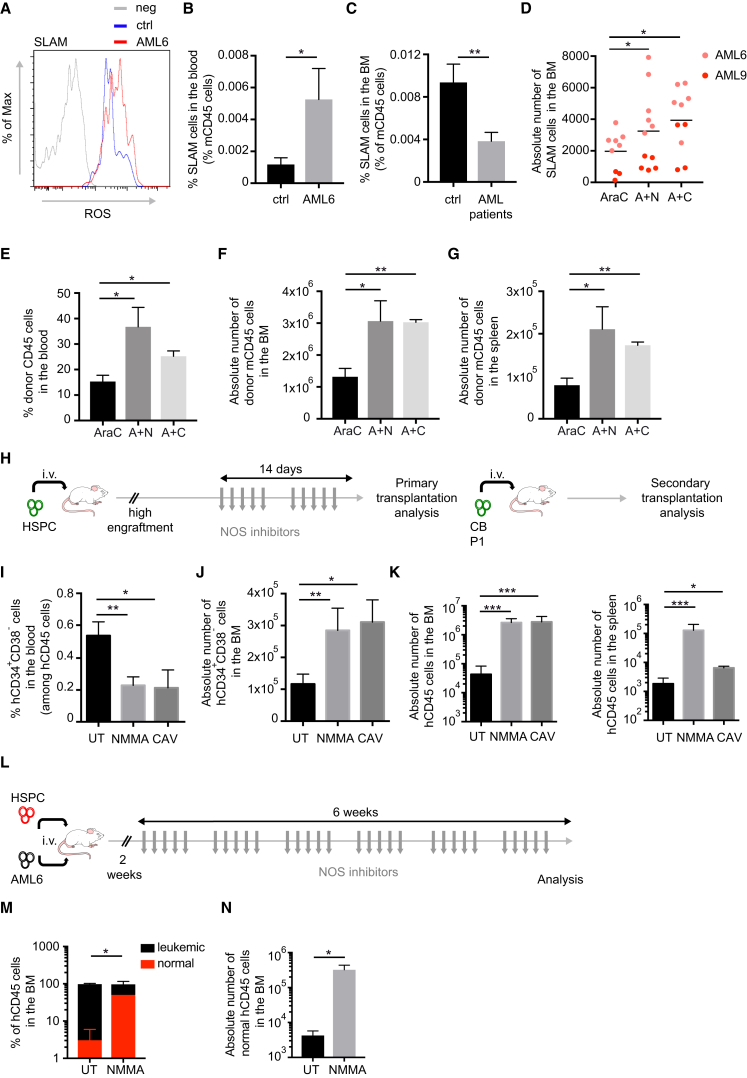
Targeting Vascular Permeability Potentiates HSPC Function (A) Cellular ROS levels in BM-derived HSC of non-transplanted mice or mice engrafted with AML6 patient-derived cells. Data are representative of triplicates. (B) Percentage of murine HSC among total murine CD45^+^ cells in the blood of non-transplanted mice and mice engrafted with AML6 patient-derived cells. ctrl, n = 5; AML6, n = 3. Data are shown as mean ± SEM. (C) Percentage of murine HSC among total murine CD45^+^ cells in the BM of non-transplanted mice of mice engrafted with AML patient-derived samples (AML6, 9). ctrl, n = 5; AML patients, n = 12. Data are shown as mean ± SEM. (D) Absolute number of murine HSC in the BM of mice transplanted with AML patient-derived cells, as depicted, and treated with AraC alone or in combination with NOS inhibitors. Each dot represents a mouse. Error bars represent mean. (E–G) Percentage in blood (E) and absolute number in BM (F) and spleen (G) of donor CD45.1, retrieved from NSG mice transplanted with AML6 patient-derived cells and treated with AraC alone or in combination with NOS inhibitors, injected in secondary CD45.2 *Rag2*-KO/*Gammac*-KO mice. AraC, n = 4; A + N, n = 3; A + C, n = 3. Data are shown as mean ± SEM. (H) Schematic of the experiment. Mice engrafted with human CD34^+^ CB cells were treated with NOS inhibitors (N^G^-monomethyl-L-arginine [NMMA] 20 mg/kg/day intraperitoneally or cavtratin 2 mg/kg/day intraperitoneally) for 2 weeks, 5 days/week. Mice were euthanized and BMC harvested for subsequent analysis and secondary transplantation. (I) Percentage of HSPCs in the peripheral blood of mice engrafted with CB-derived HSPCs and treated or not with NMMA or cavtratin. Data are shown as mean ± SEM. (J) Absolute number of HSPC in the BM of mice engrafted with CB-derived HSPC cells and treated or not with NMMA or cavtratin. Data are shown as mean ± SEM. (K) Absolute number of human CD45^+^ cells in the BM (left) and spleen (right) of secondary NSG mice transplanted with primary xenografts described in (H). Data are shown as mean ± SEM. (L) Schematic of the experiment. Non-irradiated NBSGW mice were simultaneously transplanted with tomato^+^ HSPCs (red) and non-transduced AML6 (black) patient-derived cells, and 2 weeks after transplantation control solvent or NMMA were administered for 6 weeks, 5 days/week. Two days after the last treatment, mice were euthanized and BM was analyzed. (M) Percentage of tomato^+^ (normal, red) and tomato^−^ (leukemic, black) cells among hCD45^+^ in the BM of mice described in (L); n = 4. Data are shown as mean ± SEM. (N) Absolute number of tomato^+^ hCD45^+^ (normal) cells in the BM of mice described in (L). Data are shown as mean ± SEM. ns, not significant; ^∗^p < 0.05, ^∗∗^p < 0.01, ^∗∗∗^p < 0.001. See also Figure S7.

Increased levels of NO have been associated with increased HSPC motility and egress from the BM, leading to a lower repopulation capacity of the BM stem cell pool after injuries (Aicher et al., 2003, Gur-Cohen et al., 2015). Moreover, recent findings indicate that loss of vascular integrity promotes HSPC mobilization and loss of the HSC pool in the BM (Itkin et al., 2016). We thus speculated that increased NO and vascular leakiness in AML could affect normal HSC. In the BM of AML xenografts, the residual murine HSC displayed higher levels of ROS (Figure 7A) and were mobilized to the periphery, as shown by increased SLAM^+^ cells in the blood of mice with AML xenografts compared with those without, as well as a reduced proportion of HSC in the BM (Figures 7B and 7C). The absolute number of SLAM^+^ cells in the BM was also decreased in mice with AML xenografts and in mice transplanted with murine MLL-ENL leukemic cells (Figures S7A and S7B). Therefore, we tested whether normalization of the vascular niche with NOS inhibitors would be beneficial for normal HSC function. We observed that NOS inhibitors in combination with AraC treatment were more efficient than chemotherapy alone in re-establishing the normal number of SLAM^+^ cells in the BM of PDX (Figure 7D). Not only the number but also the stem cell function of these SLAM^+^ cells was increased, as shown by their repopulation ability in secondary recipients (Figures S7C and 7E–7G). These data suggest that overproduction of NO by altered vascular niche affects normal residual HSC in the BM and that NOS inhibition may improve AML therapy by restoring normal stem cell function. We next tested the effect of NOS inhibitors on HSPCs. Hence, we treated mice engrafted with normal HSPCs with NOS inhibitors and analyzed the effect on the stem cell pool (Figure 7H). Interestingly, we observed reduced HSPC egress to the blood (Figure 7I), an effect specific for this compartment as no significant difference was observed on the egress of total hCD45^+^ cells into the blood (Figure S7D). This was accompanied by a specific increase of HSPCs in the BM (Figures 7J and S7E). This effect was not due to an increase in proliferation of the stem pool, as shown by cell-cycle analysis (Figure S7F), thus excluding a possible exhaustion of the compartment. Indeed, NO inhibition not only increased the number of HSPCs in the BM but also their stem cell function, as shown by their increased repopulation ability in secondary transplantation assays (Figure 7K). We next tested whether NO inhibition would be beneficial for residual normal HSPC to outcompete AML cells during the relapse process. To achieve this, and mimic the competition between AML cells and normal HSCs into the BM niche, we injected simultaneously normal human CB-derived HSPCs and AML patient-derived cells intravenously into non-irradiated NBSGW mice (McIntosh et al., 2015) and monitored the normal versus malignant engraftment in the presence or absence of long-term NOS inhibitors (Figure 7L). Analysis of the BM engraftment at the end of the treatment showed that NOS inhibition favored the normal over leukemic engraftment in the BM (Figures 7M and 7N). Together, these results suggest that the use of NO inhibitors will not only reverse the vascular permeability observed during AML progression and thus improve chemotherapy efficiency, but will also restore a normal stem cell vascular niche, likely protecting normal residual HSCs and allowing them to outcompete the leukemic cells.

Altogether, our results provide strong evidence for the importance of the alteration of the vascular niche in AML progression and relapse, via increased NO production by ECs, and call for clinical trials incorporating NOS inhibitors to target the abnormal vascular niche and improve the treatment response.

## Discussion

Tumor angiogenesis is a pathological complication of several types of solid cancers, which has been extensively studied and characterized. The angiogenic process is mostly initiated by pro-angiogenic factors derived from tumor cells, which in turn activates a pro-inflammatory response in the microenvironment, associated with the initiation of irregular and uncontrolled vascular growth. Newly formed tumor vessels appear abnormal and leaky in most of the solid cancers. This structurally aberrant network affects the local blood flow, metabolite exchange, and oxygenation, leading to poor drug delivery, increased tumor growth, and metastatic potential (Carmeliet and Jain, 2011a, Carmeliet and Jain, 2011b, Jain, 2005).

In the AML context, leukemic cells have been shown to modify ECs *ex vivo* via several pathways, mainly mediated by pro-angiogenic factors such as VEGF family members (Kampen et al., 2013, Poulos et al., 2014). Moreover, publicly available datasets as well as several studies reported that VEGF is not the only pro-angiogenic factor produced by AML cells, which may be a major reason why clinical trials incorporating VEGF inhibitors have not been very successful. AML cells also express high levels of hepatocyte growth factor (Aref et al., 2002), fibroblast growth factor (Ribatti et al., 2007), and other factors (Chand et al., 2016, Hatfield et al., 2009, Lee and Kim, 2014). However, no extensive analysis of the AML vascular niche *in vivo* has been reported.

In the present study, we provide a detailed picture of the vasculature in AML. We not only confirm that AML engraftment induces hypoxia and angiogenesis in the BM but also show that it alters the normal vascular architecture and function. ECs involved in the angiogenic process are highly metabolically active and consume a high level of oxygen (Potente and Carmeliet, 2017). As a result, these newly formed abnormal vessels are incapable of supplying nutrients and oxygen (as well as drugs) in a homogeneous way in the BM tissue, likely contributing to maintain the overall hypoxia. At a late stage of disease, after facing an exponential and uncontrolled growth and being exposed to high levels of NO/ROS production, ECs display an increased apoptotic phenotype, likely associated with a toxic process of vascular regression (Lobov et al., 2011).

The crosstalk between leukemic cells and ECs in the BM has not been extensively studied at the molecular level. The available data in the literature rely on the *in vitro* co-culture system, which does not faithfully reproduce the complexity of the *in vivo* environment. Our analysis of the transcriptome of vascular ECs upon human AML engraftment provides important insights into the field. First, it confirms at the molecular level the observed toxic phenotypes. Second, it highlights commonly deregulated genes and pathways, which can represent potential targets for the disruption of the AML-microenvironment crosstalk. Interestingly, the NOX4-NOS3 axis, already reported previously to act in response to stress induced by hypoxic conditions (Craige et al., 2011, Drummond et al., 2000, Mittal et al., 2007, Thomas et al., 2002, Zhang et al., 2008), has a major role in orchestrating the AML-induced vascular leakiness.

Persistent vascular leakiness and increased NO levels in AML xenografts after induction therapy suggest that the abnormal and poorly functional vessels participate to maintain a hypoxic vascular environment during the remission phase, likely contributing to treatment failure. Our hypothesis is strengthened by the analysis of NO in an independent cohort of patients which, although relatively small, faithfully represents the high diversity of the AML genetic landscape. Our analysis argues for NO as a potential prognostic BM marker, as stable/increased levels of NO post-induction therapy seems to be associated with a poor clinical outcome.

Increased vascular leakiness can lead to poor drug delivery and to a microenvironment favoring resistance to therapy and relapse. The participation of vascular leakiness to the pathophysiology of AML can be exerted at different levels. First, increased vascular permeability deregulates the balance of blood supply to the BM and, consequently, drug delivery. This leads to the formation of areas with low perfusion rate, where leukemic cells can hide and escape therapy. Restoring a normal vascular function would allow more homogeneous chemotherapeutic delivery. Second, high vascular permeability (Itkin et al., 2016) and high NO levels (Gur-Cohen et al., 2015) have recently been associated with increased HSC activation and egress from the BM in mice, with a consequent reduction of their repopulating activity. Our data showing increased ROS levels and higher egress of BM-derived HSCs are in line with these findings, further extending them to the human HSPC compartment. Indeed, we show that inhibition of NO results in an enriched number and activity of BM HSPCs. Conversely, the presence of AML engraftment induces the generation of an exogenous overproduction of NO in the BM, which likely affects the HSC motility and activity, bringing a rapid exhaustion of their functionality. In this scenario, inhibition of NO would produce multiple benefits to AML patients. NOS inhibitors are already in clinical trials (Granados-Principal et al., 2015, Ozkor et al., 2015) and specific inhibitors of NOS3 have been developed (Bucci et al., 2000, Gratton et al., 2003). Thus, our preclinical data using both genetic and pharmacologic approaches to inhibit NO in combination with AraC treatment show that targeting vascular permeability would be a successful strategy to normalize the BM vascular functionality, improve therapeutic responses, and preserve normal HSC function.

## STAR★Methods

### Key Resources Table

**Table:** 

REAGENT or RESOURCE	SOURCE	IDENTIFIER
**Antibodies**		

CD45 30-F11 1 in 400 Mouse	eBioscience	Cat: 45-0451-82; RRID: AB_1107002
TER119 Ter-119 1 in 400 Mouse	eBioscience	Cat:12-5921-82; RRID: AB_466042
CD31 390 1 in 400 Mouse	eBioscience	Cat: 25-0311-82; RRID: AB_469616
Endomucin V.7C7 1 in 100 Mouse	Santa Cruz Biotechnology	Cat: sc-65495; RRID: AB_2100037)
Sca-1 D7 1 in 400 Mouse	eBioscience	Cat: 17-5981-83 RRID: AB_469488
Lin - 1 in 400 Mouse	Biolegend	Cat: 133311; RRID: AB_11203535
Kit 2B8 1 in 400 Mouse	BD Bioscience	Cat: 553354; RRID: AB_394805
CD48 HM48-1 1 in 400 Mouse	Biolegend	Cat: 103432; RRID: AB_256163
CD150 TC15-12F12.2 1 in 400 Mouse	Biolegend	Cat: 115914; RRID: AB_439797
IB4 - 100ug/mouse Mouse	Life Technology	Cat: I21411; RRID: AB_2314662
Sca-1/Ly6A/E EPR3355 1 in 100 Mouse	Abcam	Cat: ab109211; RRID: AB_10862573
Ki67 SolA15 2 in 100 Mouse	eBioscience	Cat: 12-5698-82; RRID: AB_11150954
Ki67 20Raj1 2 in 100 Human	eBioscience	Cat: 50-5699-82; RRID: AB_2574237
rAnnexin V - 2 in 100 Mouse/Human	eBioscience	Cat: BMS306; RRID: AB_2687874
CD45 PD7/26 + 2B11 1 in 100 Human	Dako	Cat: M0701; RRID: AB_2314143
CD34 581 1 in 25 Human	BD Bioscience	Cat: 555821; RRID: AB_396150
CD45 2D1 1 in 25 Human	BD Bioscience	Cat: 47-0459-42; RRID: AB_1944368
CD38 HIT2 1 in 25 Human	BD Bioscience	Cat: 555462; RRID: AB_1994368
Ki67 20Raj1 1 in 25 Human	eBioscience	Cat: 50-5699; RRID: AB_2574237
CD45 HI30 20ug/mouse Human	Biolegend	Cat: 304017; RRID: AB_389314
NOS3-pSER1177 C9C3 1 in 100 Mouse/Human	Cell Signaling	Cat: 9570S; RRID: AB_823493
NOS3 33/eNOS 1 in 20 Mouse/Human	BD Bioscience	Cat: 560103; RRID: AB_1645506
NOS2 CXNFT 1 in 100 Mouse/Human	eBioscience	Cat: 17-5920-80; RRID: AB_2573243

**Bacterial and Virus Strains**		

pHAGE2-EF1aFull-MCS-IRES-Tomato	Kind gift of G. Mostoslavsky; modified from J Immunol Methods. 2008;329:31–44	N/A
pTWG-CMV-LUC-eGFP	Modified from pTWG-eGFP (Addgene) and N/A pGL4-50 luciferase reporter (Promega)	N/A

**Biological Samples**		

See Tables 1, S1, and S2	N/A	N/A

**Chemicals, Peptides, and Recombinant Proteins**		

Hypoxyprobe Plus Kit	Natural Pharmacia International	HP2-1000Kit
HypoxiSense 680 Fluorescent Imaging Agent	PerkinElmer	NEV11070
DAF-FM DA solution	Sigma-Aldrich	D2321
CellROX Deep Red Reagent	Thermo Fisher Scientific	C10422
N^G^-Methyl-L-arginine acetate salt (NMMA)	Sigma-Aldrich	M7033
Caveolin-1 scaffolding domain peptide (Cavtratin)	Enzolifesciences	ALX-153-064-M005
S-Nitroso-N-acetyl-DL-penicillamine (SNAP)	Sigma-Aldrich	N3398

**Deposited Data**		

RNA-sequencing data in CD31^+^ BM-derived endothelial cells	GSE88905	http://www.ncbi.nlm.nih.gov/geo/query/acc.cgi?acc=GSE88905

**Experimental Models: Cell Lines**		

HL60	ATCC	Cell Service, The Francis Crick Institute
ML1	ATCC	Cell Service, The Francis Crick Institute
U937	ATCC	Cell Service, The Francis Crick Institute

**Experimental Models: Organisms/Strains**		

NOD-SCID IL2Rg^null^ (NSG)	Jackson Laboratory	005557
NSG-*NESTIN-GFP*	The original strain is a kind gift from Dr G. Enikolopov. Back-crossed to NSG background at the BRF, The Francis Crick Institute (generation 8)	N/A
NSG-*NG2-DSRED*	Original strain from Jackson Laboratory (008241). Back-crossed to NSG background at the BRF, The Francis Crick Institute (generation 8)	N/A
C57BL/6	Jackson Laboratory	000664
C57BL/6 *Nos3*-KO	Jackson Laboratory	002684
NSG- Kit^W41/W41^ (NBSGW)	Jackson Laboratory	026622
C57BL/6-*Rag2*-KO-*GammaC*-KO	BRF, The Francis Crick Institute	N/A

**Oligonucleotides**		

Human VEGFA REV	Sigma-Aldrich	5’-TGGCCTTGGTGAGGTTTGATCC-3’
Human NOS2 FW	Sigma-Aldrich	5’-GGTGCTGTATTTCCTTACGAGGCG-3’
Human NOS2 REV	Sigma-Aldrich	5’-CTTGTTAGGAGGTCAAGTAAAGGGC-3’
Human NOS3 FW	Sigma-Aldrich	5’-CGGCATCACCAGGAAGAAGA-3’
Human NOS3 REV	Sigma-Aldrich	5’-CATGAGCGAGGCGGAGAT-3’
Human GAPDH FW	Sigma-Aldrich	5’-GGGAAGGTGAAGGTCGGAG-3’
Human GAPDH REV	Sigma-Aldrich	5’-GGGTCATTGATGGCAACAA-3’
Murine NOS3 FW	Sigma-Aldrich	5’-CCTTCCGCTACCAGCCAGA-3’
Murine NOS3 REV	Sigma-Aldrich	5’-CAGAGATCTTCACTGCATTGGCTA-3’
Murine NOX4 FW	Sigma-Aldrich	5’-ACTTTTCATTGGGCGTCCTC-3’
Murine NOX4 REV	Sigma-Aldrich	5’-AGAACTGGGTCCACAGCAGA-3’
Murine GAPDH FW	Sigma-Aldrich	5’-AACTTTGGCATTGTGGAAGG-3’
Murine GAPDH REV	Sigma-Aldrich	5’-CACATTGGGGGTAGGAACA-3’

**Software and Algorithms**		

ZEN microscope software	Zeiss	N/A
IMARIS 8.3.1 image analysis software	Bitplane	N/A

### Contact for Reagent and Resource Sharing

Further information and requests for resources and reagents should be directed to the Lead Contact Dominique Bonnet at dominique.bonnet@crick.ac.uk

#### Human Primary Samples

Umbilical Cord Blood (CB) samples were obtained from normal full-term deliveries after signed informed consent. AML samples used for xenografts were obtained after informed consent at St Bartholomew’s Hospital (London, UK). Details are listed in Table 1. BM specimens from 19 adult patients with newly diagnosed, untreated AML were also obtained after informed consent at St Bartholomew’s Hospital (London, UK). Patient characteristics are depicted in Table S1. Additional BM biopsies were available from these same 19 patients following chemotherapy (details in Table S2). To establish controls, BM biopsies were obtained either from healthy donors for bone marrow transplantation or from patients with diffuse large cell lymphoma without BM infiltration (Table S2). The collection and use of all human samples were approved by the East London Research Ethical Committee (REC:06/Q0604/110) and in accordance with the Declaration of Helsinki. Mononuclear cells (MNCs) were isolated by centrifugation using Ficoll-Paque TM PLUS (GE Healthcare Life Sciences, Buckinghamshire, UK). For CB samples, the cells were processed within 24 hours following collection. Cells were enriched for CD34^+^, using an EasySep Human CD34 Positive Selection kit (StemCell Technologies, Vancouver, Canada) according to the manufacturer's instructions, with a purity of 85 to 99%. T cells were depleted from all AML samples using the anti-CD3 mAb OKT-3 (West Lebanon, USA).

#### Mouse Models

All animal experiments were performed under the project license (PPL 70/8904) approved by the Home Office of UK and in accordance to The Francis Crick institute animal ethics committee guidelines. *NESTIN-GFP* mice were a kind gift from Dr G. Enikolopov. NOD-SCID IL2Rg^null^ (NSG), C57BL/6, *NG2-DSRED*, *Nos3*-KO, *Rag2*-KO/*GammaC*-KO and NOD.B6-SCID c-Kit^W41/W41^ (NBSGW) strains were obtained from Jackson Laboratory, Bar Harbor, Maine, USA) and were bred at The Francis Crick Institute Biological Resources Facility in individually vented cages (IVCs) under Specific Pathogen Free (SPF) conditions. NSG-*NESTIN-GFP* and NSG-*NG2-DSRED* mice were obtained by back-crossing the original lines into the NSG background (generation 8 or more).

#### Drug Treatments *In Vivo*

AraC was administrated subcutaneously at the indicated doses (10 mg/kg/day for NSG mice and 60 mg/kg/day for C57BL6 mice) at specific time points, for 7 days, as depicted. To inhibit NOS activity, mice received intraperitoneal injection of NMMA (20 mg/kg/day; Sigma-Aldrich, Gillingham, Dorset, UK) or Cavtratin (2.5 mg/kg/day, Enzo LifeScience), or vehicle, for 2 weeks, 5 days/week. All animal work was in accordance to the Home Office and The Francis Crick guidelines.

#### Intravital Two-Photon Microscopy

For intravital imaging in time-lapse, mice were anesthetized with 2.5% Isoflurane, the head shaved and held in a stereotaxic skull holder. A skin incision revealed the calvaria and methylcellulose (4%, Sigma-Aldrich, Gillingham, Dorset, UK) was applied to prevent the tissue drying. For imaging at a single time point, mice were euthanized by cervical dislocation, the skin was removed, and the intact skull was immerged in PBS for direct examination of the calvarium under the microscope. Images were obtained on a Zeiss 710 NLO laser scanning multiphoton microscope with a 20x 1.0 NA water immersion lens. The microscope was equipped with a MaiTai “High Performance” fully automated 1-box 517 mode-locked Ti:Sapphire laser with DeepSee dispersion compensation (Spectra-Physics), tuned to 890 nm excitation wavelength. To assess the hypoxia with hypoxisense probe, mice received an i.v. injection of 0.4 mg of human normal immunoglobulin twice (48 and 24 hours prior to imaging) (Gammaplex, Bio Products Laboratory, Hertfordshire, UK). The HypoxySense 680 probe (ref: NEV11070, PerkinElmer, Massachusetts, US) was injected 24 hours prior imaging (according to the manufacturer instructions), and on the day of imaging each mouse was intravenously injected with 100 μl of human CD45 AF488 antibody (Biolegend, Cat:304017) 30 minutes prior sacrifice, or with 150 kDa TRITC-dextran in case of NSG-*Nestin-GFP* mice. Mice were imaged as described above. Bone signal (Second Harmonic Generation, SHG) was collected at 380-485nm, hCD45 or Nestin-GFP at 500-550nm, TRITC-dextran at 555-625nm and Hypoxisense at 640-690nm with a 750 nm Infra Red filter by not descanned detectors. To assess vascular perfusion, 100 ug of IB4-AF488 (Life Technology, Cat: I21411) were i.v. injected into mice 30 min prior imaging. 15 μl of Qtracker® 655 Vascular Labels (Thermo Fisher Scientific, Cat: Q21021MP) were injected i.v. 1 min prior imaging. SHG was collected at 380-485nm, IB4-AF488 at 500-550nm, and Qtracker at 640-690nm by not descanned detectors. To assess the hypoxic state of the vasculature, NSG-*Nestin-GFP* mice were used, and TRITC-dextran (150 kDa) was intravenously injected 1 min before imaging to label the vascular tree. SHG was collected at 380-485nm, Nestin-GFP at 500-550nm, TRITC dextran at 555-625nm and Hypoxisense at 640-690nm with a 750 nm Infra Red filter by not descanned detectors. To image the vascular leakiness, we used the strategy detailed in Figure S2E. Briefly, two vessel-pooling agents of different molecular weights were intravenously injected simultaneously: Qtracker® 655 Vascular Labels (Thermo Fisher Scientific, Cat: Q21021MP), high molecular weight and 65-85 kDa TRITRC dextran, medium molecular weight (Sigma-Aldrich, Gillingham, Dorset, UK). Mice were imaged at different time points (Figure 2C) after probes injection, or 10 min after probes administration in all other cases. Calvarium was imaged. SHG was collected at 380-485nm, GFP, 65-85 kDa TRITC dextran at 555-625nm, and 655- Qtracker at 640-690nm by not descanned detectors. Each z stack of images (100-150 μM) was rendered in 3D using Imaris software (Bitplane). At least 9 images representing different areas of bone were taken per group.

#### Images Processing

To measure vessel mean diameter, the ‘filament” algorithm was applied using the signal intensity derived from the NT-Qdot in the BM vasculature (Imaris, Bitplane). To measure the distance to hypoxic areas of BM vessels, a volumetric iso-surface was generated using the signal intensity derived from the Hypoxisense. A map of distances from the Hypoxisense iso-surface was calculated with “Distance Transformation” tool using Imaris XT extension. A volumetric iso-surface to define the vessels was then generated using the intensity derived from the dextran. The distance between each vessel and the closest hypoxiprobe pixel was measured (Bitplane). To assess the vascular leakiness, the signal intensity derived from the Qtracker was used to generate a volumetric iso-surface of the BM vasculature, using the standard surface algoritm of IMARIS. “Background Subtraction” (5-10% based on local contrast) was performed with manual threshold. The iso-surface was then used to segment the signal intensity (SUM) derived from the dextran in “IN” and “OUT” the vasculature, using the “Mask Settings” function. The ratio between these two intensities (OUT/IN) represents the leakiness of the vasculature at a given time point (Imaris, Bitplane). To measure the distance of AML cells to vessels, a volumetric iso-surface was generated using the signal intensity derived from the dextran. A map of distances from the Hypoxisense iso-surface was calculated with “Distance Transformation” tool using Imaris XT extension. The spot function was used to identify hCD45^+^ cells. The distance between each spot and the closest vascular pixel was measured (Bitplane). To quantify the hypoxia, in each z-stack the hypoxisense signal intensity and the total volume of the z-stack (voxel) were calculated. The hypoxia was expressed as signal intensity/voxel (Imaris, Bitplane).

#### Bioluminescence Imaging

Before imaging, each mouse was given an intraperitoneal injection of 125 mg/kg luciferin (Caliper Life Science). General anesthesia was induced with 2% isoflurane and the mouse was placed in a light-tight heated chamber. After acquiring bright-field images of each mouse, anterior and posterior luminescent images were then successively acquired with 1- to 5-min exposure time. Optical images were displayed and analyzed with the IVIS Living Image software (Caliper Life Science). Regions of interest were manually drawn around the bodies of the mice to assess the intensity of the bioluminescence signal for each mouse. Optical signal was expressed as photon count.

#### Cell Lines

293 HEK were grown in DMEM; HL60, ML1 and U937 were grown in RPMI1640, MS5 in IMDM. All cell lines were tested for mycoplasma prior to commencing experiments. All cell lines came originally ATCC (distributor LGC standards, UK) and were grown by our cell service at the Institute. Before use these lines, they were authenticated using the Short Tandem Repeat (SRF) profiling. All media were supplemented with 10% FBS and 1x Penicillin-Streptomycin and all reagents were from Gibco®-Life Technologies (Paisley, UK).

#### Transduction and Culture of Human Primary Samples

T-cell depleted hAML MNCs were pre-stimulated in StemSpam SFEM (StemCell Technologies, Vancouver, Canada) supplemented with 25 ng/ml G-CSF or 100ng/ml SCF, 100ng/ml Flt3-L, 60ng/ml IL-3 and 10ng/ml TPO (PrepoTech, London, UK), respectively for 4 to 6 hours. CD34-enriched HSPCs were pre-stimulated in StemSpam SFEM (StemCell Technologies, Vancouver, Canada) supplemented with 25 ng/ml G-CSF, 150ng/ml SCF, 150ng/ml Flt3-L, 10ng/ml IL-6 and 20ng/ml TPO (PrepoTech, London, UK) for 4 to 6 hours. Lentiviral supernatant (GFP-Luciferase vector for human AML MNCs and tomato vector (Pan et al., 2008) for HSPCs) was added at a multiplicity of infection (MOI) of 30 in the presence of 5μg/mL polybrene for 12 hours. The cells were then washed and expanded in culture. The efficiency of transduction was analyzed after 4 days by GFP expression. Primary hAML cells were co-cultured on a layer of primary human MSC for one week. Transduced AML cells were co-cultured on MS-5 cells for 4 days before sorting on the human CD45 and GFP fraction and injected into mice. Cells were grown in Myelocult H5100 medium (StemCell Technologies, Vancouver) supplemented with 20ng/ml IL-3, 20ng/ml G-CSF and 20ng/ml TPO (PeproTech, London, UK). Transduced HSPC cells were grown in StemSpam SFEM supplementer with 150 ng/ml SCF, 150 ng/ml Flt-3 and 20 ng/ml TPO for 4 days before sorting on the human CD45 and tomato fraction and injected into mice.

#### Transduction and Culture of Murine Primary Samples

Primary murine AML carrying the MLL-AF9 or MLL-ENL translocations were generated as reported (Horton et al., 2009). Leukemic cells retrieved from BM or spleen of primary leukemic mice (MLL-AF9 #1, MLL-ENL #1 and #2) were cultured in RPMI supplemented with 10% FCS, 10 ng/ml IL3, 10 ng/ml IL6, 100 ng/ml SCF (Sigma-Aldrich, Gillingham, Dorset, UK). Cells were infected by spinoculation with a lentivirus expressing GFP-Luciferase (MLL-AF9 #1 and MLL-ENL #1) or tomato (MLL-ENL #2) (centrifugation at 700*g*, 25°C, 45 minutes) in the presence of 5μg/mL polybrene (Sigma-Aldrich, Gillingham, Dorset, UK), as previously described (Horton et al., 2005).

#### AML and HSPCs Transplantation Assay *In Vivo*

##### Murine AML

Murine AML cells from culture were transplanted into sub-lethally irradiated C57BL6 mice. Secondary AML cells retrieved from these animals were transplanted into unconditioned WT or *Nos3*-KO mice at the indicated concentrations.

##### Murine Normal HSC

For HSC *in vivo* functionality assays, murine CD45.1 cells retrieved from NSG mice were isolated using an EasySep murine CD45 Positive Selection kit (StemCell Technologies, Vancouver, Canada) and 250000 cells were transplanted i.v. into sub-lethally irradiated *Rag2*-KO/*GammaC*-KO secondary recipients.

##### Xenografts

For xenografts assays, human AML cell lines, AML patient-derived samples and HSPC were transplanted into 8- to 12-week old unconditioned NSG or NSG*-NESTIN-GFP* and NSG*-NG2-DSRED* mice by i.v. injection, as indicated. For competitive transplantation experiments, 5,000 tomato^+^ HSPCs were injected together with 100000 untransduced AML6 cells i.v. into unconditioned NBSGW mice (Jackson Laboratory, BarHarbor, Maine, USA). BM engraftment was assessed by BM aspirate or bioluminescence, when indicated.

#### Bone Marrow Cell Isolation

At the end of each experiment, animals were euthanized by cervical dislocation and the 6 rear bones collected in cold PBS. To retrieve BM endothelial and mesenchymal cells, bones were dissected in small pieces of 2-3 mm diameter and incubated 30 min at 37 degrees in a HBSS digestion solution containing 2% FBS, DNase I (10 μg/ml), Collagenase (1 mg/ml), Dispase II (5 mg/ml) and Heparin (20 U/ml), all from Sigma-Aldrich, Gillingham, Dorset, UK. Bone pieces were next crashed with mortar and pestle in the same digestion solution and incubated at 37 degrees for 30 min. Cell suspension was then homogenized with a micropipette and filtered with a 100 micron cell strainer.

#### HO Diffusion

Mice were administrated with two injections of 0.4 mg HO (Sigma-Aldrich, Gillingham, Dorset, UK) at 5 minutes interval, and euthanized 5 minutes later. After cervical dislocation, bones were quickly dissected and immediately immersed in ice-cold PBS containing inhibitors of the APC-efflux pump (Reserpine, Verapamil; Sigma-Aldrich, Gillingham, Dorset, UK) to avoid HO extrusion. Cells were recovered and processed as described above and stained with specific surface antibodies. HO uptake was calculated by dividing the geometric mean fluorescence intensity of the HO blue channel by the same analysis carried out on the same population of cells from the same organ but from an animal not injected with HO (negative control). This corresponds to the specific fluorescence intensity and reflects the actual uptake of HO by the population of interest.

#### Hypoxiprobe Assessment

Pimonidazole (PIM; Hypoxyprobe) was purchased from Natural Pharmacia International, Inc. Mice were i.v. injected with 125 mg/kg PIM or saline solution and euthanized after 2 hours. For flow cytometry analysis, bones were rapidly harvested and kept in PBS at 4°C. Cells were recovered and processed as described above, and stained with specific surface antibodies. Next, they were permeabilized (BD Cytofix/Cytoperm kit) and stained with specific FITC-MAb1, following manufacturer instructions. Cells from PIM non-injected animals but stained with the same concentration of relevant antibody after permeabilization were used as a negative control to calculate the specific geometric mean fluorescence intensity as previously reported (Lassailly et al., 2013). For IHC analysis, samples were processed as described below, and stained with primary FITC-MAb1 and secondary anti-FITC-HRP following manufacturer instructions.

#### Flow Cytometry Analysis and Cell Sorting

Flow cytometry analysis was performed using a Fortessa flow cytometer (BD Biosciences, Oxford, UK). AML transduced cells were identified based on their GFP expression. Dead cells and debris were excluded from the analysis using 4,6, diamidino-2-phenylindole (DAPI) staining. Murine HSC (SLAM) were defined by the expression of these surface markers: CD45^+^LIN^-^Kit^+^CD48^-^CD150^+^, with the exclusion of Tomato+ murine AML cells or hCD45+ human AML cells. DAF-FM probe was used to measure intracellular NO, using manufacturer instructions (D2321, Sigma-Aldrich, Gillingham, Dorset, UK). In particular, bone marrow cells were incubated for 30 min at 37 degrees with the probe at 5 uM, and extensively washed afterwards before analysis. CellROX Deep Red Reagent was used to measure cellular ROS, following manufacturer instructions (C10422 – ThermoFisher Scientific). For intracellular staining, cells were fixed and permeabilized after surface epitope staining using BD Cytofix/Cytoperm kit, following manufacturer instruction (BD Bioscience, Oxford, UK). Cell sorting was performed using a FACS Aria SORP (BD Biosciences, Oxford, UK). Human AML cell lines and primary patient-derived AML cells were sorted according to the expression of GFP and hCD45. Murine endothelial and mesenchymal cells were analyzed and sorted based on the absence of expression of CD45 and Ter119 makers, and the positive expression of CD31 and Nestin-GFP, respectively. Flow cytometry plots displayed in this manuscript represent one set of data points from at least 3 replicates. Antibodies informations are included in the Key Resources Table.

#### Drug Treatments *In Vitro*

Human ML1 cells were seeded alone or on a confluent layer of MS-5 cells for 24 hours before starting the treatments. NMMA was used at a concentration of 5 mM for 48 hours before assessing apoptosis. Primary AML-derived samples were seeded on a confluent layer of MS-5 cells for 4 days before starting the treatment. Drugs were added to the culture (NMMA at 5 mM and SNAP at 10 μm; Sigma-Aldrich, Gillingham, Dorset, UK) for 5 days before assessing apoptosis and cell cycle.

#### Reverse Transcriptase and Real Time Quantitative PCR (RT-qPCR)

Total RNA from sorted cell samples was isolated using the RNeasy Mini Kit (Qiagen, Crawley, UK) according to the manufacturer’s instructions. mRNA was reverse transcribed by Superscript III Reverse Transcriptase (Invitrogen) with an oligoDT primer (Sigma-Aldrich, Gillingham, Dorset, UK). RT-qPCR was performed with an ABI 7500 FAST Thermal Cycler (Applied Biosystems) using SYBR Green dye (Applied Biosystems). RNA was quantified with the Comparative CT Method and GAPDH was used as a housekeeping gene. The CT values used were the result of triplicates. The primers used are described in the Key Resources Table.

#### Histological Processing and Immunostaining

Murine samples. Harvested humerus samples were fixed o/n in 10% neutral buffered formalin and then decalcified with 17% EDTA (Osteosoft, Millipore) during 7 days. Samples were processed, paraffin embedded and sectioned (5 μm) for histological studies. Hematoxylin/eosin was performed first to assess quality of the sections of BM samples and visualize vessel integrity. For immunofluorescence (IF) studies heat antigen retrieval was performed in all cases. Primary antibodies are listed in the key resources table. Goat anti-mouse/rabbit/rat secondaries antibodies coupled to AF-488, AF555 and AF647 were used (all from Invitrogen). DAPI was included in the mounting media to label the nuclei. Images were obtained with a Zeiss LSM710 upright confocal microscope. At least 3 images were taken per sample group. To quantify micro-vascular density (MVD), the number of vessel sprouts/mm2 was counted (Bitplane), and data are show as fold over ctrl.

#### Sample Preparation for RNA-Sequencing

The quality and concentration of total RNA were determined on Agilent 2100 Bioanalyzer using the Eukaryote Total RNA Pico Assay. Most of the total RNA has average RIN number 5-8, with a concentration at least 4 pg/μl. Some of the samples were concentrated on a speedvac without heat to obtain a final volume of 5 μl. 3.5 ng of Total RNA in 5 μl volume was used to generate cDNA synthesis with Nugen Ovation® RNA-Seq System V2 kit (part No. 7102). The resulting SPIA-cDNA were normalized to 100 ng in 15 μl based on Qubit DNA HS assay. Fragmentation was done using 8 microTUBE-15 AFA Beads Strip V2 (PN 520159) on Covaris E-series at setting 20%DF, 18W, 200 burst, 20C tempt, 120s treatment time and no Intensifier. Only 10-30 ng in 10 μl of fragmented cDNA was used starting from the End Repair step of the Ovation® Ultralow Library Systems V2 (part No. 0344NB) protocol, with 10 cycles PCR. The RNAseq libraries were quality checked on Qubit DNA HS assay, Bioanalyser and Illumina Ecoreal QPCR followed by Illumina PE51 sequencing on Hiseq 2500 V3 reagents.

#### RNA-Sequencing Analysis Methods

FastQ files were generated using CASAVA BCL to FastQ (version 2.16). Sequencing yield was typically ∼25 million strand-specific paired-end reads. The RSEM package (version 1.2.29) (Li and Dewey, 2011) in conjunction with the STAR alignment algorithm (version 2.5.1b) (Dobin et al., 2013) was used for the mapping and subsequent gene-level counting of the sequenced reads with respect to mm10 Ensembl genes downloaded from the UCSC Table Browser 15 on 14th April 2016 (Karolchik et al., 2004). The “--forward-prob” parameter was set to “0” and all other parameters were kept as default. Differential expression analysis was performed with the DESeq2 package (version 1.10.1) (Love et al., 2014) within the R programming environment (version 3.2.3) (R Development Core Team, 2015). Genes with FDR <0.05 were judged to be differentially expressed. Genes from each given pairwise comparison were ranked using the Wald statistic. GSEA pre-ranked analysis was performed with respect to MSigDB (version 5.1) (Subramanian et al., 2005) genesets C2 canonical pathways and C5 GO biological process. All parameters were kept as default except for enrichment statistic (weighted), min size (5) and max size (50000).

#### Statistical Analysis

Statistics were performed with Prism (GraphPad). Unpaired Student's t-test was used to compare two groups, except in Figure 5E and S3B and S5B, where paired Student’s t-test was used. Log-rank test was used for survival curves. Details of each analysis are in figure legends. For probabilities distribution in our cohort of patients, we have considered the joint probability distribution of two Boolean variables: NO normalization and response to therapy. We applied Bayesian inference to estimate the probabilities of each of the four possible combinations of these variables from the cohort of patients. These probabilities are related to the occurrence of each combination of NO normalization and response to therapy, according to a multinomial distribution parameterized by the four probabilities. We estimated these parameters from their posterior distributions obtained by updating a uniform prior using the data available. The posterior distributions for the four probabilities have been obtained by generating a sample of the parameters of the multinomial (N=1 10^6^), weighted according to importance sampling. The generated sample was then used to calculate relevant conditional probabilities and their corresponding posterior distributions, which allow the calculation of maximum a posteriori (MAP) estimates and 95% credible intervals (CI95). The analysis was performed using custom-made software written in R (R Development Core Team, 2015). A similar analysis was performed including only intermediate risk group patients.

### Data and Software Availability

#### Data Resources

The accession number for the transcriptome sequencing data generated in this study are deposited on Geo bank (reference number: GSE88905).

## Author Contributions

D.P. designed, conducted, and performed the experiments and wrote the manuscript. A.d.T., A.A., K.R.-P., K.F., F.L., L.A.M., and B.M. performed some experiments. L.B. prepared the libraries and P.C. performed the statistical analysis of RNA-seq data. G.D. performed statistical analysis on patients' cohorts. J.G. provided patient samples and clinical information. D.B. designed experiments and wrote the manuscript.

## Supplemental Information

Document S1. Figures S1–S7 and Tables S1 and S2

Document S2. Article plus Supplemental Information
