# Supplementary material for: Increased Vascular Permeability in the Bone Marrow Microenvironment Contributes to Disease Progression and Drug Response in Acute Myeloid Leukemia
Source: Cancer Cell. 2017 Sep 11;32(3):324–341.e6. doi: 10.1016/j.ccell.2017.08.001 (PMC5598545; doi:10.1016/j.ccell.2017.08.001)
Supplement: Document S1. Figures S1–S7 and Tables S1 and S2 [file mmc1.pdf]

Cancer Cell, Volume *32*

Supplemental Information

Increased Vascular Permeability in the Bone Marrow Microenvironment Contributes to Disease Progression and Drug Response in Acute Myeloid Leukemia

Diana Passaro, Alessandro Di Tullio, Ander Abarrategi, Kevin Rouault-Pierre, Katie Foster, Linda Ariza-McNaughton, Beatriz Montaner, Probir Chakravarty, Leena Bhaw, Giovanni Diana, François Lassailly, John Gribben, and Dominique Bonnet

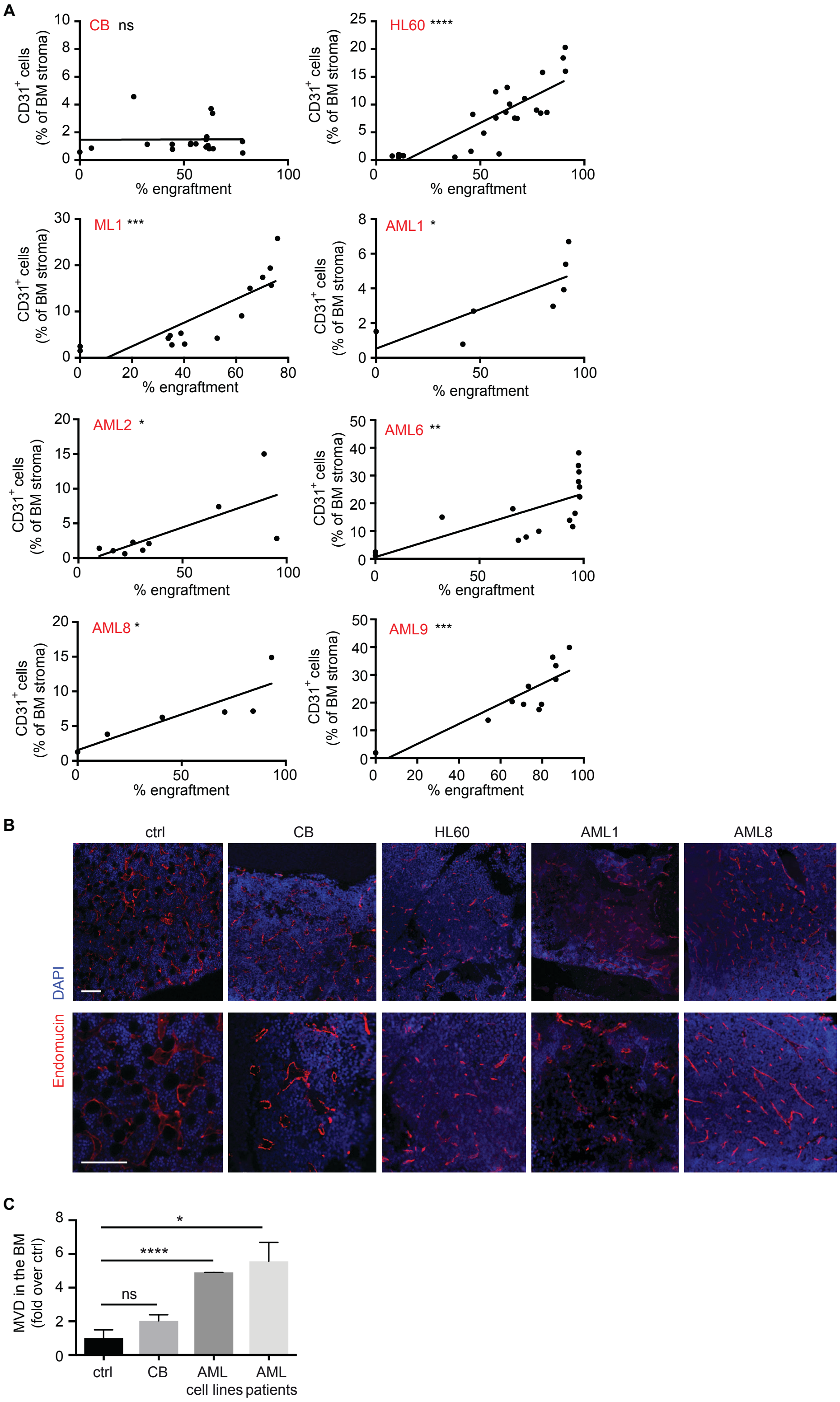

A
CB ns
HL60 ****
ML1 ***
AML1 *
AML2 *
AML6 **
AML8 *
AML9 ***
CD31+ cells (% of BM stroma)
% engraftment
B
ctrl
CB
HL60
AML1
AML8
DAPI
Endomucin
C
MVD in the BM (fold over ctrl)
ns
****
*
ctrl
CB
AML cell lines
AML patients

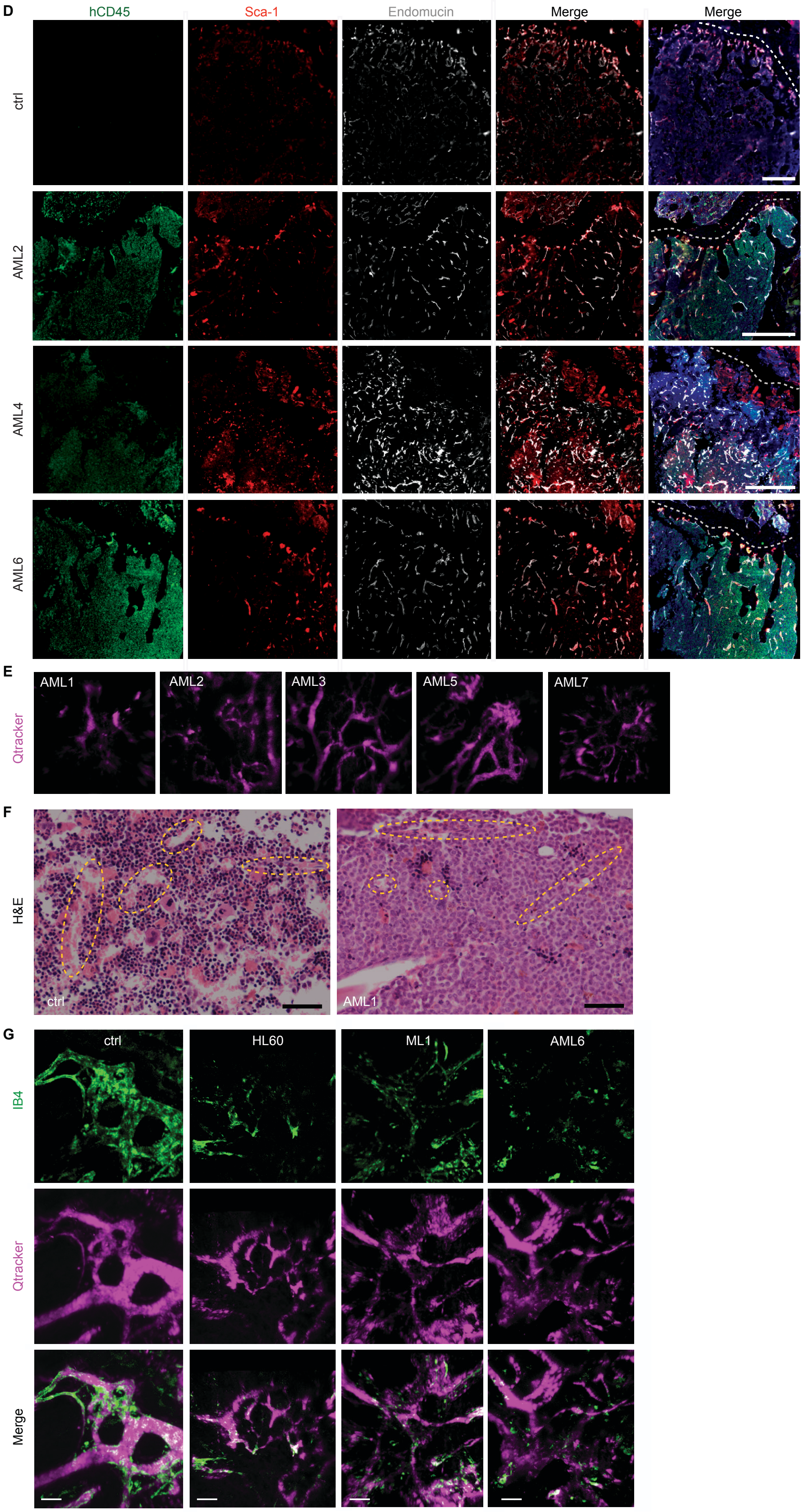
D
hCD45
Sca-1
Endomucin
Merge
Merge
ctrl
AML2
AML4
AML6
E
AML1
AML2
AML3
AML5
AML7
Qtracker
F
H&E
ctrl
AML1
G
ctrl
HL60
ML1
AML6
IB4
Qtracker
Merge

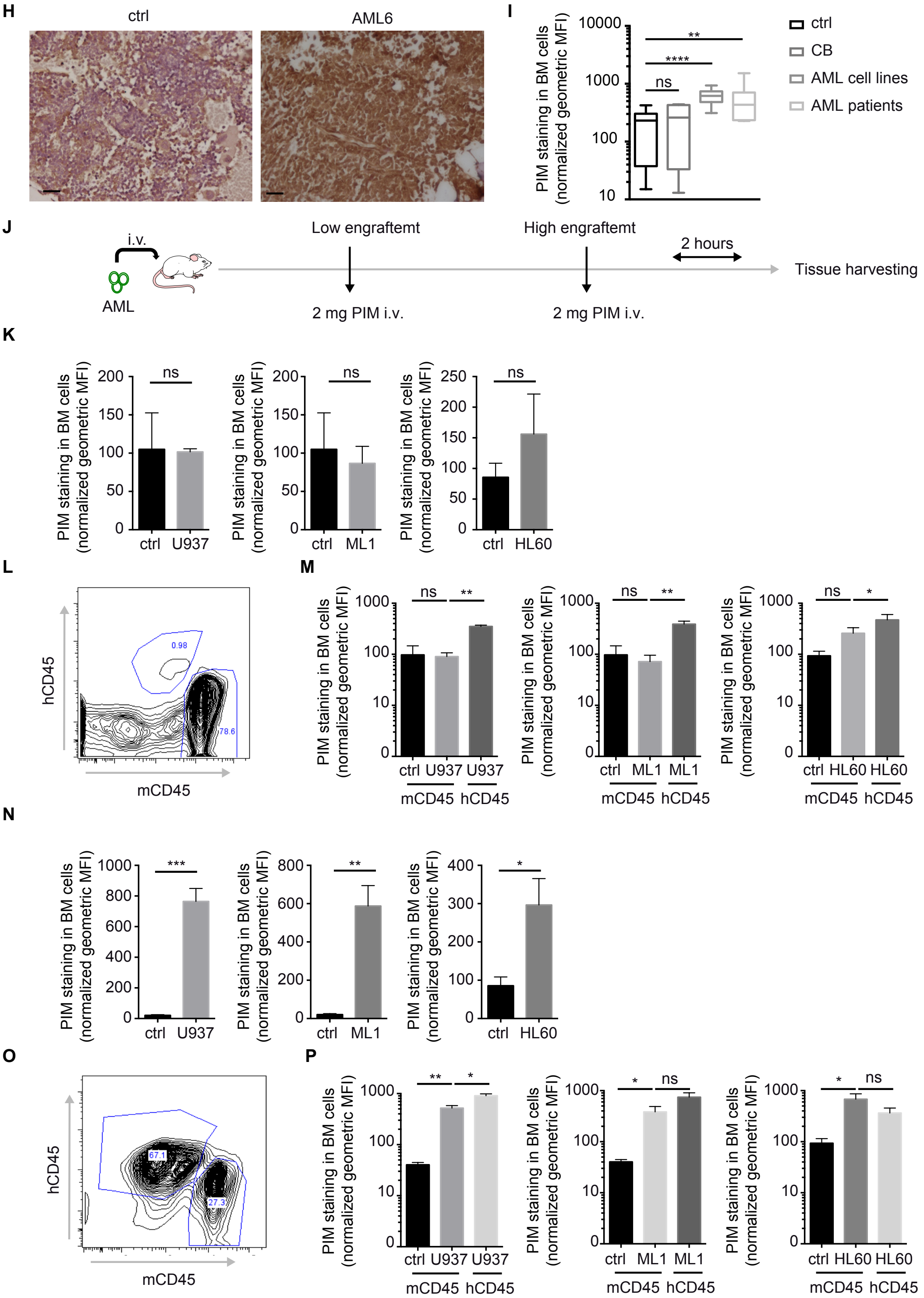

H
ctrl
AML6
I
PIM staining in BM cells (normalized geometric MFI)
10000
1000
100
10
**
****
ns
ctrl
CB
AML cell lines
AML patients
J
i.v.
AML
Low engraftemt
High engraftemt
2 hours
Tissue harvesting
2 mg PIM i.v.
2 mg PIM i.v.
K
PIM staining in BM cells (normalized geometric MFI)
ns
ctrl U937
ctrl ML1
ctrl HL60
L
hCD45
mCD45
0.98
78.6
M
PIM staining in BM cells (normalized geometric MFI)
ns **
ns **
ns *
ctrl U937 U937
ctrl ML1 ML1
ctrl HL60 HL60
mCD45 hCD45
N
PIM staining in BM cells (normalized geometric MFI)
***
**
*
ctrl U937
ctrl ML1
ctrl HL60
O
hCD45
mCD45
67.1
27.3
P
PIM staining in BM cells (normalized geometric MFI)
** *
* ns
* ns
ctrl U937 U937
ctrl ML1 ML1
ctrl HL60 HL60
mCD45 hCD45

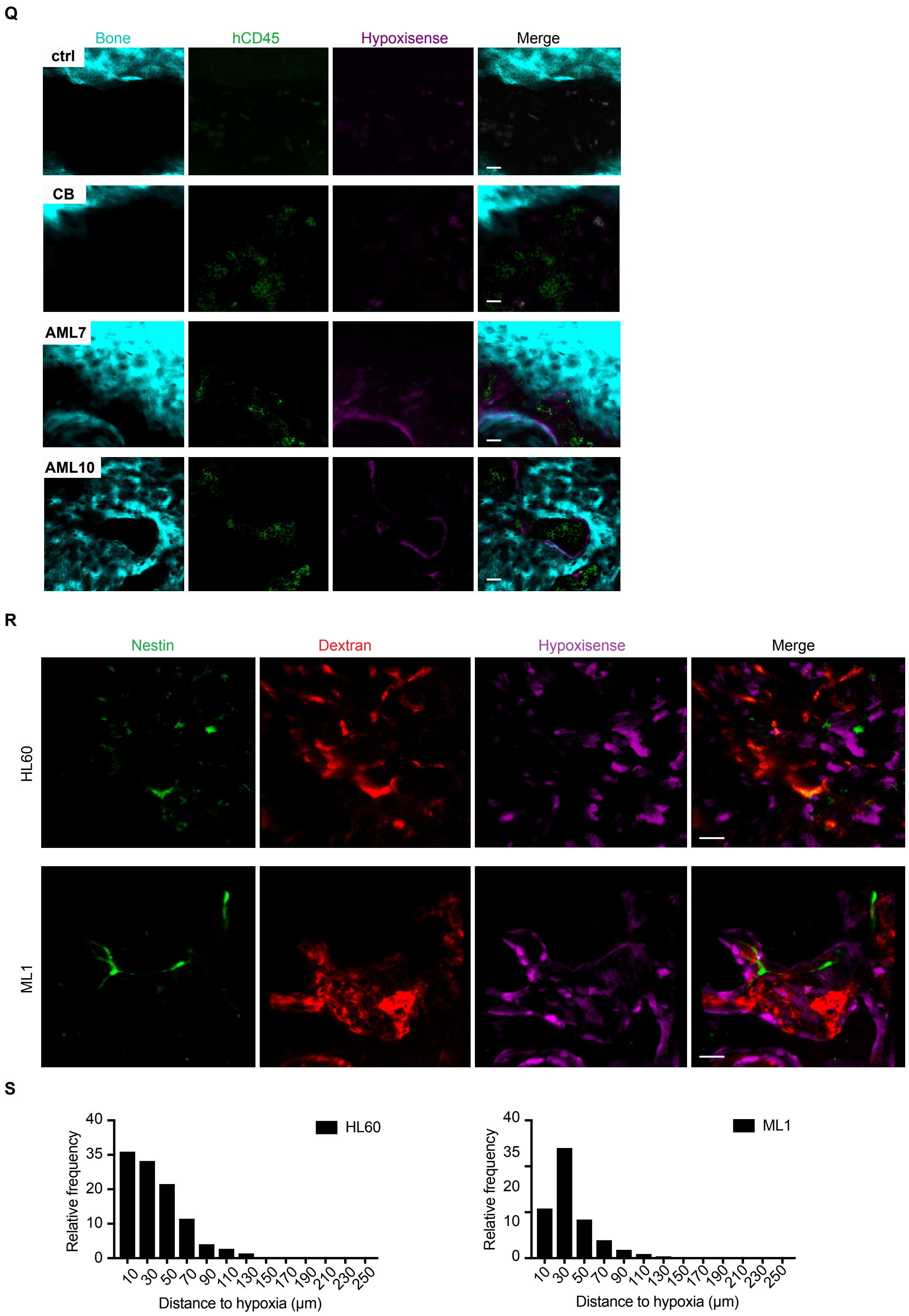

Figure S1. Related to Figure 1. AML derived alteration of the BM vascular architecture and function. (A) Quantification of $CD31^+$ endothelial cells in the BM (shown as % of $CD45^-Ter119^-$ BM cells) in correlation with the human normal or leukemic engraftment, as depicted. Linear regression is shown. **(B)** Immunofluorescence images of fixed long bones showing endomucin (red)

vasculature of non-transplanted mice or mice transplanted with CB derived HSPCs (CB), human HL60 cells or AML patient-derived cells, as depicted. Nuclei are stained with DAPI (blue) Bars represent 200 μm. **(C)** Microvascular density (MVD) quantified via endomucin staining in the BM of non-transplanted mice (ctrl) and mice transplanted with CB-derived HSPCs (CB) or human AML patients' derived samples. Ctrl n=5; CB n=11; AML cell lines n=7; AML patients (AML1, 6) n=9. Data are shown as mean ± s.e.m. **(D)** Immunofluorescence images of fixed long bones of non-transplanted mice or mice transplanted with AML patient-derived samples, showing endomucin (white) and Sca-1 (red) vasculature in association with leukemic cells (hCD45, green). Nuclei are stained with DAPI (blue). Bars represent 500 μm. **(E)** Representative 3D reconstruction of BM vasculature of the calvarium of mice transplanted with AML patient-derived samples, as depicted, imaged via 2P microscopy 1 min after injection of 655-conjugated NT-Qtracker as vessel pooling agent. Data are representative of 5 independent AML derived PDX done in triplicates. Bars represent 70 μm. **(F)** H&E staining of long bones of non-transplanted mice (ctrl) or mice transplanted with AML1 patient-derived cells. Yellow circles indicate vessel lumen. Bars represent 50 μm. **(G)** Representative 3D reconstruction of BM vasculature of the calvarium of non-transplanted mice (ctrl) or AML xenografts, as depicted, imaged via 2P microscopy. IB4 (green) labeling of endothelial cells represents vascular perfusion, and Qtracker (purple) labels the vascular tree. Data are representative of at least 9 replicates per group. Bars represent 70 μm. **(H)** Representative IHCs showing the hypoxic staining via hypoxiprobe of long bones of non-transplanted mice (ctrl) and mice transplanted with AML6 patient-derived cells, as depicted. Bars represent 50 μm. **(I)** Hypoxiprobe staining of BM cells isolated from non-transplanted mice (ctrl) and mice transplanted with CB-derived HSPCs (CB) or AML cell lines or patient-derived samples, as depicted. Ctrl n=14; CB n=6; AML cell lines n=9; AML patients n=11. Data are shown as Whiskers min to max plots, the line inside the box representing the mean, the top and the bottom line representing the 75% and 25% percentiles, respectively, and the lines above and below the box representing the S.D. **(J)** Schematic of the experiment: Mice engrafted with human AML cells were assessed for BM engraftment. At specific time points (low and high engraftment), 2 mg of hypoxiprobe were injected intravenously 2-hours prior sacrifice. Bones were retrieved and hypoxiprobe intracellular adducts were measured either via IHC or intracellular flow cytometry staining. **(K)** Low engraftment**:** hypoxiprobe staining in BM cells of non-transplanted mice or mice transplanted with human AML cell lines, as depicted; n=3 per condition. Data are shown as mean ± s.e.m. **(L)** Flow cytometry gating strategy. **(M)** Hypoxiprobe staining in different populations of BM cells in mice sacrificed at low leukemic engraftment; n=3 per condition. Data are shown as mean ± s.e.m. **(N)** High engraftment: Hypoxiprobe staining in BM cells of non-transplanted mice or mice transplanted with human AML cell lines, as depicted; n=3 per condition. Data are shown as mean ± s.e.m. **(O)** Flow cytometry gating strategy. **(P)** Hypoxiprobe staining in different populations of BM cells in mice sacrificed at high leukemic engraftment; n=3 per condition. Data are shown as mean ± s.e.m. **(Q)** Representative z-stacks of BM hypoxia imaged via intravital microscopy using the hypoxisense probe together with bone (SHG) and $hCD45^+$ cells in non-transplanted mice or mice transplanted with CB-derived HSPCs (CB) or AML patient-derived samples, as

depicted. Bars represent 30 µm. **(R)** Representative 3D reconstruction of BM hypoxia imaged via intravital microscopy using the hypoxisense probe together with vasculature (Dextran) and Nestin$^+$ cells in mice transplanted with AML cell lines, as depicted. Bars represent 50 µm. **(S)** Distribution and relative frequency of vessel distances to hypoxic areas in the BM of non-transplanted mice (ctrl) or mice transplanted with AML cell lines, as depicted. P values: ns: non-significant; * $p<0.05$; ** $p<0.01$; *** $p<0.001$.

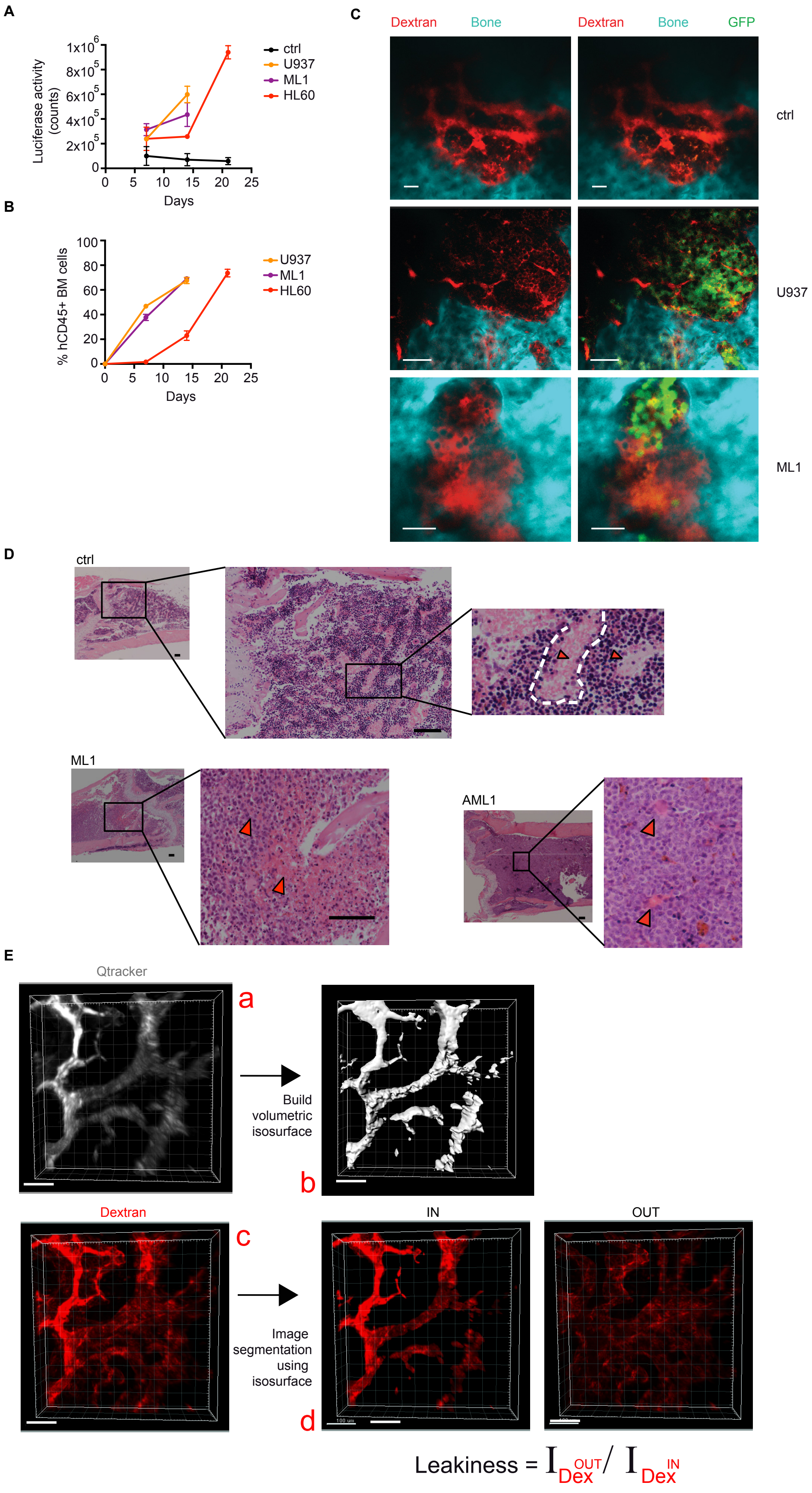
A
Luciferase activity (counts)
1x10^6
8x10^5
6x10^5
4x10^5
2x10^5
0
0 5 10 15 20 25
Days
ctrl
U937
ML1
HL60
B
% hCD45+ BM cells
100
80
60
40
20
0
0 5 10 15 20 25
Days
U937
ML1
HL60
C
Dextran Bone
Dextran Bone GFP
ctrl
U937
ML1
D
ctrl
ML1
AML1
E
Qtracker
a
Build volumetric isosurface
b
Dextran
c
Image segmentation using isosurface
d
IN
OUT
Leakiness = I Dex OUT / I Dex IN

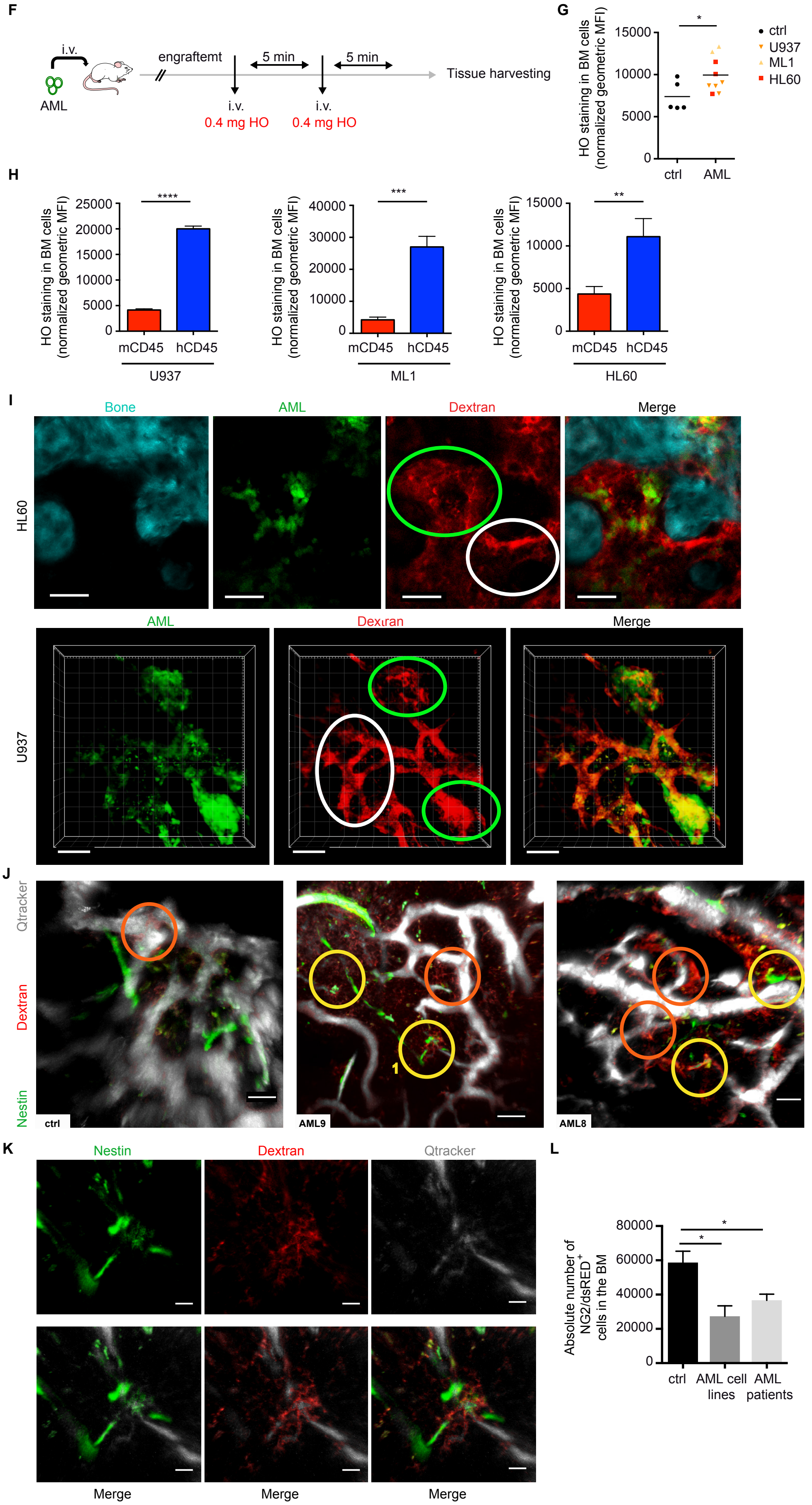
F
i.v.
AML
engraftemt
5 min
5 min
i.v.
0.4 mg HO
i.v.
0.4 mg HO
Tissue harvesting
G
HO staining in BM cells (normalized geometric MFI)
15000
10000
5000
0
ctrl
AML
ctrl
U937
ML1
HL60
H
HO staining in BM cells (normalized geometric MFI)
mCD45
hCD45
U937
ML1
HL60
I
Bone
AML
Dextran
Merge
HL60
U937
J
Qtracker
Dextran
Nestin
ctrl
AML9
AML8
K
Nestin
Dextran
Qtracker
Merge
L
Absolute number of NG2/dsRED+ cells in the BM
80000
60000
40000
20000
0
ctrl
AML cell lines
AML patients

Figure S2. Related to Figure 2. AML engraftment increases vascular permeability in the BM. (A) Kinetics of BM engraftment shown as luciferase activity in mice (n=3 per condition) transplanted with human AML cell lines. Data are shown as mean ± s.e.m. **(B)** Kinetics of BM engraftment shown as percentage of $hCD45^+$ cells in the BM of mice (n=3 per condition) transplanted with human AML cell lines. Data are shown as mean ± s.e.m. **(C)** Representative z-stack of the TRITC-Dextran labeled BM vasculature in the calvarium of non-transplanted control or from mice transplanted with GFP-U937 and GFP-ML1 cells. Data are representative of triplicates. Bars represent 30, 70, 50 µm for ctrl, U937 and ML1, respectively. **(D)** H&E staining of the long bones of non-transplanted mice (ctrl) or mice transplanted with AML xenografts, as depicted. Red arrows point to erythrocytes. Bars represent 50 µm**. (E)** The quantification strategy of bone marrow vascular permeability is shown. The volumetric signal derived from the 655-conjugated Qtracker (a) is used to create an isosurface of the vascular tree in the BM (b). This isosurface is used to segment the volumetric signal derived from the Dextran (c) inside and outside the vasculature. The leakiness is shown as a ratio OUT/IN (d). The formula used to measure the leakiness is shown (I = intensity signal). Bars represent 100 µm. **(F)** Schematic of the experiment: HL60, ML1 and U937 human AML cells were injected intravenously into NSG mice. Once engraftment confirmed, two consecutive intravenous injections of 0.4 mg of Hoechst (HO) were performed at 5 mns interval. Mice were sacrificed 5 mns after the last injection, and BM cells retrieved in cold PBS containing inhibitors of HO expulsion. **(G)** HO signal was analyzed by flow cytometry in BM cells of non-transplanted mice or mice engrafted with AML cell lines, as depicted (normalized geometric MFI). Bars represent mean. **(H)** HO signal in murine $CD45^+$ and human $CD45^+$ cells in the BM of mice detailed in B; n=3 per condition. Data are shown as mean ± s.e.m. **(I)** Representative Z-stacks (HL60) and 3D reconstruction (U937) of the BM vasculature in the calvarium of mice after injection of TRITC-Dextran vessel pooling agent. SHG: Second harmonic signal generated by the bone. White ellipses indicate non-engrafted areas; green ellipses indicate AML engrafted areas. Bars represent 50 µm (HL60) and 100 µm (U937). **(J)** Representative 3D reconstruction of the BM vasculature in the calvarium of non-transplanted mice or AML xenografts, as depicted, after injection of Qtracker and Dextran. Nestin-GFP signal (green) labels arteriolar vsculature. Orange and yellow circles indicate $Nestin^-$ and $Nestin^+$, respectively, leaky vessels. Bars represent 50 µm. **(K)** Higher magnification of area n1 highlighted in (J). Bars represent 10 µm. **(L)** Absolute number of $NG2\text{-}DSRED^+$ cells in the BM of non-transplanted mice (ctrl, n=3) or mice engrafted with human AML cell lines (HL60 n=2; ML1 n=3) or patient-derived samples (AML6, n=6; AML9, n=5). Bars represent mean ± s.e.m. p values: *p<0.05; *** p< 0.001; **** p<0.0001.

Table S1. Related to Figure 3. BM biopsies obtained from AML patients and donors with normal BM

PATIENT ID	CYTOGENETICS	AGE	GENDER	NPM/FLT3	CLINICAL INFORMATIONS
AML11	T(4;17;15;7)	34	FEMALE	WT/MUT	n.d.
AML12	TRISOMY 8	44	MALE	WT/WT	M1
AML13	T(15;17)	22	FEMALE	WT/WT	M3
AML14	n.d.	46	FEMALE	WT/WT	M1
AML15	T(15;17) +	39	FEMALE	WT/WT	M3
AML16	PLUS X	30	FEMALE	WT/WT	M0
AML17	NORMAL	65	FEMALE	WT/WT	2ND AML
AML18	NORMAL	55	FEMALE	WT/WT	M0
AML19	NORMAL	37	FEMALE	WT/MUT	M5
AML20	NORMAL	62	MALE	WT/WT	2ND AML
AML21	NORMAL	43	MALE	WT/WT	TRIPHENOTYPIC: B,T, M
AML22	NORMAL	49	FEMALE	WT/MUT	M1
AML23	NORMAL	54	MALE	WT/WT	M2
AML24	NORMAL	63	MALE	WT/WT	M5
AML25	NORMAL	64	MALE	MUT/MUT	M0
AML26	T(15;17) +	41	FEMALE	WT/WT	M3
AML27	NORMAL	69	MALE	WT/WT	M2
AML28	INS(10;11)MLL(11Q23)REARRANGED	31	MALE	WT/MUT	M1
AML29	TRISOMY 8	68	MALE	WT/MUT	M2
HC1	-	34	MALE	-	DLBC - Stage IE
HC2	-	45	MALE	-	DLBC - Stage IE - chest wall
HC3	-	86	FEMALE		DLBC - Stage II
HC4	-	69	MALE	-	DLBC - Stage IIE - THYROID
HC5	-	78	MALE	-	DLBC - Stage IE - TESTES
HC6	-	40	FEMALE	-	BMT donor - B-thalassaemia trait
HC7	-	27	FEMALE	-	BMT donor - B-thalassaemia trait
HC8	-	37	FEMALE	-	BMT donor - Sickle cell trait

HC1 to 5 were used as healthy biopsies as they show no BM infiltration of malignant cells.

Table S2. Related to Figure 3. Treatment protocols of patients in Table S1

AML ID	Treatment protocol	Alive
AML11	Induction: AraC, daunorubicin and etoposide	yes
AML12	Induction: ifosfomide, carboplatin and etoposide (ICE) and AraC	yes
AML13	Induction: AraC, daunorubicin and etoposide	no
AML14	Induction: AraC, daunorubicin and etoposide	yes
AML15	Induction: AraC, daunorubicin and etoposide<br>Consolidation: all trans retinoic acid, 6-mercaptopurine and methotrexate	yes
AML16	Induction 1: ifosfomide, carboplatin and etoposide (ICE)<br>Induction 2: daunorubicin and AraC	no
AML17	Induction: idarubicin and AraC	no
AML18	Induction: ifosfomide, carboplatin and etoposide (ICE)	yes
AML19	Induction: AraC, daunorubicin and etoposide	no
AML20	Induction: Daunorubicin and AraC + haematopoetic stem cell transplant	yes
AML21	Induction 1: German multicentre acute lymphoblastic leukeamia trial<br>Induction 2: etoposide and AraC<br>Relapse: intrathecal chemotherapy to treat central nervous system relapse	no
AML22	Induction: AraC, daunorubicin and etoposide + Gemtuzumab ozogamacin<br>Relapse 1: Low dose AraC<br>Replapse 2: daunorubicin and AraC	no
AML23	Induction: AraC, daunorubicin and etoposide	no
AML24	Induction: daunorubicin and AraC + all trans retinoic acid	yes
AML25	Induction: daunorubicin and AraC + Gemtuzumab ozogamacin	yes
AML26	Induction: all trans retinoic acid and arsenic trioxide	yes
AML27	Induction: Azacytidine + low dose AraC<br>Consolidation: low dose AraC	no
AML28	Induction 1: AraC, daunorubicin and etoposide	no
AML29	Induction: Azacytidine	no

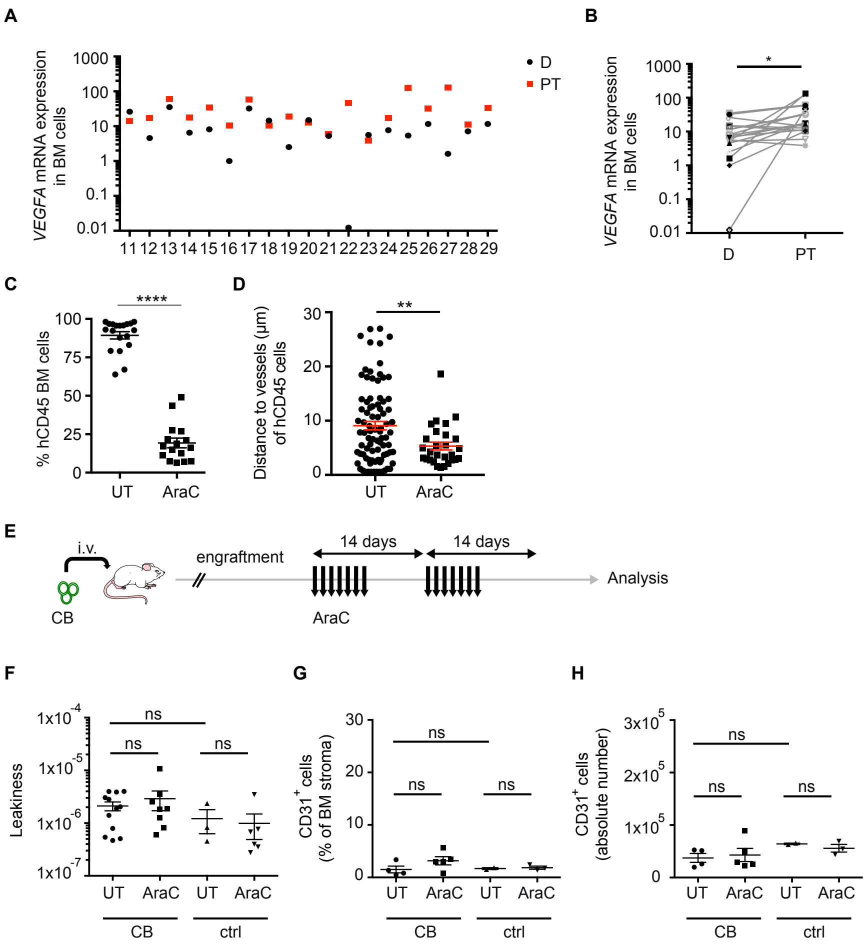

Figure S3. Related to Figure 3. Persistence of increased vascular permeability after chemotherapy. (A) *VEGFA* mRNA relative expression in BMC derived from AML patients at diagnosis (D, black circles) and post-treatment (PT, red squares). **(B)** Comparison of the level of *VEGFA* mRNA relative expression in BMC of each AML patients at diagnosis and post-treatment. **(C)** Percentage of human CD45^{+} leukemic cells in the BM of mice described, treated with AraC or control solvent. AML patients tested: AML6, 7, 8, 9. UT n=17; AraC n=16. Each dot represents one mouse. Bars represent mean ± s.e.m. **(D)** Measurement of hCD45^{+} cell distribution in relation to vasculature in the calvaria BM of mice transplanted with AML6 patient-derived cells, and treated or not with AraC, as depicted. Each dot represents one cell. Bars represent mean± s.e.m. UT n=86; AraC n=28. **(E)** Schematic of the experiment. Non-transplanted mice or mice engrafted with human CD34^{+} cells were treated with 10 mg/kg/day of AraC or solvent, administrated intraperitoneally for two alternated weeks. Vessel-pooling agents were administrated intravenously. Mice were sacrificed 10 min later and calvaria imaged via 2P microscopy. **(F)** Vascular leakiness in the BM of mice detailed in A; n=3 or more replicates. Bars represent mean ± s.e.m. **(G)** Quantification of CD31^{+} endothelial cells in the BM (shown as % of CD45^{-} Ter119^{-} BM cells) of mice detailed in A. Bars represent mean ± s.e.m. **(H)** Absolute number of CD31^{+} endothelial cells in the BM (2 femurs, 2 tibias and 2 iliac crests) of mice detailed in A, as depicted. Bars represent mean ± s.e.m. p values: ns: non-significant; * p<0.05; ** p<0.01;*** p<0.001.

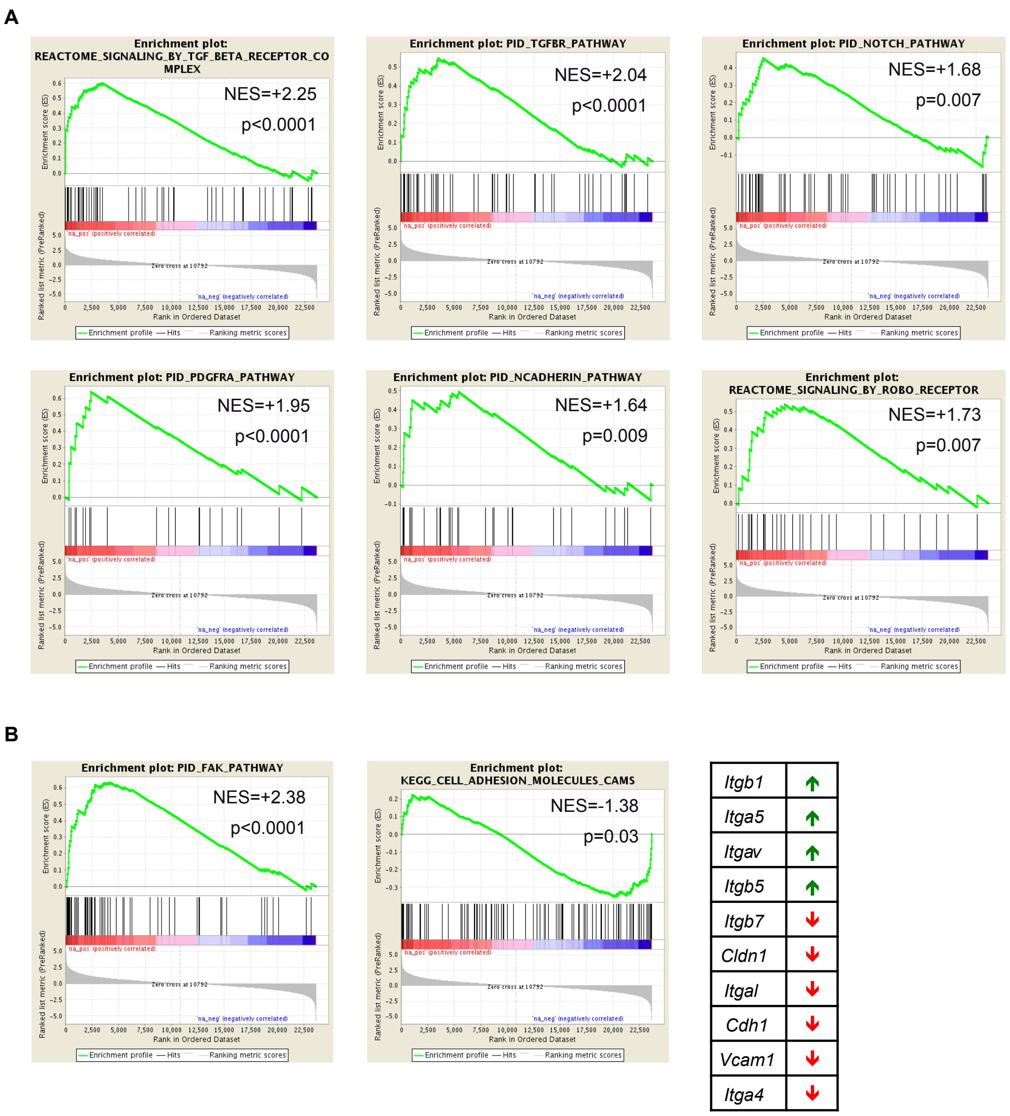

Figure S4. Related to Figure 4. AML-induced transcriptional signature in BM ECs. (A) Enrichment plots for Pathways from GSEA between CB and AML groups using RNA-seq gene expression data indicate enrichment of different pro-angiogenic pathways in endothelial cells from AML group. Normalized Enrichment Score (NES) and nominal p value are shown. **(B)** Enrichment plots for Pathways from GSEA between CB and AML groups using RNA-seq gene expression data indicate upregulation of FAK pathway (top) and downregulation of cell adhesion molecules (CAMs) pathway (bottom) in endothelial cells from the AML group. Normalized Enrichment Score (NES) and nominal p values are shown. On the right, specific deregulated adhesion molecules.

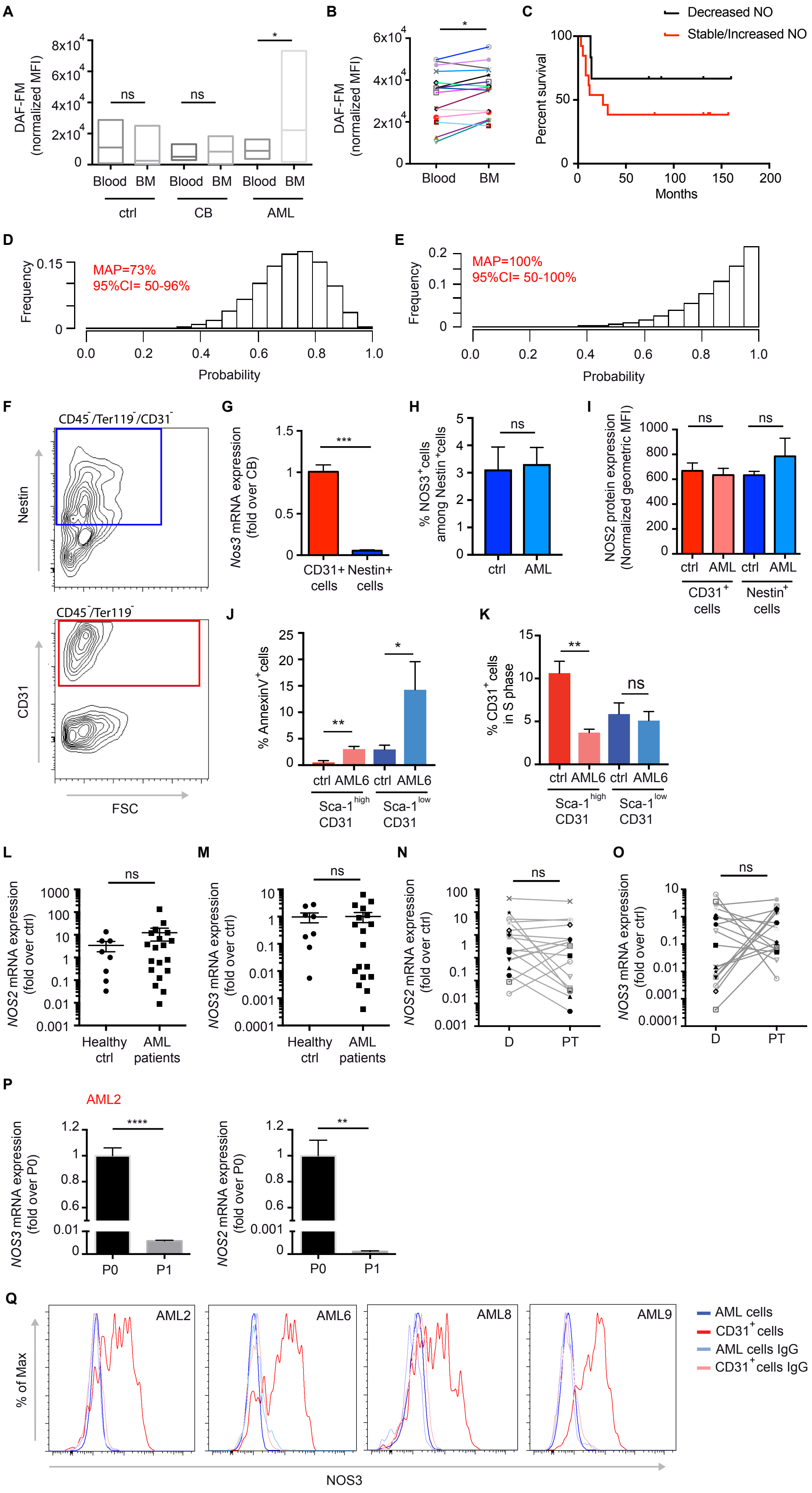
A
DAF-FM (normalized MFI)
ns
ns
*
Blood
BM
ctrl
CB
AML
B
DAF-FM (normalized MFI)
*
Blood
BM
C
Decreased NO
Stable/Increased NO
Percent survival
Months
D
Frequency
MAP=73%
95%CI= 50-96%
Probability
E
Frequency
MAP=100%
95%CI= 50-100%
Probability
F
CD45-/Ter119-/CD31-
Nestin
CD45-/Ter119-
CD31
FSC
G
Nos3 mRNA expression (fold over CB)
***
CD31+ cells
Nestin+ cells
H
% NOS3+cells among Nestin+cells
ns
ctrl
AML
I
NOS2 protein expression (Normalized geometric MFI)
ns
ns
ctrl
AML
CD31+ cells
Nestin+ cells
J
% AnnexinV+cells
**
*
ctrl
AML6
Sca-1high CD31
Sca-1low CD31
K
% CD31+ cells in S phase
**
ns
ctrl
AML6
Sca-1high CD31
Sca-1low CD31
L
NOS2 mRNA expression (fold over ctrl)
ns
Healthy ctrl
AML patients
M
NOS3 mRNA expression (fold over ctrl)
ns
Healthy ctrl
AML patients
N
NOS2 mRNA expression (fold over ctrl)
ns
D
PT
O
NOS3 mRNA expression (fold over ctrl)
ns
D
PT
P
AML2
NOS3 mRNA expression (fold over P0)
****
P0
P1
NOS2 mRNA expression (fold over P0)
**
P0
P1
Q
AML2
AML6
AML8
AML9
% of Max
NOS3
AML cells
CD31+ cells
AML cells IgG
CD31+ cells IgG

Figure S5. Related to Figure 5. AML engraftment is associated to increased nitric oxide levels in the BM. (A) Nitric oxide levels in the BM and peripheral blood cells of non-transplanted mice (ctrl, n=13 and n=9 respectively), mice transplanted with CB derived $CD34^+$ cells (CB, n=24 and n=7 respectively) and AML patients' derived samples (AML2, 6, 9; n=10 and n=8 respectively), as depicted. Data are shown as min to max boxplots, the line inside the box representing the mean. **(B)** Nitric oxide levels in peripheral blood and BM cells of AML patients at diagnosis. Each point represents one patient; n=19. **(C)** Klapan-Meier survival curve of patients stratified based on the NO level normalization parameter. **(D)** Estimated distribution of probabilities of stable/increased NO levels in the BM in case of therapy failure, analyzed in our entire patient cohort (n=19) or **(E)** analyzed in intermediate risk group patients from our cohort (n=9). MAP= maximum a posteriori estimate. CI95= 95% credible interval. Bayesian inference. **(F)** Gating strategy in the $CD45^-Ter119^-$ BM stromal compartment for selecting $Nestin^+$ (top, blue box) and $CD31^+$ (down, red box) cells. **(G)** *Nos3* expression analyzed by qRT-PCR in $CD31^+$ and $Nestin^+$ cells BM cells, as depicted. Data are shown as mean ± s.e.m. **(H)** NOS3 protein expression in $Nestin^+$ mesenchymal cells in the BM of non-transplanted mice or AML xenografts, quantified by flow cytometry. Data are shown as mean ± s.e.m. **(I)** NOS2 protein expression analyzed by flow cytometry (Geometric MFI) in $CD31^+$ and $Nestin^+$ BM cells retrieved from non-transplanted mice or mice engrafted with AML patients-derived samples (AML6, 8 and 9). Data are shown as mean ± s.e.m. **(J)** % of apoptotic cells among different populations of BM-derived ECs, as depicted. Data are shown as mean ± s.e.m. **(K)** % cells in the S phase of cell cycle among different populations of BM-derived ECs, as depicted. Data are shown as mean ± s.e.m. **(L, M)** *NOS2* (L) and *NOS3* (M) expression analyzed by qRT-PCR in the BM of healthy controls (n=8) and in AML patients (n=19) from our cohort (Table S1). Data are shown as mean ± s.e.m. **(N, O)** *NOS2* (N) and *NOS3* (O) expression analyzed by qRT-PCR in the BM of AML patients (n=19) before and after therapy. **(P)** *NOS3* (left) and *NOS2* (right) expression analyzed by qRT-PCR in AML2 patient's derived cells (P0) and AML2 patient's derived xenografted cells (P1), as depicted. Data are shown as mean ± s.e.m. **(Q)** NOS3 protein expression measured via flow cytometry in $CD31^+$ and AML cells in the BM of AML xenografts, as depicted. Data are representative of triplicates. P values: ns: non-significant; *p<0.05; ** p<0.01;*** p<0.001.

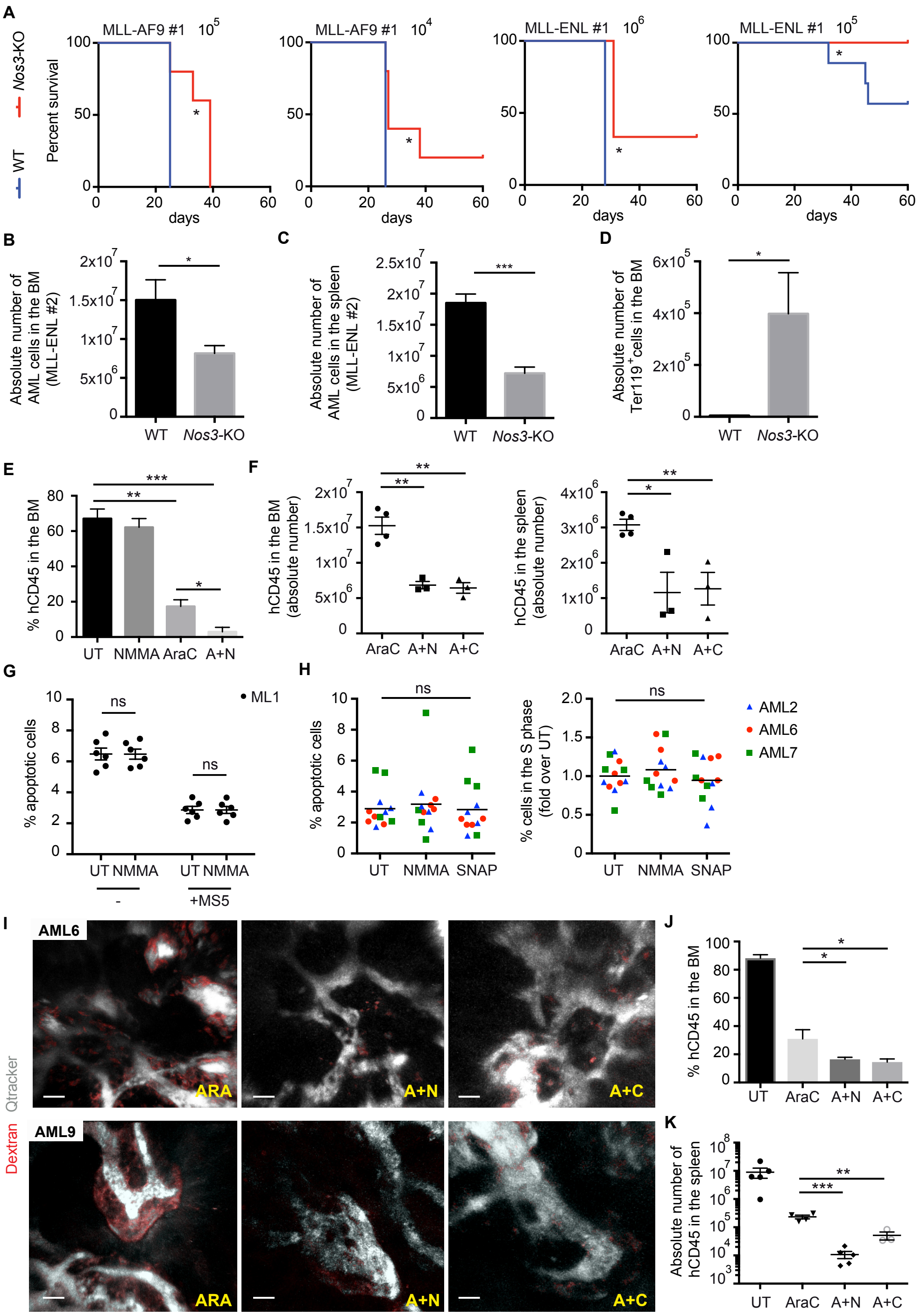

L

Patient sample	Treatment group	BM engraftment (% of hCD45 cells) ± s.e.m.	Spleen engraftment (% of hCD45 cells) ± s.e.m.
AML 6	UT	96.38 ± 0.423; n=3	71.53 ± 3.135; n=3
AML 6	NMMA	95.83 ± 0.088; n=3	56.2 ± 1.418; n=3
AML 6	CAV	95.08 ± 0.118; n=3	41.47 ± 0.393; n=3
AML 6	ARA	16.93 ± 1.01; n=3	10.56 ± 2.878; n=3
AML 6	A+N	7.043 ± 1.201; n=3	6.86 ± 3.164; n=3
AML 6	A+C	5.758 ± 0.126; n=3	3.183 ± 0.136; n=3
AML 9	UT	88.22 ± 2.463; n=5	70.56 ± 4.237; n=5
AML 9	NMMA	89.38 ± 2.453; n=5	77.48 ± 2.053; n=5
AML 9	CAV	89.36 ± 4.63; n=5	73.22 ± 4.475; n=5
AML 9	ARA	30.97 ± 6.527; n=3	48.87 ± 3.012; n=3
AML 9	A+N	16.48 ± 1.433; n=5	8.528 ± 2.541; n=5
AML 9	A+C	14.54 ± 2.158; n=4	25.85 ± 5.322; n=4

Figure S6. Related to Figure 6. Targeting vascular permeability cooperates with chemotherapy to improve AML treatment. (A) Survival curve of mice of depicted genotypes transplanted with murine MLL-AF9 #1 (top) or MLL-ENL #1 (bottom) AML cells at different doses. MLL-AF9 #1: 10^5 WT n=3; *Nos3*-KO n=5. 10^4 WT n=3; *Nos3*-KO n=5; MLL-ENL #1: 10^6 WT n=3; *Nos3*-KO n=3; 10^5 WT n=7; *Nos3*-KO n=4; 10^4 WT n=3; *Nos3*-KO n=3. **(B)** Absolute number of MLL-ENL #2 tomato$^+$ leukemic cells in the BM of mice of depicted genotypes. Data are shown as mean± s.e.m. **(C)** Absolute number of tomato$^+$ MLL-ENL leukemic cells in the spleen of mice of depicted genotypes. Data are shown as mean± s.e.m. **(D)** Absolute of Ter119$^+$ BMC in mice of depicted genotypes engrafted with tomato+ MLL-ENL leukemic cells. Data are shown as mean± s.e.m. **(E)** Human CD45$^+$ engraftment in the BM of mice engrafted with ML1 human AML cell line and treated with solvent (UT), NMMA alone, AraC alone, or combination of AraC with NMMA (A+N), as depicted; n=3. Data are shown as mean ± s.e.m. **(F)** Absolute number of human CD45$^+$ cells in the BM (left) and the spleen (right) of mice engrafted with ML.1 treated with AraC alone, or combination of AraC with NMMA (A+N) or Cavtatrin (A+C), as depicted; n=3 or more. Bars represent mean ± s.e.m. **(G)** % of apoptotic of ML1 cells grown alone or in co-culture with MS-5 and treated or not with NMMA. Bars represents mean ± s.e.m. Each dot represents a well. **(H)** % of apoptotic cells (left) and cells in the S phase (right) among AML primary patient-derived cells grown in co-culture with MS-5 and treated or not with NMMA and SNAP. Each dot represents one well. Bars represent mean. **(I)** Representative 3D reconstructions of the calvarium BM of mice transplanted with AML6 (top) and AML9 (bottom) patient-derived cells, treated with AraC alone or combination of AraC with NMMA or Cavtatrin, as depicted, and imaged via 2P microscopy using 655-conjugated Qtracker and TRITC-Dextran as vessel pooling agents. Bars respresent 30 µm. **(J)** Percentage of Human CD45$^+$ engraftment in the BM of mice engrafted with patient-derived sample AML9 and treated as in Figure 6D; n=5 per condition. Data are shown as mean ± s.e.m. **(K)** Absolute number of leukemic cells in the spleen of mice engrafted with AML9 patient-derived cells and treated or not with AraC, NMMA, Cavtatrin or combination of AraC and NMMA or Cavtatrin. Bars represent mean ± s.e.m. **(L)** Multi-organ analysis of PDX described in Figure 6 and S6. P values: ns: non-significant; *p<0.05; ** p<0.01; *** p<0.001; ****p<0.0001.

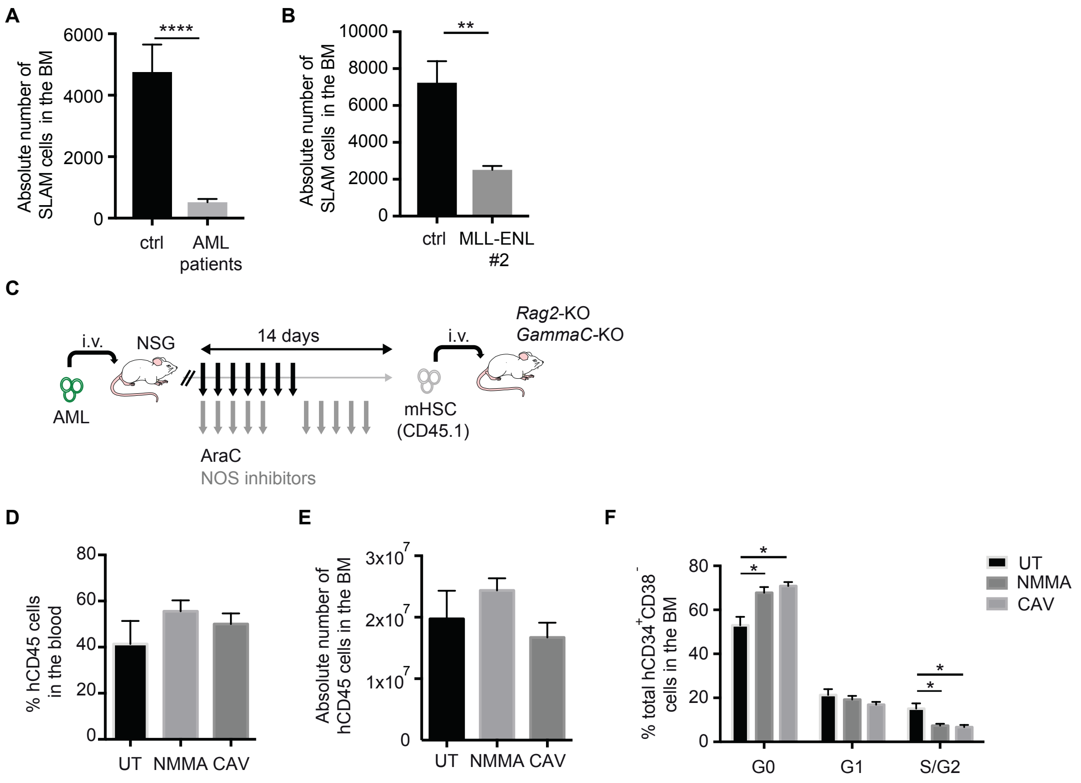

Figure S7. Related to Figure 7. Targeting vascular permeability potentiates HSPC function. (A) Absolute number of murine HSC in the BM of non-transplanted mice (n=5) or mice engrafted with AML patient-derived cells (AML6, n=4; AML9, n=5), as depicted. Data are shown as mean ± s.e.m. **(B)** Absolute number of murine HSC in the BM of non-transplanted mice (n=4) or mice engrafted with murine MLL-ENL leukemia (n=5), as depicted. Data are shown as mean ± s.e.m. **(C)** Schematic of the experiment. Residual murine CD45.1 cells in the BM of mice engrafted with AML6 patient-derived cells and treated with AraC or combination of AraC and NO inhibitors were transplanted i.v. in sub-lethally irradiated *Rag2*-KO/*GammaC*-KO CD45.2 recipient mice. Engraftment was assessed 8 weeks after transplantation (see Figure 7E-G). **(D)** Percentage of $hCD45^+$ cells in the peripheral blood of mice described in Figure 7H. Data are shown as mean ± s.e.m. **(E)** Absolute number of $hCD45^+$ cells in the BM of mice described in Figure 7H. Data are shown as mean ± s.e.m. **(F)** Percentage of HSPC in the different phase of cell cycle in the BM of mice in Figure 7H. Data are shown as mean ± s.e.m. p values: * p<0.05; **p<0.01; **** p<0.0001.
