# Supplementary material for: Increased Vascular Permeability in the Bone Marrow Microenvironment Contributes to Disease Progression and Drug Response in Acute Myeloid Leukemia
Source: Cancer Cell. 2017 Sep 11;32(3):324–341.e6. doi: 10.1016/j.ccell.2017.08.001 (PMC5598545; doi:10.1016/j.ccell.2017.08.001)
Supplement: Document S2. Article plus Supplemental Information [file mmc2.pdf]

Cancer Cell

Article

Increased Vascular Permeability in the Bone Marrow Microenvironment Contributes to Disease Progression and Drug Response in Acute Myeloid Leukemia

Graphical Abstract

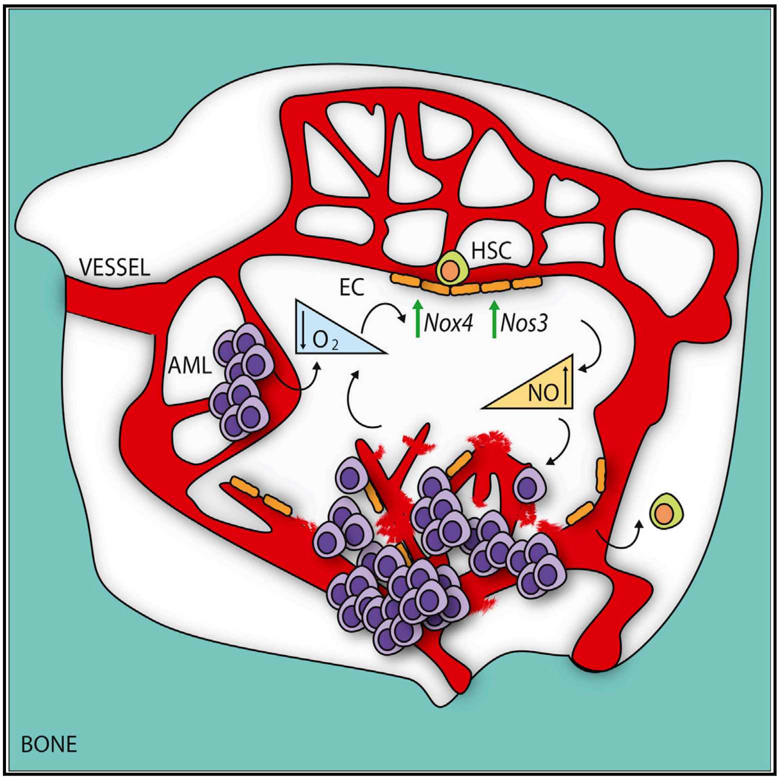

Highlights

- Functional imaging of the BM vascular niche using intravital 2P microscopy
- Increased vascular permeability is caused by robust molecular alterations in ECs
- Persistent high NO after chemotherapy is associated with poor prognosis in patients
- Inhibition of EC-derived NO normalizes vasculature and improve response to Ara.C

Authors

Diana Passaro, Alessandro Di Tullio, Ander Abarrategi, ..., François Lassailly, John Gribben, Dominique Bonnet

Correspondence

dominique.bonnet@crick.ac.uk

In Brief

Passaro et al. show that acute myeloid leukemia, mainly via nitric oxide (NO), causes bone marrow vascular abnormalities and that failure to restore normal vasculature after induction chemotherapy is associated with a poor prognosis. Importantly, inhibition of NO production improves treatment response *in vivo*.

Data Resources

GSE88905

Passaro et al., 2017, Cancer Cell *32*, 324–341
September 11, 2017 © 2017 The Francis Crick Institute.
Published by Elsevier Inc.
http://dx.doi.org/10.1016/j.ccell.2017.08.001

CellPress
Cancer Cell
Article

Increased Vascular Permeability in the Bone Marrow Microenvironment Contributes to Disease Progression and Drug Response in Acute Myeloid Leukemia

Diana Passaro,[1] Alessandro Di Tullio,[1] Ander Abarrategi,[1] Kevin Rouault-Pierre,[1] Katie Foster,[1] Linda Ariza-McNaughton,[1] Beatriz Montaner,[1] Probir Chakravarty,[2] Leena Bhaw,[3] Giovanni Diana,[4] François Lassailly,[1] John Gribben,[5] and Dominique Bonnet[1,6,*]
[1]Haematopoietic Stem Cell Laboratory, The Francis Crick Institute, 1 Midland Road, London NW1 1AT, UK
[2]Bioinformatic Core Unit, The Francis Crick Institute, 1 Midland Road, London NW1 1AT, UK
[3]Advanced Sequencing Unit, The Francis Crick Institute, 1 Midland Road, London NW1 1AT, UK
[4]MRC Centre for Developmental Neurobiology, King's College London, London, UK
[5]Department of Haemato-Oncology, Barts Cancer Institute, Queen Mary University of London, London EC1M 6BQ, UK
[6]Lead Contact
*Correspondence: dominique.bonnet@crick.ac.uk
http://dx.doi.org/10.1016/j.ccell.2017.08.001

SUMMARY

The biological and clinical behaviors of hematological malignancies can be influenced by the active crosstalk with an altered bone marrow (BM) microenvironment. In the present study, we provide a detailed picture of the BM vasculature in acute myeloid leukemia using intravital two-photon microscopy. We found several abnormalities in the vascular architecture and function in patient-derived xenografts (PDX), such as vascular leakiness and increased hypoxia. Transcriptomic analysis in endothelial cells identified nitric oxide (NO) as major mediator of this phenotype in PDX and in patient-derived biopsies. Moreover, induction chemotherapy failing to restore normal vasculature was associated with a poor prognosis. Inhibition of NO production reduced vascular permeability, preserved normal hematopoietic stem cell function, and improved treatment response in PDX.

INTRODUCTION

Acute myeloid leukemia (AML) is the most common acute leukemia in adults (Sant et al., 2010). AML is a hematological malignancy arising from the occurrence of genetic mutations in hematopoietic progenitors, which cause a blockage in the maturation and an uncontrolled growth of leukemic blasts in the bone marrow (BM). While the clinical presentation of AML is quite uniform, it is a highly heterogeneous disease at the molecular level. There is a consistent effort aimed to characterize each molecular subgroup and design the best therapeutic strategy (Grimwade et al., 2010). Besides a few cases (Wang and Chen, 2008), the common clinical practice remains induction therapy with cytarabine (AraC) (Buchner et al., 2012; Mayer et al., 1994). However, therapy resistance and relapse remain a major clinical challenge.

Another approach aims to identify and target common features within this complex disease. One possibility could be the BM microenvironment, which is the site where leukemic cells arise, expand, and eventually develop resistance to therapy. The BM microenvironment is constituted by different types of cells, with a predominant vascular component responsible for nutrient and metabolite turnover, the ingress and egress of different cells, and the regulation of normal hematopoietic

Significance

Acute myeloid leukemia (AML) represents the most common acute leukemia affecting adults. Recent findings suggest that AML cells alter their microenvironment, which represents an intriguing source of potential therapeutic targets. A thorough characterization of the vasculature of the bone marrow with AML reveals a severe functional abnormality. Vascular wall barriers responsible for oxygen, nutrients, and drug delivery appear severely damaged, with increased permeability, altered perfusion, and release of normal hematopoietic stem cells to the periphery. At a molecular level, endothelial cell signature is altered, resulting in increased reactive oxygen species and nitric oxide. In our preclinical models, the successful combination of nitric oxide synthase inhibitors and chemotherapy restore normal vasculature and delay leukemia, leading the way to combine leukemia-niche therapies in clinical trials.

 © 2017 The Francis Crick Institute. Published by Elsevier Inc.
This is an open access article under the CC BY license (http://creativecommons.org/licenses/by/4.0/).

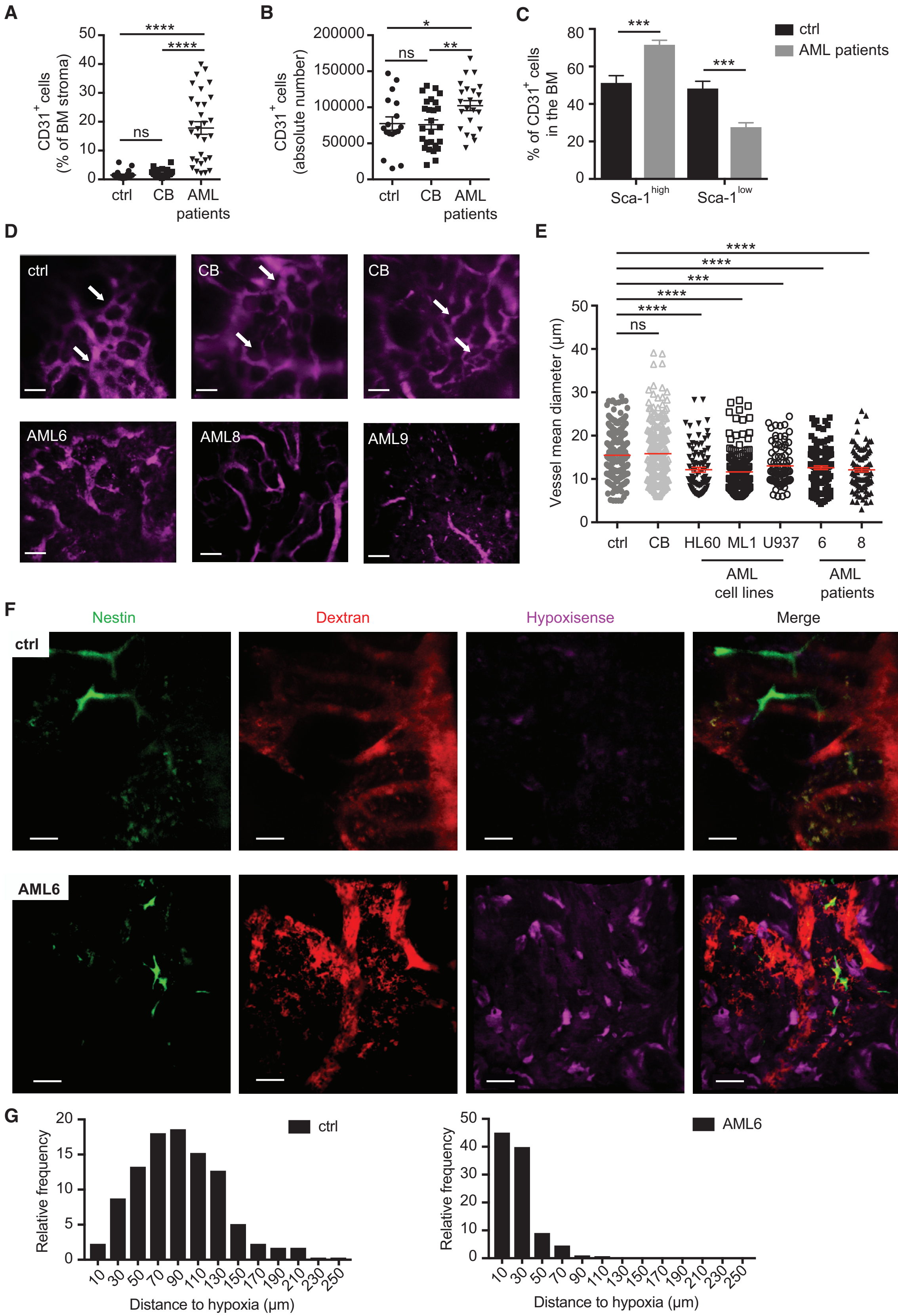

(legend on next page)

Table 1. Patient-Derived Samples Used for Xenograft Studies

AML ID	Cytogenetics	Cyto Risk	NPM	FLT3
AML1	ND[a]	ND	mutant	ND
AML2	t(9,6)	intermediate	ND	ND
AML3	normal	intermediate	WT	ITD[b]
AML4	11q23/MLL	ND	ND	ND
AML5	complex	poor	WT	ND
AML6	normal	intermediate	mutant	ITD
AML7	normal	intermediate	mutant	ITD
AML8	Del 7q+8 + 1	poor	WT	WT
AML9	normal	intermediate	mutant	ITD
AML10	complex	poor	ND	ND

[a]Not determined.
[b]Internal tandem duplication.

stem cell (HSC) function (Boulais and Frenette, 2015; Mendez-Ferrer et al., 2015). The introduction and development of intravital two-photon (2P) microscopy has allowed direct, high-resolution, dynamic imaging of the calvarium BM (Colmone et al., 2008; Foster et al., 2015; Lassailly et al., 2013; Lo Celso et al., 2009; Sipkins et al., 2005), providing a powerful tool for the functional characterization of the BM microenvironment.

Recent findings indicate that myeloid malignancies also affect the function of the BM niche, pointing to the existence of an active crosstalk between leukemic cells and the microenvironment (Dong et al., 2016; Frisch et al., 2012; Hanoun et al., 2014; Kim et al., 2008; Kode et al., 2014; Krause et al., 2013; Raaijmakers et al., 2010; Schepers et al., 2013; Schmidt et al., 2011; Zhang et al., 2012). Similar to what has been observed in solid cancers, AML has been associated with an increase in microvascular density (MVD) and production of pro-angiogenic factors, notably vascular endothelial growth factor (VEGF) (Chand et al., 2016; Hussong et al., 2000; Kampen et al., 2013). However, whether increased MVD and VEGF constituted a prognostic factor for AML treatment response remains unclear (Aref et al., 2005; Padro et al., 2000; Reddy and Moreb, 2000). Moreover, despite promising preclinical studies (Zhu et al., 2003), clinical trials incorporating anti-VEGF inhibitors have not produced encouraging results (Fiedler et al., 2003, 2005; Giles et al., 2006; Mattison et al., 2015; Roboz et al., 2006; Zahiragic et al., 2007), suggesting that targeting pro-angiogenic cytokines may not be the best strategy for disrupting the crosstalk between AML and the vascular niche. A thorough analysis of the status of the vasculature in the BM is required to dissect the complexity of the vascular phenotype associated with AML. Thus, we aim to study the *in vivo* picture of the abnormalities associated with the BM vasculature induced by AML engraftment and unravel common pathologic processes, which could represent potential targets in AML.

RESULTS

AML Engraftment Alters Vascular Architecture and Function

To provide a detailed picture of the BM vasculature in AML, we studied the status of the vascular niche in human AML patient-derived xenografts (PDX). Recipient mice were left unconditioned, given the toxic effect derived from the irradiation or myelosuppressive treatment on the vasculature (Hooper et al., 2009; Kopp et al., 2005; Shirota and Tavassoli, 1991; and data not shown). We observed an expansion of the endothelial compartment among the non-hematopoietic stroma upon human AML engraftment (Figure 1A and Table 1). Importantly, this effect was specific to AML, as no such expansion was observed in mice engrafted with normal human hematopoietic stem/progenitor cells (HSPCs) derived from umbilical cord blood (CB) (Figure 1A). Moreover, the percentage of endothelial cells (ECs) was positively correlated to the leukemic engraftment of human AML cell lines and patient-derived samples (Figure S1A), suggesting a gradual pathologic process. Not only did the percentage of ECs increase, but there was also a real expansion of the endothelial compartment in terms of absolute number, specifically upon human AML engraftment (Figure 1B). We also observed an increased MVD, as shown by the higher number of vessel sprouts quantified by immunofluorescence (Figures S1B and S1C), similar to what is observed in patient-derived trephines (Chand et al., 2016; Padro et al., 2000). The existence of

Figure 1. AML-Derived Alteration of the BM Vascular Architecture and Function

(A) Quantification of $CD31^+$ ECs in the BM (shown as percentage of $CD45^-Ter119^-$ BM cells) of non-transplanted mice (ctrl) and mice transplanted with HSPCs (CB) or AML patient-derived samples, as depicted. Each dot represents an individual mouse. Ctrl, n = 22; CB, n = 29; AML patients (AML1, 2, 3, 5, 6, 7, 8, 9), n = 30. Data are shown as mean ± SEM.

(B) Absolute number of $CD31^+$ ECs in the BM (2 femurs, 2 tibias, and 2 iliac crests) of non-transplanted mice (ctrl) and mice transplanted with CB-derived HSPCs (CB) or AML patient-derived samples, as depicted. Each dot represents an individual mouse. Ctrl, n = 17; CB, n = 26; AML patients (AML1, 2, 6, 7, 8, 9), n = 23. Data are shown as mean ± SEM.

(C) Frequency of $Sca\text{-}1^{high}$ and $Sca\text{-}1^{low}$ $CD31^+$ cells in the BM of non-transplanted mice (ctrl) and mice transplanted with AML patient-derived samples. Ctrl, n = 19; AML patients (AML 2, 3, 6, 8, 9), n = 16. Data are shown as mean ± SEM.

(D) Representative 3D reconstruction of BM vasculature of the calvarium of non-transplanted mice (ctrl) and mice transplanted with CB-derived HSPCs (CB) or AML patient-derived samples, as depicted, imaged via 2P microscopy 1 min after injection of 655-conjugated NT-Qtracker as vessel-pooling agent. White arrows pointing at sinusoids. Data are representative of triplicates in 4 independent experiments. Scale bars represent 70 μm.

(E) Quantification of vascular mean diameter in the calvarium BM of non-transplanted mice (ctrl) and mice transplanted with CB-derived HSPCs (CB) or human AML-derived samples, as depicted, using IMARIS filament tool. Dots represent the diameter of vascular fragments in the z stack of the calvarium of at least 3 mice per group. Ctrl, n = 235; CB, n = 232; HL60, n = 91; ML1, n = 148; U937, n = 85; AML6, n = 130; AML8, n = 94. Red lines represent the mean ± SEM.

(F) Representative 3D reconstruction of BM hypoxia imaged via intravital microscopy using the HypoxiSense probe together with vasculature (dextran) and $Nestin^+$ cells in non-transplanted mice or mice transplanted with AML6 patient-derived cells, as depicted. Scale bars represent 50 μm.

(G) Distribution and relative frequency of vessel distances to hypoxic areas in the BM of non-transplanted mice (ctrl) or mice transplanted with AML6 patient-derived cells, as depicted.

ns, not significant; *p < 0.05; **p < 0.01, ***p < 0.001, ****p < 0.0001. See also Figure S1.

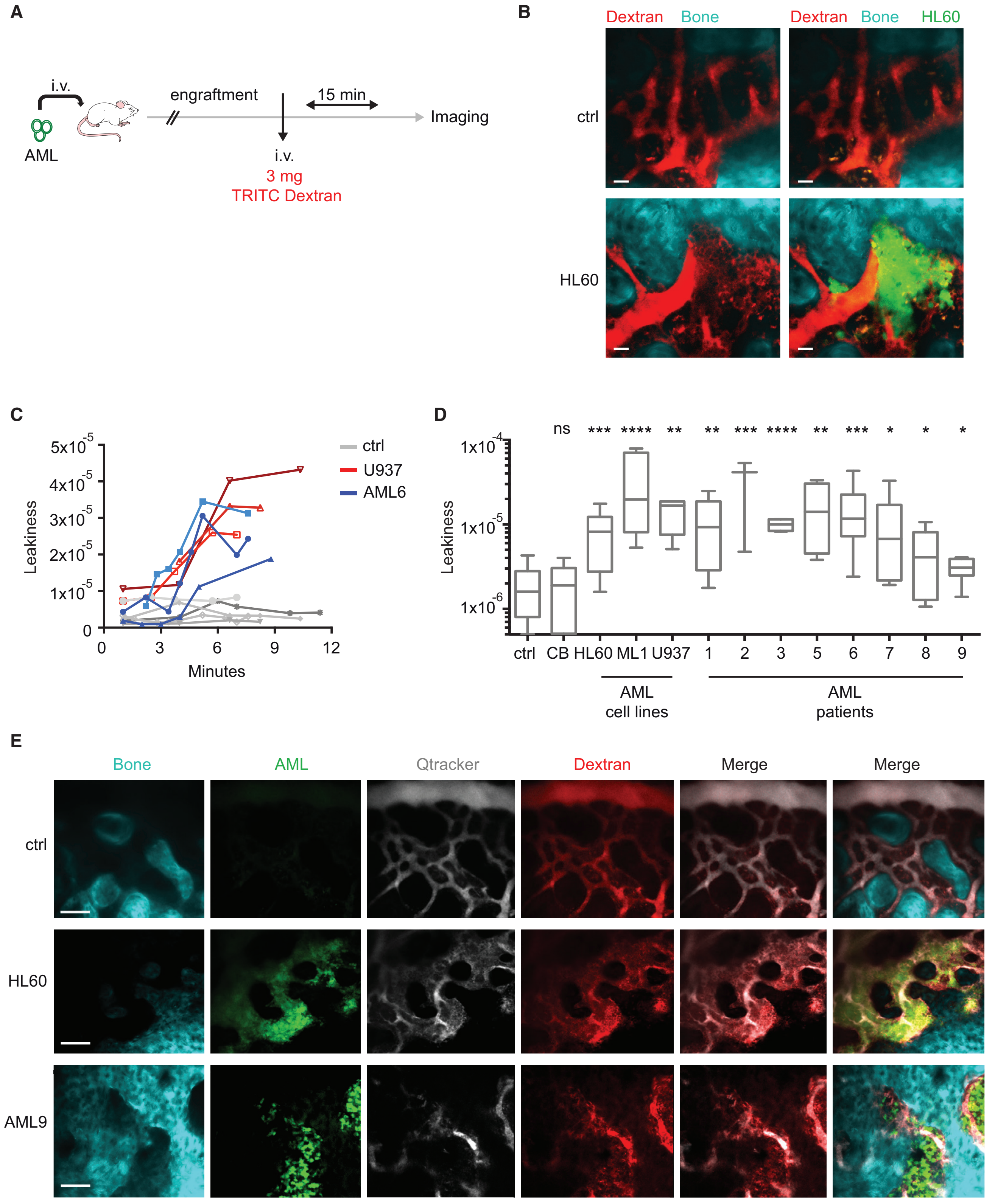

(legend on next page)

specific endothelial cell markers defining distinct BM vascular niches has recently been highlighted (Itkin et al., 2016). We thus analyzed the expression of these markers in the context of AML disease in PDX. We observed a significant loss of ECs associated with sinusoids ($CD31^{+}Sca1^{low}$) as well as an increased number of ECs associated with arterioles ($CD31^{+}$ $Sca1^{high}$) (Figures 1C and S1D). We next analyzed the architecture of the BM vasculature by 2P microscopy using a vessel-pooling agent to visualize the vascular tree in the calvarium BM. Although vascular architecture appeared highly heterogeneous among different PDX (Figures 1D and S1E), we noticed some common abnormalities. First, the regularity of sinusoidal structures, which are preserved with normal human engraftment, was lost in human AML xenografts (Figure 1D, white arrows pointing at sinusoids). Second, the mean vascular diameter of vessels was reduced (Figure 1E), a pathologic phenotype previously reported in tumor angiogenesis as a result of solid stress applied to vessels by overgrowing tumor cells (Padera et al., 2004; Stylianopoulos and Jain, 2013). Vessel compression was also highlighted by H&E staining in long bones (Figure S1F, dashed circles indicating vessel lumen). To study BM perfusion, we injected isolectin B4 (IB4), a pan-endothelial marker (Lassailly et al., 2013), and analyzed its distribution on the BM vasculature by 2P microscopy. In control mice, we observed a homogeneous IB4 perfusion rate, allowing the visualization of ECs surrounding the arteriolar and sinusoidal vasculature (Figure S1G, ctrl). In contrast, we observed the presence of many poorly perfused areas in the BM of AML xenografts (Figure S1G). We next tested whether AML engraftment also affected BM oxygenation, by measuring the BM hypoxia. While in non-transplanted mice we observed a heterogeneous staining with Hypoxyprobe, indicating a physiological spread distribution of hypoxic areas, human AML engraftment increased the hypoxia homogeneously throughout the bones (Figure S1H). Quantification of Hypoxyprobe staining in BM cells by flow cytometry confirmed the significant increase of BM hypoxia upon human AML engraftment compared with normal human engraftment (Figure S1I). Of note, at early stage of engraftment hypoxia was localized in close proximity to AML cells (Figures S1J–S1M), whereas at high engraftment the BM was overall hypoxic (Figures S1N–S1P). We next visualized the hypoxic state of the BM via intravital microscopy using HypoxiSense probe (Bao et al., 2012). Similar to what was observed with Hypoxyprobe, we observed increased hypoxia in the BM upon AML engraftment with this alternative method (Figures 1F, S1Q, and S1R). To evaluate the hypoxic state of the vasculature, we measured the distance of each vessel to hypoxic areas. Whereas in control BM this distance was widely distributed, in the presence of AML most of the vessels were close to hypoxic areas (Figures 1G and S1S). Together these results showed that AML is associated with a structurally and functionally abnormal vasculature in the BM.

AML Engraftment Increases Vascular Permeability in the BM

To study the vascular barrier function in the BM of human AML xenografts, we used intravital 2P microscopy to image the calvarium of unconditioned NSG mice engrafted with different human AML cell lines or patient-derived samples. Human AML cell lines were engineered to express GFP and luciferase to facilitate the monitoring of BM engraftment via bioluminescence (Figure S2A) and BM aspirate (Figure S2B). Prior to imaging, tetramethylrhodamine isothiocyanate (TRITC)-dextran was injected intravenously to label the vasculature (Egawa et al., 2013; Fukumura and Jain, 2008) (Figure 2A). Strikingly, we observed an important leakage of the dextran outside the vasculature in the BM of mice engrafted with human AML cell lines compared with control non-transplanted mice (Figures 2B and S2C). This observation was corroborated by H&E staining of long bones showing the loss of vascular barriers and release of erythrocytes in the BM parenchyma (Figure S2D, red arrows pointing at erythrocytes). To quantify the vascular leakiness, we used a strategy allowing reliable measurement independent of the fluorescence intensity of the probe (detailed in Figure S2E and STAR Methods). The intravital observation of the calvarium BM vasculature at different time points after injection of the vessel-pooling agents showed an increased vascular leakiness starting between 6 and 9 min post injection (Figure 2C). We quantified vascular permeability in xenografts of different human AML samples 10 min after injection of the vessel-pooling agents (Figures 2D and 2E). All the samples used in our study, which represented different molecular subtypes of human AML, induced increased vascular leakiness in the BM. A different method using *in vivo* Hoechst permeability in BM cells (Wong et al., 2015) was used to confirm the AML-associated vascular phenotype in the BM (Figures S2F and S2G). Of note, AML-engrafted areas appeared to be much more leaky than

Figure 2. AML Engraftment Increases Vascular Permeability in the BM

(A) Schematic of the experiment. Human AML cells engineered to express GFP and luciferase were injected intravenously into NSG mice and engraftment was monitored via bioluminescence and BM aspirate. Once engraftment was confirmed, 3 mg of 65–85 kDa TRITC-dextran was injected intravenously and 15 min later, calvaria were imaged via 2P microscopy.

(B) Representative z stack of the TRITC-dextran-labeled BM vasculature in the calvarium of non-transplanted control or from mice transplanted with GFP-HL60 cells. Left: TRITC-dextran signal alone. Right: merge of TRITC-dextran and GFP signals. Data are representative of triplicates. Scale bars represent 30 μm.

(C) Vascular leakiness quantified *in vivo* at different time points after dextran injection in non-transplanted mice (ctrl) and U937 or AML6 patient cells-engrafted mice.

(D) Vascular leakiness quantification in the BM of non-transplanted mice or mice transplanted with different human AML cell lines and patient-derived samples, as depicted, and imaged 10 min after administration of vascular dyes. Ctrl, n = 11; CB, n = 19; HL60, n = 12; ML1, n = 7; U937, n = 4; AML9, n = 6; AML6, n = 14; AML5, n = 4; AML1, n = 12; AML3, n = 4; AML2, n = 3; AML8, n = 7; AML7, n = 6. Data are shown as whiskers minimum-to-maximum plots, the line inside the box representing the mean, the top and the bottom lines representing the 75% and 25% percentiles, respectively, and the lines above and below the box representing the SD.

(E) Representative z stacks of BM vasculature in the calvarium of non-transplanted mice (ctrl) or mice engrafted with HL60 or AML9 cells, as depicted. Scale bars represent 80 μm.

ns, not significant; *p < 0.05, **p < 0.01, ***p < 0.001, ****p < 0.0001. See also Figure S2.

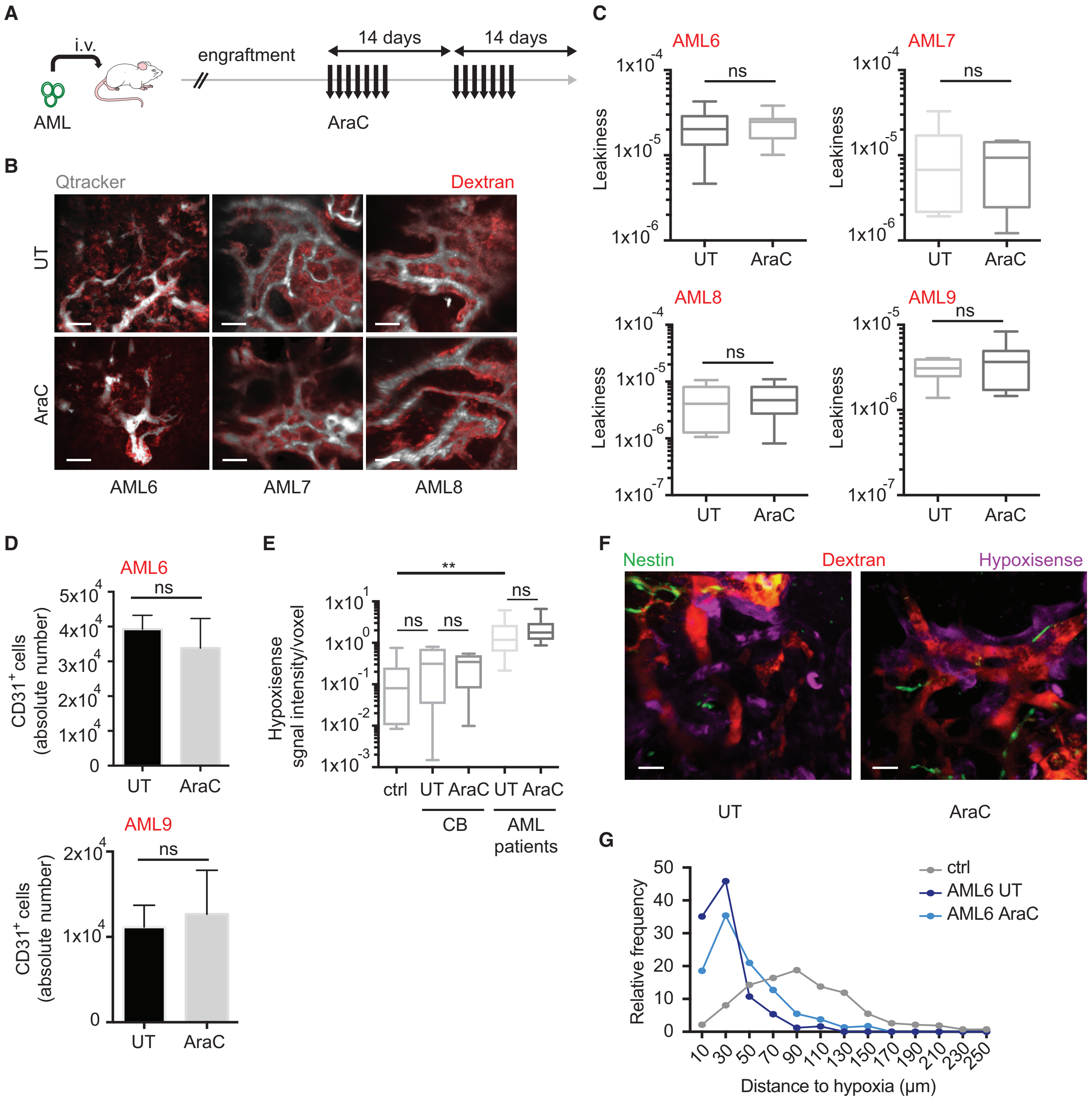

Figure 3. Persistence of Increased Vascular Permeability after Chemotherapy

(A) Schematic of the experiment. Patient-derived AML cells were injected intravenously into NSG mice and engraftment was monitored via BM aspirate. Once BM engraftment (>40%) assessed, 10 mg/kg/day of AraC or solvent was administrated subcutaneously for one or two alternate weeks, depending on the patient sample. Seven days after the last administration of AraC, vessel-pooling agents were administrated intravenously. Mice were euthanized 10 min later and calvaria imaged via 2P microscopy.

(B) Representative 3D reconstruction of the BM vasculature in the calvarium of mice engrafted with different patient-derived samples treated with solvent (top) or AraC (bottom). Scale bars represent 70 µm.

(C) Vascular leakiness in the BM of mice transplanted with different human AML patient-derived samples, treated with solvent or AraC, as depicted. Three or more replicates were used for each condition. Data are shown as whiskers minimum-to-maximum plots, the line inside the box representing the mean, the top and the bottom lines representing the 75% and 25% percentiles, respectively, and the lines above and below the box representing the SD.

(D) Absolute number of $CD31^+$ ECs in the BM (n = 2 femurs) of mice transplanted with AML6 (top) or AML9 (bottom) patient-derived cells, treated with solvent or AraC, as depicted; n = 3 in each condition. Data are shown as mean ± SEM.

(E) Hypoxia represented as HypoxiSense signal intensity/voxel in the calvaria BM of non-transplanted mice (ctrl) and mice transplanted with CB-derived HSPCs (CB) or AML patient-derived samples, treated or not with AraC, as depicted. Ctrl, n = 9; CB UT, n = 7; CB AraC, n = 8; AML patients (AML6 and 9) UT, n = 16; AraC,

(legend continued on next page)

non-engrafted areas (Figures S2H and S2I). In normal BM, Nestin-associated vessels, characterized by higher expression of Sca-1 (Itkin et al., 2016), show less permeability than Nestin$^-$ vessels (Figure S2J). On the contrary, in the presence of leukemic engraftment, both Nestin$^+$ and Nestin$^-$ vessels were associated with increased leakiness (Figures S2J and S2K), highlighting a substantial functional difference between normal and leukemic Nestin-associated Sca-1$^+$ vessels. Of note, reduction of normal pericyte coverage was also observed in the BM of AML PDX (Figure S2L). These results show an altered vascular permeability in the BM associated with AML engraftment, observed commonly throughout different AML subtypes.

Chemotherapy Does Not Rescue Normal Vascular Permeability in the BM

It is still debated whether AML-derived angiogenesis, in terms of MVD in the BM and VEGF quantification, is reduced in patients upon chemotherapy (Aref et al., 2005; Chand et al., 2016; Padro et al., 2000; Reddy and Moreb, 2000). Thus, we analyzed the level of VEGF in BM biopsies obtained at diagnosis and post treatment from a cohort of AML patients (Tables S1 and S2). We found an increase in VEGF in the majority of patients' post-treatment biopsies (Figures S3A and S3B). This suggests a persistent pro-angiogenic environment after chemotherapy. We thus studied the vascular niche state in AML PDX after chemotherapy. We set up a preclinical treatment protocol, which closely followed the clinical practice, by treating AML xenotransplanted mice daily with 10 mg/kg of AraC during one or two alternate weeks, depending on xenografts' response (Figure 3A). This treatment significantly reduced the leukemic burden in the BM (Figure S3C) and induced a "remission-like" phase, during which the remaining leukemic cells persist and give rise to relapse after few weeks (not shown). Notably, residual AML cells after AraC treatment localized in close proximity to the vasculature (Figure S3D). We observed the BM vasculature with 2P microscopy during the "remission-like" phase in different cohorts of AML PDX treated with this protocol. Although the leukemic engraftment was significantly reduced, the vascular permeability was still very high, similar to mice treated with the control solvent, which were highly engrafted with leukemic cells (Figures 3B and 3C). Moreover, the number of ECs was not reduced in AML PDX after AraC treatment (Figure 3D), suggesting that the abnormal vascular phenotype was maintained. This effect was not due to an unspecific toxicity of the chemotherapy on the vascular niche, as non-transplanted mice or mice engrafted with normal HSPCs and subjected to the same treatment did not show abnormal vascular permeability (Figures S3E and S3F), frequency, or number of ECs in the BM (Figures S3G and S3H). These results show that in AML disease, the vascular barrier functionality is still compromised after chemotherapeutic treatment. Furthermore, we observed that BM hypoxia was not normalized upon removal of leukemic cells (Figures 3E and 3F). Notably, similarly to what was observed in presence of high leukemic infiltration, most of the vessels were still close to hypoxic areas upon AraC treatment (Figure 3G). These data highlight a persistent poorly functional vasculature in the BM of AML PDX after chemotherapy.

AML-Induced Molecular Signature in Vascular ECs

AML cells have been extensively studied at the transcriptional level, with many reports assessing the production of angiogenic factors (Aref et al., 2002; Hatfield et al., 2009; Ribatti et al., 2007). To identify common downstream effectors of this angiogenic stimulation in the vascular niche, we studied the transcriptome of BM-derived ECs retrieved from mice engrafted with different AML patient-derived cells via RNA sequencing (RNA-seq), and compared it with ECs retrieved from non-engrafted mice or mice engrafted with normal HSPCs. As shown by the hierarchical clustering, despite the heterogeneity found among the groups, we identified a common deregulated signature associated with human AML engraftment (Figure 4A). Gene set enrichment analysis (GSEA) highlighted several altered processes directly associated with the abnormal vascular phenotype observed above, including vasculature development, angiogenesis, and response to hypoxia (Figure 4B). Several deregulated pathways associated with angiogenic and hypoxic stimulation were identified (Figures 4B and S4A). Interestingly, the expression of adhesion molecules changed dramatically upon AML engraftment, with upregulation of integrins associated with Fak pathway activation in ECs (Figure S4B) inducing migration, growth, and survival of newly forming vessels during angiogenesis (Muether et al., 2007; Stenzel et al., 2011; Weis and Cheresh, 2011), as well as initiation of VEGF-mediated vascular permeability (Chen et al., 2012; Eliceiri et al., 2002; Rodrigues and Granger, 2015). We noticed also reduced expression of tight junction components, e.g., *Cldn1* responsible for maintaining endothelial layer integrity (Rodrigues and Granger, 2015) or *Vcam1* required for HSC homing and retention to the niche (Lewandowski et al., 2010; Ulyanova et al., 2007). Interestingly, we found among the common highest upregulated genes in ECs upon AML engraftment *Nox4*, a NAPDH oxidase expressed in the vasculature and involved in the response to hypoxia via production of reactive oxygen species (ROS), activation of nitric oxide synthase 3 (NOS3), and release of nitric oxide (NO) (Craige et al., 2011) (Figure 4C). Of note, in response to AML engraftment, *Nox4* expression in ECs (Figure 4D) and ROS production in the BM (Figure 4E) were both increased. Moreover, the upregulated expression of *Nox4* in ECs and the high production of ROS in the BM were maintained after AraC treatment (Figures 4F and 4G). These data show that the AML-induced hypoxic environment alters the molecular signature of vascular ECs, activating several pro-angiogenic

n = 16. Data are shown as whiskers minimum-to-maximum plots, the line inside the box representing the mean, the top and the bottom line representing the 75% and 25% percentiles, respectively, and the lines above and below the box representing the SD.

(F) Representative 3D reconstruction of BM hypoxia imaged via intravital microscopy using the HypoxiSense probe together with vasculature (dextran) and Nestin$^+$ cells in mice transplanted with AML6 patient-derived cells and treated or not with AraC, as depicted. Scale bars represent 50 μm.

(G) Distribution and relative frequency of vessel distances to hypoxic areas in the BM of mice transplanted with AML6 patient-derived cells, and treated or not with AraC, as depicted.

ns, not significant; $^{**}p < 0.01$. See also Figure S3; Tables S1 and S2.

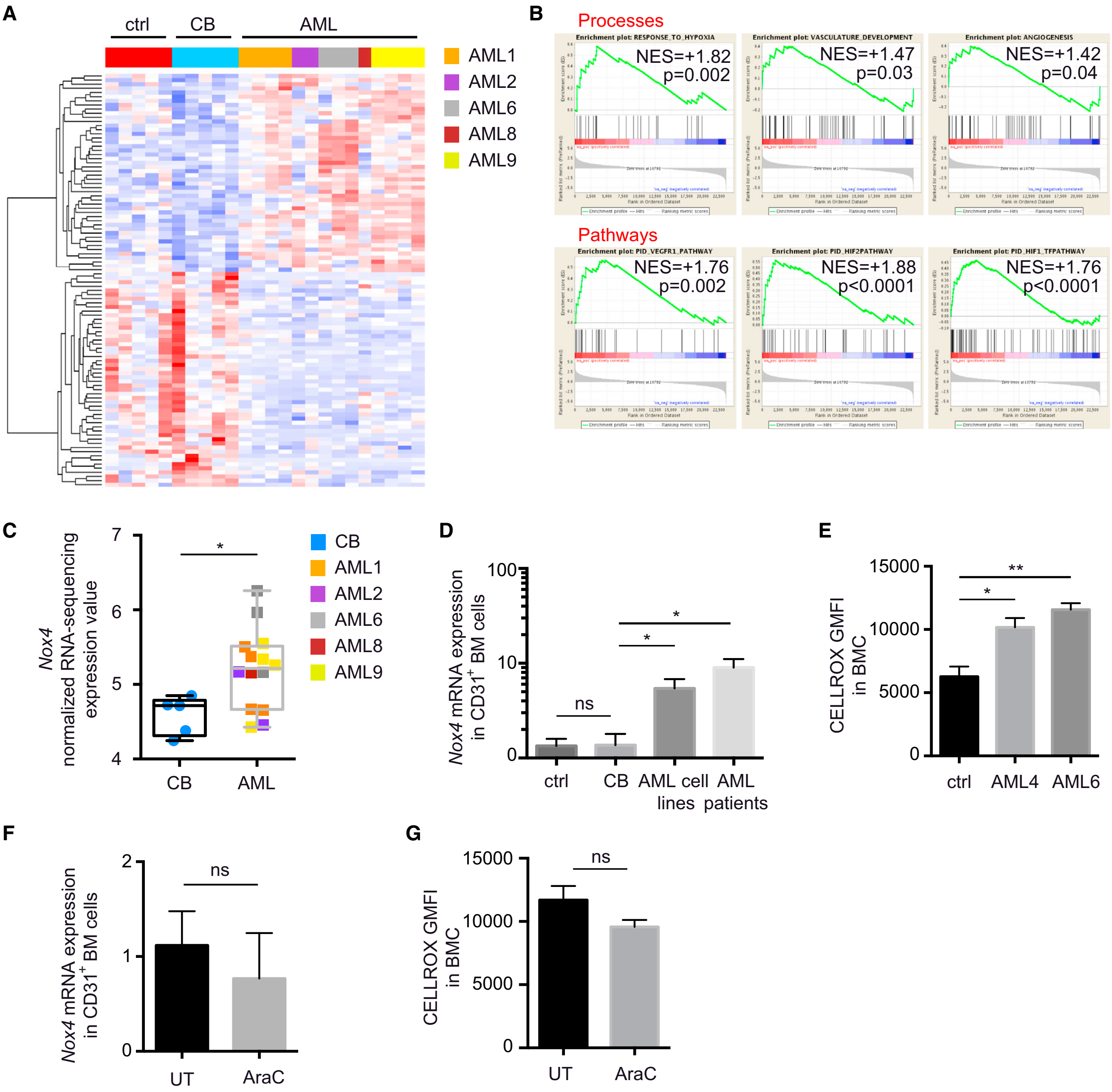

Figure 4. AML-Induced Transcriptional Signature in BM ECs

(A) Heatmap from hierarchical clustering of GSEA top 100 ranked genes between untransplanted mice (ctrl group), mice engrafted with human CB-derived HSPCs (CB group), and mice engrafted with different patient-derived samples (AML group) derived BM ECs, using RNA-seq data. Red indicates higher expression values and blue indicates lower expression values ($\log_2$ FPKM [fragments per kilobase per million mapped] scale).

(B) Enrichment plots for Processes (top) and Pathways (bottom) from GSEA between CB and AML groups using RNA-seq gene expression data indicate enrichment of these pathways in AML group. Normalized enrichment score (NES) and nominal p value are shown.

(C) Normalized expression values of *Nox4* in CB versus AML groups analyzed via RNA-seq. Data are shown as whiskers minimum-to-maximum plots, the line inside the box representing the mean, the top and the bottom lines representing the 75% and 25% percentiles, respectively, and the lines above and below the box representing the SD.

(D) *Nox4* expression analyzed by qRT-PCR in $CD31^+$ BM cells retrieved from the depicted groups of mice. CB, n = 5; AML cell lines, n = 8; AML patients (AML1, 2, 9), n = 13. Data are shown as mean ± SEM.

(E) Cellular ROS levels in BMC retrieved from mice of depicted groups; n = 3 per group. Data are shown as mean ± SEM.

(F) *Nox4* expression analyzed by qRT-PCR in $CD31^+$ BM cells retrieved from mice engrafted with AML6 and treated or not with AraC. n = 3 per group. Data are shown as mean ± SEM.

(G) Cellular ROS levels in BMC retrieved from mice engrafted with AML6 and treated or not with AraC; n = 3 per group. Data are shown as mean ± SEM.

ns, not significant; *p < 0.05, **p < 0.01. See also Figure S4.

pathways and positively regulating *Nox4* expression and the response to hypoxia.

AML Engraftment Is Associated with Increased Nitric Oxide Levels in the BM

As NO is the major mediator of vascular permeability in both physiological and several pathological conditions (Aramoto et al., 2004; Fukumura et al., 2001, 2006; Mayhan, 1999), we investigated whether the AML-derived upregulation of *Nox4* in ECs influenced NO production in the BM vascular niche. We found increased levels of NO in the BM of AML xenografts compared with non-transplanted mice or mice engrafted with normal HSPCs (Figure 5A). A slight but significant increase of NO levels was also observed in BM of PDX with low engraftment (not shown). This effect seemed to be specific to BM (Figure S5A). Of note, as already observed for vascular permeability and *Nox4* expression in ECs, chemotherapy failed to normalize the NO levels in the BM of AML PDX (Figure 5B).

To translate our observations to the human disease, we analyzed the NO levels in our cohort of AML patient-derived BM biopsies and compared them with those of healthy donors (Tables S1 and S2). We observed a significant increase in the NO level in BM aspirates of patients at diagnosis, which were similar to those observed in xenografts, compared with healthy donors (Figure 5C). NO levels were higher in the BM compared with the blood of the same patient at diagnosis (Figure S5B), similar to what was observed in mice with AML xenografts. Importantly, also similar to what was observed in AML xenografts, the NO levels remained elevated in the majority of post-treatment BM samples analyzed (Figures 5D and 5E). Moreover, failure in reducing NO levels appeared to be associated with a higher incidence of treatment failure (Figure S5C), with a probability of 73% in the cohort of patients studied herein (Figure S5D). This probability increases to 100% if we restrict the analysis to the intermediate-risk group samples, characterized by a more controversial prognostic assessment (Figure S5E). Our data suggest that persistence of high NO levels in the BM of patients after therapy could represent a biomarker for poor prognosis, particularly in patients with intermediate-risk AML. The upregulation of *Nox4* in ECs, indicating an overactivation of NOS3 in this compartment, likely is responsible for the increased NO levels in the BM. We therefore analyzed the expression of NOS3 in the two major components of the vascular niche, endothelial ($CD31^+$) and mesenchymal perivascular ($Nestin^+$) cells (Figure S5F). We observed that ECs represented the major compartment expressing NOS3 both at mRNA (Figure S5G) and protein (Figure 5F) levels. Moreover, we observed that NOS3 protein expression was significantly increased in ECs retrieved from mice engrafted with human AML patient-derived samples (Figure 5G). Of note, not only the total NOS3 protein level but also its activation was increased, as shown by increased phosphorylation of Ser1177 (Figure 5H (Kolluru et al., 2010)). In contrast, NOS3 was not increased in the mesenchymal compartment upon AML engraftment (Figure S5H). NOS2 protein expression is not changed in either the endothelial or the mesenchymal compartment upon AML engraftment (Figure S5I). Analysis of different endothelial subpopulations revealed that in normal BM, $CD31^+Sca\text{-}1^{high}$ ECs exhibit lower ROS and NO levels than $CD31^+Sca\text{-}1^{low}$ ECs (Figure 5I), and AML engraftment caused an increase in both ROS and NO levels in the $CD31^+$ $Sca\text{-}1^{high}$ population (Figure 5J). We investigated the consequences of long-term higher production of NO and ROS in ECs, and observed that in heavily engrafted AML xenografts ECs displayed increased apoptosis in both $Sca\text{-}1^{high}$ and $Sca\text{-}1^{low}$ ECs (Figure S5J), whereas cell-cycle arrest was restricted to the $Sca\text{-}1^{high}$ EC fraction (Figure S5K). We next evaluated whether the leukemic compartment might also contribute to the NO overproduction. Human AML cells do not express higher mRNA levels of *NOS2* or *NOS3* compared with normal hematopoietic cells residing in the BM, as reported in publicly available datasets (HemaExplorer/BloodSpot, not shown). Moreover, the mRNA levels of these enzymes in AML patient-derived samples were similar to those from healthy donors (Figures S5L and S5M). There was no significant upregulation of these enzymes after chemotherapy (Figures S5N and S5O), or in AML xenografts (P1) compared with their primary counterpart (P0) (Figure S5P). The NOS3 protein level in AML was much lower than that in the endothelial compartment (Figure S5Q). Our results show an overproduction of NO in the BM in the presence of AML at diagnosis as well as after therapy, produced by an abnormally activated NOS3 in BM ECs.

Targeting Vascular Permeability Cooperates with Chemotherapy to Improve AML Treatment Response

To test whether the exogenous production of NO has a role in leukemia transplantation and progression, we transplanted leukemic cells from two models of murine AML (MLL-AF9 and MLL-ENL) in *Nos3*-knockout (*Nos3*-KO) recipient mice and studied the status of the vasculature and disease progression. Interestingly, MLL-AF9 leukemia occurrence was significantly delayed in *Nos3*-KO mice (Figure S6A, top), *Nos3-KO* mice were active and healthy at the time wild-type (WT) mice showed clear signs of disease and were euthanized. Similar results were obtained using two distinct clones of MLL-ENL AML transplanted in WT or *Nos3*-KO recipient mice, showing significantly reduced disease penetrance (Figure S6A, bottom), reduced leukemic engraftment in the BM and spleen (Figures S6B and S6C), and amelioration of the anemic phenotype caused by AML (Figure S6D). Moreover, *Nos3* inactivation cooperated with AraC treatment to reduce leukemic engraftment in both MLL-AF9 and MLL-ENL models (Figure 6A) and reduced the vascular leakiness in the BM of AML-engrafted mice (Figure 6B). Together these data suggest that exogenous NO production by NOS3 has a role in AML engraftment. We next investigated whether the observed difference was likely to be attributed to a delay in engraftment or also to a reduction in disease progression. For that we used the PDX model and allowed AML cells to engraft the animals before inhibiting NO production with NOS inhibitors. We set up a similar treatment strategy to the one used before, including NOS inhibitors during and after the chemotherapy, to normalize the vascular permeability (Figure 6C). Treatment of mice xenografted with human ML1 cell line with NOS inhibitors in combination with chemotherapy significantly inhibited leukemic progression, as shown by reduced number of leukemic cells in the BM and the spleen (Figures S6E and S6F). The sole administration of NO inhibitors in heavily engrafted leukemic mice did not produce any significant decrease of leukemic load in the BM (Figure S6E), likely due to

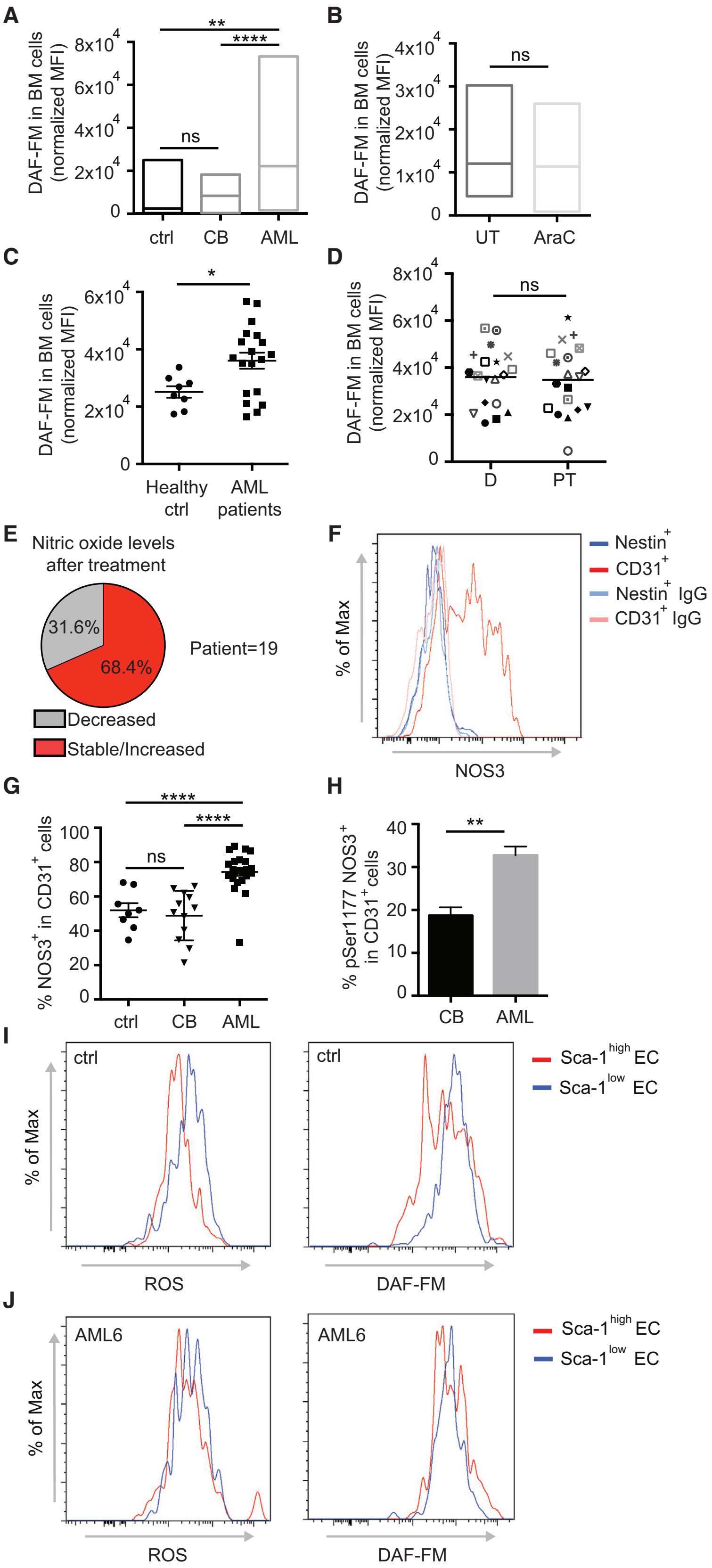

Figure 5. AML Engraftment Is Associated with Increased Nitric Oxide Levels in the BM

(A) Nitric oxide levels shown as DAF fluorescence intensity (normalized mean fluorescence intensity [MFI]) in BM cells of non-transplanted mice (ctrl) and mice transplanted with CB-derived HSPCs (CB) or AML patient-derived samples, as depicted. Ctrl, n = 13; CB, n = 24; AML patients (AML1, 2, 6, 8, 9), n = 35. Data are shown as minimum-to-maximum box plots, the line inside the box representing the mean.

(B) Nitric oxide levels (normalized MFI) in BM cells of mice transplanted with AML patient-derived cells, treated with solvent or AraC, as depicted. AML patients: AML6 and 9. UT, n = 7; AraC, n = 7. Data are shown as minimum-to-maximum box plots, the line inside the box representing the mean.

(C) Nitric oxide levels in BM cells of healthy donors or AML patients at diagnosis. Healthy ctrl, n = 8; AML patients, n = 19. Error bars represent mean ± SEM.

(D) Nitric oxide levels in BM cells of AML patients at diagnosis (D) and post treatment (PT); n = 18. Error bars represent mean.

(E) Pie chart showing the percentage of patients with decreased or stable/increased levels of nitric oxide after treatment.

(F) NOS3 expression measured via flow cytometry in Nestin$^+$ and CD31$^+$ cells, as depicted. Data are representative of triplicates.

(G) Percentage of NOS3$^+$ ECs measured by flow cytometry in the BM of non-transplanted mice and mice engrafted with CB-derived HSPCs (CB) or AML patient-derived samples, as depicted. Ctrl, n = 8; CB, n = 12; AML patients (AML2, 6, 8, 9), n = 24. Data are shown as mean ± SEM.

(H) Activated NOS3 (measured by flow cytometry with an anti-phosphoSer1177 antibody) in CD31$^+$ ECs of the BM of mice engrafted with CB-derived CD34$^+$ cells (CB) or AML patient-derived samples, as depicted. CB, n = 4; AML patients (AML2, 6, 9), n = 9. Data are shown as mean ± SEM.

(I) Cellular ROS (left) and nitric oxide (right) levels in different populations of BM-derived ECs, as depicted, in non-transplanted mice (ctrl). Data are representative of triplicates.

(J) Cellular ROS (left) and nitric oxide (right) levels in different populations of BM-derived ECs, as depicted, in mice engrafted with AML6 patient-derived cells. Data are representative of triplicates.

ns, not significant; *$p < 0.05$, **$p < 0.01$, ****$p < 0.0001$. See also Figure S5.

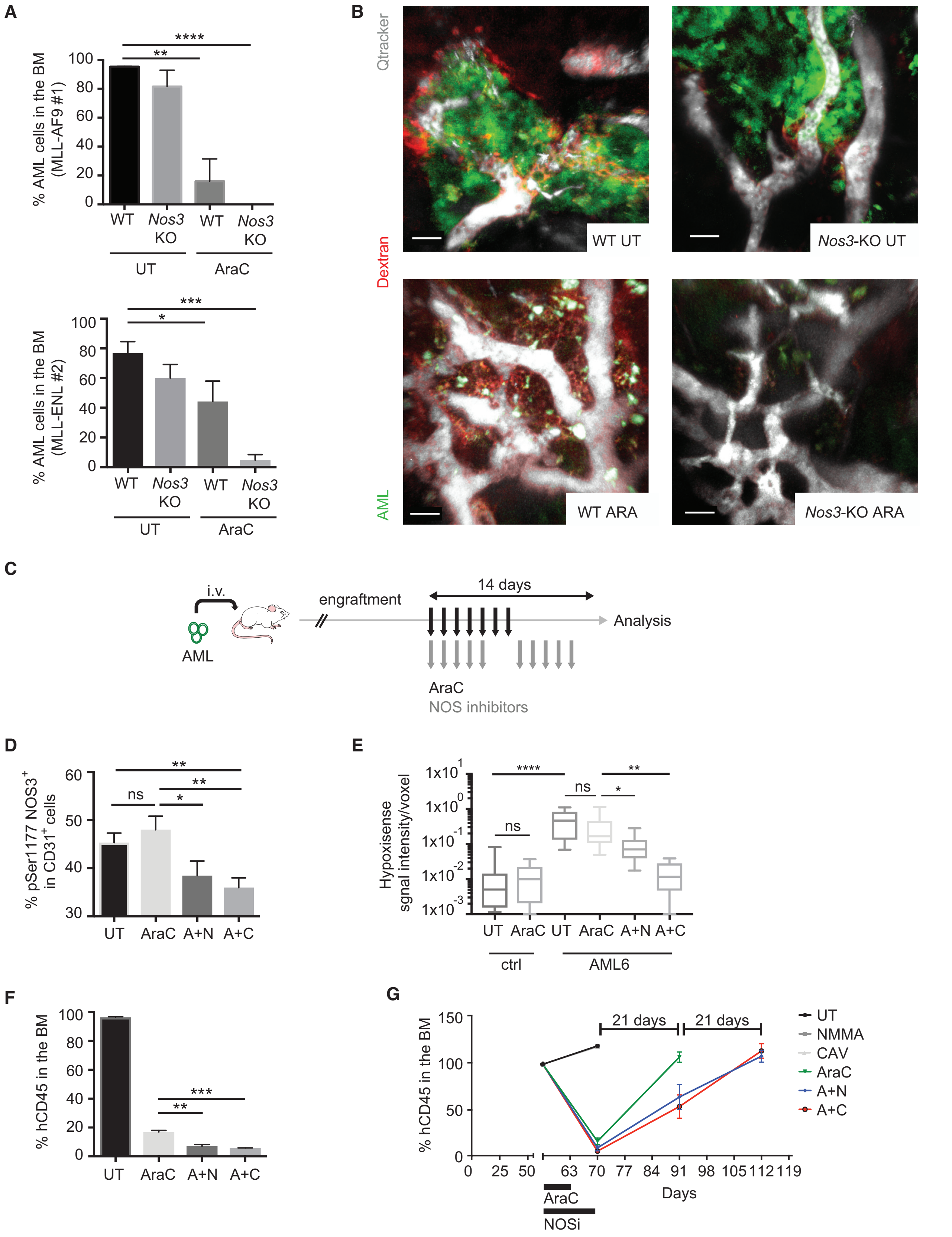

(legend on next page)

the absence of a specific effect of NO on leukemic cells. Indeed, treatment of leukemic cells (ML1 or primary patient samples) *in vitro* with NO inhibitor or NO donor did not affect their survival or proliferation ability (Figures S6G and S6H). NO inhibition combined with AraC treatment reduced NOS3 activation (Figure 6D), vascular leakiness (Figure S6I), and the BM hypoxia (Figure 6E). Importantly, the combined treatment was more powerful than the chemotherapy alone in reducing leukemic progression in the BM (Figures 6F and S6J) and the spleen (Figure S6K) of mice engrafted with two different patient-derived samples (Figure S6L). The combined treatment also significantly extended the ''remission-like'' phase of the disease compared with chemotherapy alone (Figure 6G). These data show that increased NO levels contribute to the vascular leakiness in AML-engrafted mice, and restoring normal vascular function in the BM by NOS inhibition improves the therapeutic response compared with standard chemotherapy.

NOS Inhibition Potentiates Normal HSPC Function

Increased levels of NO have been associated with increased HSPC motility and egress from the BM, leading to a lower repopulation capacity of the BM stem cell pool after injuries (Aicher et al., 2003; Gur-Cohen et al., 2015). Moreover, recent findings indicate that loss of vascular integrity promotes HSPC mobilization and loss of the HSC pool in the BM (Itkin et al., 2016). We thus speculated that increased NO and vascular leakiness in AML could affect normal HSC. In the BM of AML xenografts, the residual murine HSC displayed higher levels of ROS (Figure 7A) and were mobilized to the periphery, as shown by increased SLAM$^+$ cells in the blood of mice with AML xenografts compared with those without, as well as a reduced proportion of HSC in the BM (Figures 7B and 7C). The absolute number of SLAM$^+$ cells in the BM was also decreased in mice with AML xenografts and in mice transplanted with murine MLL-ENL leukemic cells (Figures S7A and S7B). Therefore, we tested whether normalization of the vascular niche with NOS inhibitors would be beneficial for normal HSC function. We observed that NOS inhibitors in combination with AraC treatment were more efficient than chemotherapy alone in re-establishing the normal number of SLAM$^+$ cells in the BM of PDX (Figure 7D). Not only the number but also the stem cell function of these SLAM$^+$ cells was increased, as shown by their repopulation ability in secondary recipients (Figures S7C and 7E–7G). These data suggest that overproduction of NO by altered vascular niche affects normal residual HSC in the BM and that NOS inhibition may improve AML therapy by restoring normal stem cell function. We next tested the effect of NOS inhibitors on HSPCs. Hence, we treated mice engrafted with normal HSPCs with NOS inhibitors and analyzed the effect on the stem cell pool (Figure 7H). Interestingly, we observed reduced HSPC egress to the blood (Figure 7I), an effect specific for this compartment as no significant difference was observed on the egress of total hCD45$^+$ cells into the blood (Figure S7D). This was accompanied by a specific increase of HSPCs in the BM (Figures 7J and S7E). This effect was not due to an increase in proliferation of the stem pool, as shown by cell-cycle analysis (Figure S7F), thus excluding a possible exhaustion of the compartment. Indeed, NO inhibition not only increased the number of HSPCs in the BM but also their stem cell function, as shown by their increased repopulation ability in secondary transplantation assays (Figure 7K). We next tested whether NO inhibition would be beneficial for residual normal HSPC to outcompete AML cells during the relapse process. To achieve this, and mimic the competition between AML cells and normal HSCs into the BM niche, we injected simultaneously normal human CB-derived HSPCs and AML patient-derived cells intravenously into non-irradiated NBSGW mice (McIntosh et al., 2015) and monitored the normal versus malignant engraftment in the presence or absence of long-term NOS inhibitors (Figure 7L). Analysis of the BM engraftment at the end of the treatment showed that NOS inhibition favored the normal over leukemic engraftment in the BM (Figures 7M and 7N). Together, these results suggest that the use of NO inhibitors will not only reverse the vascular permeability observed during AML progression and thus improve chemotherapy efficiency, but will also restore a normal stem cell vascular niche, likely protecting normal residual HSCs and allowing them to outcompete the leukemic cells.

Altogether, our results provide strong evidence for the importance of the alteration of the vascular niche in AML progression and relapse, via increased NO production by ECs, and call for clinical trials incorporating NOS inhibitors to target the abnormal vascular niche and improve the treatment response.

Figure 6. Targeting Vascular Permeability Cooperates with Chemotherapy to Improve AML Treatment

(A) Percentage of murine GFP$^+$ MLL-AF9 #1 or tomato$^+$ MLL-ENL #2 AML cells in the BM of secondary mice of depicted genotypes, treated with solvent or AraC; n = 3 or more replicates. Data are shown as mean ± SEM.

(B) Representative 3D reconstruction of calvarium BM of mice of depicted genotypes transplanted with murine MLL-AF9 #1 AML cells, treated with solvent or AraC. Scale bars represent 30 μm.

(C) Schematic of the experiment. Mice engrafted with human AML patients' samples were treated with AraC or solvent for 1 week, and with or without NOS inhibitors for 2 weeks.

(D) Activated NOS3 protein (measured by flow cytometry with an anti-phosphoSer1177 antibody) in CD31$^+$ endothelial cells of the BM of mice transplanted with AML6 and AML9 patient-derived cells, treated as described in (C); n = 6. Data are shown as mean ± SEM.

(E) Hypoxia represented as HypoxiSense signal intensity/voxel in the calvaria BM of non-transplanted mice (ctrl) and mice transplanted with AML6 patient-derived samples, treated or not with AraC, as depicted. Ctrl UT, n = 13; ctrl AraC, n = 10; AML6 UT, n = 18; AML6 AraC, n = 20; AML6 A + N, n = 9; AML6 A + C, n = 9. Data are shown as whiskers minimum-to-maximum plots, the line inside the box representing the mean, the top and the bottom lines representing the 75% and 25% percentiles, respectively, and the lines above and below the box representing the SD.

(F) Percentage of human CD45$^+$ engraftment in the BM of mice engrafted with patient-derived sample AML6 and treated as in (C); n = 3 per condition. Data are shown as mean ± SEM.

(G) Percentage of human CD45$^+$ engraftment measured via BM aspirate in mice engrafted with AML 6 and treated as in (C). Data are shown as mean ± SEM.

ns, not significant; *p < 0.05, **p < 0.01, ***p < 0.001, ****p < 0.0001. See also Figure S6.

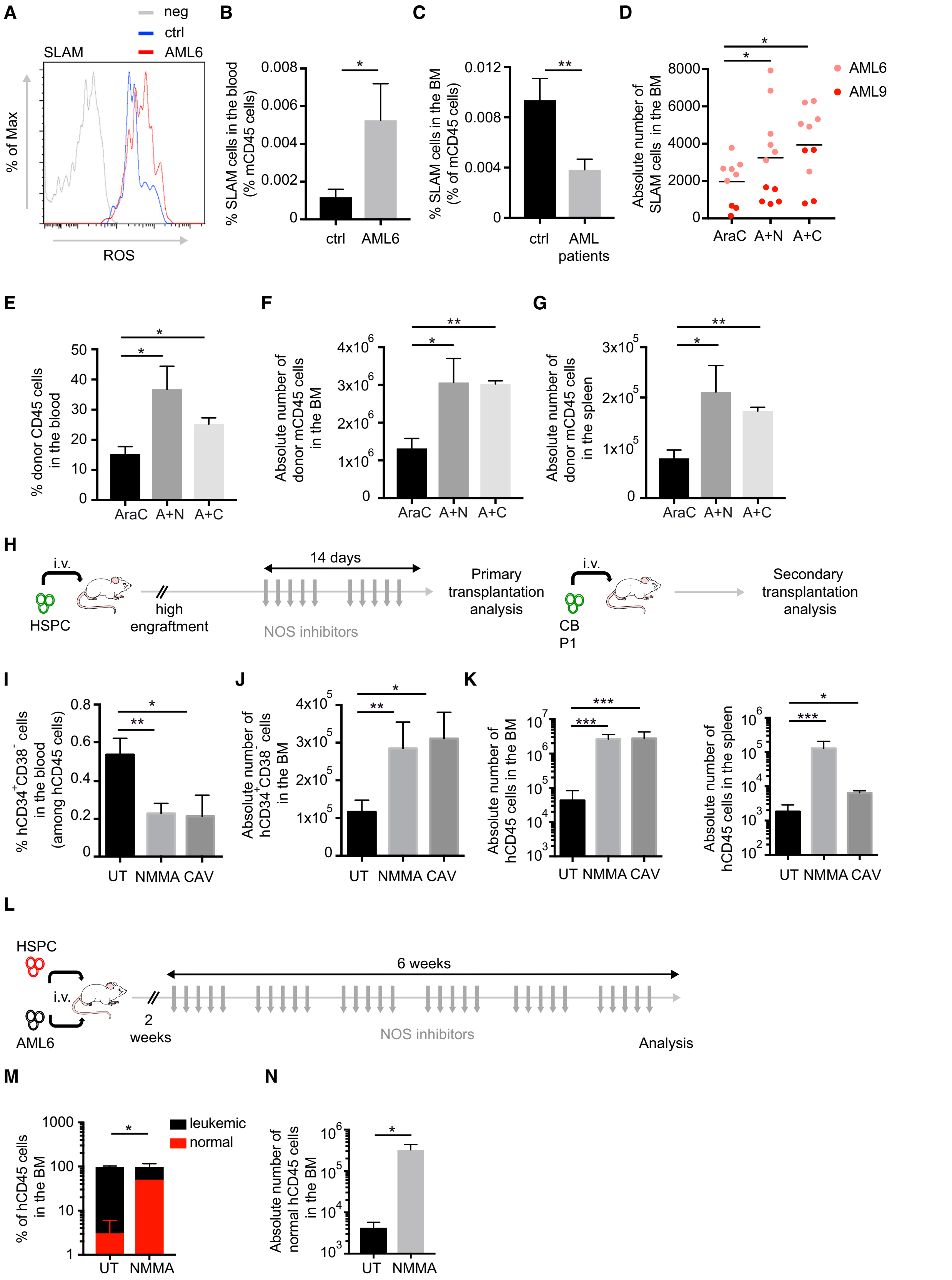

(legend on next page)

DISCUSSION

Tumor angiogenesis is a pathological complication of several types of solid cancers, which has been extensively studied and characterized. The angiogenic process is mostly initiated by pro-angiogenic factors derived from tumor cells, which in turn activates a pro-inflammatory response in the microenvironment, associated with the initiation of irregular and uncontrolled vascular growth. Newly formed tumor vessels appear abnormal and leaky in most of the solid cancers. This structurally aberrant network affects the local blood flow, metabolite exchange, and oxygenation, leading to poor drug delivery, increased tumor growth, and metastatic potential (Carmeliet and Jain, 2011a, 2011b; Jain, 2005).

In the AML context, leukemic cells have been shown to modify ECs *ex vivo* via several pathways, mainly mediated by pro-angiogenic factors such as VEGF family members (Kampen et al., 2013; Poulos et al., 2014). Moreover, publicly available datasets as well as several studies reported that VEGF is not the only pro-angiogenic factor produced by AML cells, which may be a major reason why clinical trials incorporating VEGF inhibitors have not been very successful. AML cells also express high levels of hepatocyte growth factor (Aref et al., 2002), fibroblast growth factor (Ribatti et al., 2007), and other factors (Chand et al., 2016; Hatfield et al., 2009; Lee and Kim, 2014). However, no extensive analysis of the AML vascular niche *in vivo* has been reported.

In the present study, we provide a detailed picture of the vasculature in AML. We not only confirm that AML engraftment induces hypoxia and angiogenesis in the BM but also show that it alters the normal vascular architecture and function. ECs involved in the angiogenic process are highly metabolically active and consume a high level of oxygen (Potente and Carmeliet, 2017). As a result, these newly formed abnormal vessels are incapable of supplying nutrients and oxygen (as well as drugs) in a homogeneous way in the BM tissue, likely contributing to maintain the overall hypoxia. At a late stage of disease, after facing an exponential and uncontrolled growth and being exposed to high levels of NO/ROS production, ECs display an increased apoptotic phenotype, likely associated with a toxic process of vascular regression (Lobov et al., 2011).

The crosstalk between leukemic cells and ECs in the BM has not been extensively studied at the molecular level. The available data in the literature rely on the *in vitro* co-culture system, which does not faithfully reproduce the complexity of the *in vivo* environment. Our analysis of the transcriptome of vascular ECs upon human AML engraftment provides important insights into the field. First, it confirms at the molecular level the observed toxic phenotypes. Second, it highlights commonly deregulated genes and pathways, which can represent potential targets for the disruption of the AML-microenvironment crosstalk. Interestingly, the NOX4-NOS3 axis, already reported previously to act in response to stress induced by hypoxic conditions (Craige et al., 2011; Drummond et al., 2000; Mittal et al., 2007; Thomas et al., 2002; Zhang et al., 2008), has a major role in orchestrating the AML-induced vascular leakiness.

Persistent vascular leakiness and increased NO levels in AML xenografts after induction therapy suggest that the abnormal and poorly functional vessels participate to maintain a hypoxic vascular environment during the remission phase, likely contributing to treatment failure. Our hypothesis is strengthened by the analysis of NO in an independent cohort of patients which, although relatively small, faithfully represents the high diversity of the AML genetic landscape. Our analysis argues for NO as a potential prognostic BM marker, as stable/increased levels of NO post-induction therapy seems to be associated with a poor clinical outcome.

Increased vascular leakiness can lead to poor drug delivery and to a microenvironment favoring resistance to therapy and

Figure 7. Targeting Vascular Permeability Potentiates HSPC Function

(A) Cellular ROS levels in BM-derived HSC of non-transplanted mice or mice engrafted with AML6 patient-derived cells. Data are representative of triplicates.

(B) Percentage of murine HSC among total murine $CD45^+$ cells in the blood of non-transplanted mice and mice engrafted with AML6 patient-derived cells. ctrl, n = 5; AML6, n = 3. Data are shown as mean ± SEM.

(C) Percentage of murine HSC among total murine $CD45^+$ cells in the BM of non-transplanted mice of mice engrafted with AML patient-derived samples (AML6, 9). ctrl, n = 5; AML patients, n = 12. Data are shown as mean ± SEM.

(D) Absolute number of murine HSC in the BM of mice transplanted with AML patient-derived cells, as depicted, and treated with AraC alone or in combination with NOS inhibitors. Each dot represents a mouse. Error bars represent mean.

(E–G) Percentage in blood (E) and absolute number in BM (F) and spleen (G) of donor CD45.1, retrieved from NSG mice transplanted with AML6 patient-derived cells and treated with AraC alone or in combination with NOS inhibitors, injected in secondary CD45.2 *Rag2*-KO/*Gammac*-KO mice. AraC, n = 4; A + N, n = 3; A + C, n = 3. Data are shown as mean ± SEM.

(H) Schematic of the experiment. Mice engrafted with human $CD34^+$ CB cells were treated with NOS inhibitors (N^G-monomethyl-L-arginine [NMMA] 20 mg/kg/day intraperitoneally or cavtratin 2 mg/kg/day intraperitoneally) for 2 weeks, 5 days/week. Mice were euthanized and BMC harvested for subsequent analysis and secondary transplantation.

(I) Percentage of HSPCs in the peripheral blood of mice engrafted with CB-derived HSPCs and treated or not with NMMA or cavtratin. Data are shown as mean ± SEM.

(J) Absolute number of HSPC in the BM of mice engrafted with CB-derived HSPC cells and treated or not with NMMA or cavtratin. Data are shown as mean ± SEM.

(K) Absolute number of human $CD45^+$ cells in the BM (left) and spleen (right) of secondary NSG mice transplanted with primary xenografts described in (H). Data are shown as mean ± SEM.

(L) Schematic of the experiment. Non-irradiated NBSGW mice were simultaneously transplanted with $tomato^+$ HSPCs (red) and non-transduced AML6 (black) patient-derived cells, and 2 weeks after transplantation control solvent or NMMA were administered for 6 weeks, 5 days/week. Two days after the last treatment, mice were euthanized and BM was analyzed.

(M) Percentage of $tomato^+$ (normal, red) and $tomato^-$ (leukemic, black) cells among $hCD45^+$ in the BM of mice described in (L); n = 4. Data are shown as mean ± SEM.

(N) Absolute number of $tomato^+$ $hCD45^+$ (normal) cells in the BM of mice described in (L). Data are shown as mean ± SEM.

ns, not significant; *p < 0.05, **p < 0.01, ***p < 0.001. See also Figure S7.

relapse. The participation of vascular leakiness to the pathophysiology of AML can be exerted at different levels. First, increased vascular permeability deregulates the balance of blood supply to the BM and, consequently, drug delivery. This leads to the formation of areas with low perfusion rate, where leukemic cells can hide and escape therapy. Restoring a normal vascular function would allow more homogeneous chemotherapeutic delivery. Second, high vascular permeability (Itkin et al., 2016) and high NO levels (Gur-Cohen et al., 2015) have recently been associated with increased HSC activation and egress from the BM in mice, with a consequent reduction of their repopulating activity. Our data showing increased ROS levels and higher egress of BM-derived HSCs are in line with these findings, further extending them to the human HSPC compartment. Indeed, we show that inhibition of NO results in an enriched number and activity of BM HSPCs. Conversely, the presence of AML engraftment induces the generation of an exogenous overproduction of NO in the BM, which likely affects the HSC motility and activity, bringing a rapid exhaustion of their functionality. In this scenario, inhibition of NO would produce multiple benefits to AML patients. NOS inhibitors are already in clinical trials (Granados-Principal et al., 2015; Ozkor et al., 2015) and specific inhibitors of NOS3 have been developed (Bucci et al., 2000; Gratton et al., 2003). Thus, our preclinical data using both genetic and pharmacologic approaches to inhibit NO in combination with AraC treatment show that targeting vascular permeability would be a successful strategy to normalize the BM vascular functionality, improve therapeutic responses, and preserve normal HSC function.

STAR★METHODS

Detailed methods are provided in the online version of this paper and include the following:

- KEY RESOURCES TABLE
- CONTACT FOR REAGENT AND RESOURCE SHARING
  - Human Primary Samples
  - Mouse Models
  - Drug Treatments *In Vivo*
  - Intravital Two-Photon Microscopy
  - Images Processing
  - Bioluminescence Imaging
  - Cell Lines
  - Transduction and Culture of Human Primary Samples
  - Transduction and Culture of Murine Primary Samples
  - AML and HSPCs Transplantation Assay *In Vivo*
  - Bone Marrow Cell Isolation
  - HO Diffusion
  - Hypoxiprobe Assessment
  - Flow Cytometry Analysis and Cell Sorting
  - Drug Treatments *In Vitro*
  - Reverse Transcriptase and Real Time Quantitative PCR (RT-qPCR)
  - Histological Processing and Immunostaining
  - Sample Preparation for RNA-Sequencing
  - RNA-Sequencing Analysis Methods
  - Statistical Analysis
- DATA AND SOFTWARE AVAILABILITY
  - Data Resources

SUPPLEMENTAL INFORMATION

Supplemental Information includes seven figures and two tables and can be found with this article online at http://dx.doi.org/10.1016/j.ccell.2017.08.001.

AUTHOR CONTRIBUTIONS

D.P. designed, conducted, and performed the experiments and wrote the manuscript. A.d.T., A.A., K.R.-P., K.F., F.L., L.A.M., and B.M. performed some experiments. L.B. prepared the libraries and P.C. performed the statistical analysis of RNA-seq data. G.D. performed statistical analysis on patients' cohorts. J.G. provided patient samples and clinical information. D.B. designed experiments and wrote the manuscript.

ACKNOWLEDGMENTS

This work is dedicated to Dr François Lasailly. The authors would like to thank members of the Biological Resource Unit, Flow Cytometry, *In Vivo* Imaging, Computational Biology, Advanced Sequencing and Light Microscopy core facilities at the Francis Crick Institute for their valuable help. The authors are grateful to Dr. W. Gray for critical reading of the manuscript, Dr. F. Anjos-Afonso for critical discussions and technical support, and Prof. A. Fielding and Dr. R. Burt for providing some human healthy control BM. We would like to thank Dr. O. William for providing us with MLL-AF9 and MLL-ENL mouse leukemias. We would like to thank Mrs. Shohreh Beski (Obstetric Department, Royal London Hospital, London, UK) and her team as well as Mrs. Jashu Patel for helping in the collection of umbilical cord blood. We are grateful to the ASH-EHA TRTH joint program for the valuable help in supporting and improving the project. D.P. was supported by a non-clinical research fellowship from EHA. This work was supported by The Francis Crick Institute, which receives its core funding from Cancer Research UK (FC001045), The UK Medical Research Council (FC001045), and the Welcome Trust (FC001045).

Received: October 7, 2016
Revised: April 25, 2017
Accepted: August 1, 2017
Published: August 31, 2017

REFERENCES

Aicher, A., Heeschen, C., Mildner-Rihm, C., Urbich, C., Ihling, C., Technau-Ihling, K., Zeiher, A.M., and Dimmeler, S. (2003). Essential role of endothelial nitric oxide synthase for mobilization of stem and progenitor cells. Nat. Med. *9*, 1370–1376.

Aramoto, H., Breslin, J.W., Pappas, P.J., Hobson, R.W., 2nd, and Duran, W.N. (2004). Vascular endothelial growth factor stimulates differential signaling pathways in in vivo microcirculation. Am. J. Physiol. Heart Circ. Physiol. *287*, H1590–H1598.

Aref, S., Mabed, M., Sakrana, M., Goda, T., and El-Sherbiny, M. (2002). Soluble hepatocyte growth factor (sHGF) and vascular endothelial growth factor (sVEGF) in adult acute myeloid leukemia: relationship to disease characteristics. Hematology *7*, 273–279.

Aref, S., El Sherbiny, M., Goda, T., Fouda, M., Al Askalany, H., and Abdalla, D. (2005). Soluble VEGF/sFLt1 ratio is an independent predictor of AML patient outcome. Hematology *10*, 131–134.

Bao, B., Groves, K., Zhang, J., Handy, E., Kennedy, P., Cuneo, G., Supuran, C.T., Yared, W., Rajopadhye, M., and Peterson, J.D. (2012). In vivo imaging and quantification of carbonic anhydrase IX expression as an endogenous biomarker of tumor hypoxia. PLoS One *7*, e50860.

Boulais, P.E., and Frenette, P.S. (2015). Making sense of hematopoietic stem cell niches. Blood *125*, 2621–2629.

Bucci, M., Gratton, J.P., Rudic, R.D., Acevedo, L., Roviezzo, F., Cirino, G., and Sessa, W.C. (2000). In vivo delivery of the caveolin-1 scaffolding domain inhibits nitric oxide synthesis and reduces inflammation. Nat. Med. *6*, 1362–1367.

Buchner, T., Schlenk, R.F., Schaich, M., Dohner, K., Krahl, R., Krauter, J., Heil, G., Krug, U., Sauerland, M.C., Heinecke, A., et al. (2012). Acute myeloid leukemia (AML): different treatment strategies versus a common standard arm–combined prospective analysis by the German AML Intergroup. J. Clin. Oncol. *30*, 3604–3610.

Carmeliet, P., and Jain, R.K. (2011a). Molecular mechanisms and clinical applications of angiogenesis. Nature *473*, 298–307.

Carmeliet, P., and Jain, R.K. (2011b). Principles and mechanisms of vessel normalization for cancer and other angiogenic diseases. Nat. Rev. Drug Discov. *10*, 417–427.

Chand, R., Chandra, H., Chandra, S., and Verma, S.K. (2016). Role of microvessel density and vascular endothelial growth factor in angiogenesis of hematological malignancies. Bone Marrow Res. *2016*, 5043483.

Chen, X.L., Nam, J.O., Jean, C., Lawson, C., Walsh, C.T., Goka, E., Lim, S.T., Tomar, A., Tancioni, I., Uryu, S., et al. (2012). VEGF-induced vascular permeability is mediated by FAK. Dev. Cell *22*, 146–157.

Colmone, A., Amorim, M., Pontier, A.L., Wang, S., Jablonski, E., and Sipkins, D.A. (2008). Leukemic cells create bone marrow niches that disrupt the behavior of normal hematopoietic progenitor cells. Science *322*, 1861–1865.

Craige, S.M., Chen, K., Pei, Y., Li, C., Huang, X., Chen, C., Shibata, R., Sato, K., Walsh, K., and Keaney, J.F., Jr. (2011). NADPH oxidase 4 promotes endothelial angiogenesis through endothelial nitric oxide synthase activation. Circulation *124*, 731–740.

Dobin, A., Davis, C.A., Schlesinger, F., Drenkow, J., Zaleski, C., Jha, S., Batut, P., Chaisson, M., and Gingeras, T.R. (2013). STAR: ultrafast universal RNA-seq aligner. Bioinformatics *29*, 15–21.

Dong, L., Yu, W.M., Zheng, H., Loh, M.L., Bunting, S.T., Pauly, M., Huang, G., Zhou, M., Broxmeyer, H.E., Scadden, D.T., and Qu, C.K. (2016). Leukaemogenic effects of Ptpn11 activating mutations in the stem cell microenvironment. Nature *539*, 304–308.

Drummond, G.R., Cai, H., Davis, M.E., Ramasamy, S., and Harrison, D.G. (2000). Transcriptional and posttranscriptional regulation of endothelial nitric oxide synthase expression by hydrogen peroxide. Circ. Res. *86*, 347–354.

Egawa, G., Nakamizo, S., Natsuaki, Y., Doi, H., Miyachi, Y., and Kabashima, K. (2013). Intravital analysis of vascular permeability in mice using two-photon microscopy. Sci. Rep. *3*, 1932.

Eliceiri, B.P., Puente, X.S., Hood, J.D., Stupack, D.G., Schlaepfer, D.D., Huang, X.Z., Sheppard, D., and Cheresh, D.A. (2002). Src-mediated coupling of focal adhesion kinase to integrin alpha(v)beta5 in vascular endothelial growth factor signaling. J. Cell Biol. *157*, 149–160.

Fiedler, W., Mesters, R., Tinnefeld, H., Loges, S., Staib, P., Duhrsen, U., Flasshove, M., Ottmann, O.G., Jung, W., Cavalli, F., et al. (2003). A phase 2 clinical study of SU5416 in patients with refractory acute myeloid leukemia. Blood *102*, 2763–2767.

Fiedler, W., Serve, H., Dohner, H., Schwittay, M., Ottmann, O.G., O'Farrell, A.M., Bello, C.L., Allred, R., Manning, W.C., Cherrington, J.M., et al. (2005). A phase 1 study of SU11248 in the treatment of patients with refractory or resistant acute myeloid leukemia (AML) or not amenable to conventional therapy for the disease. Blood *105*, 986–993.

Foster, K., Lassailly, F., Anjos-Afonso, F., Currie, E., Rouault-Pierre, K., and Bonnet, D. (2015). Different motile behaviors of human hematopoietic stem versus progenitor cells at the osteoblastic niche. Stem Cell Rep. *5*, 690–701.

Frisch, B.J., Ashton, J.M., Xing, L., Becker, M.W., Jordan, C.T., and Calvi, L.M. (2012). Functional inhibition of osteoblastic cells in an in vivo mouse model of myeloid leukemia. Blood *119*, 540–550.

Fukumura, D., and Jain, R.K. (2008). Imaging angiogenesis and the microenvironment. APMIS *116*, 695–715.

Fukumura, D., Gohongi, T., Kadambi, A., Izumi, Y., Ang, J., Yun, C.O., Buerk, D.G., Huang, P.L., and Jain, R.K. (2001). Predominant role of endothelial nitric oxide synthase in vascular endothelial growth factor-induced angiogenesis and vascular permeability. Proc. Natl. Acad. Sci. USA *98*, 2604–2609.

Fukumura, D., Kashiwagi, S., and Jain, R.K. (2006). The role of nitric oxide in tumour progression. Nat. Rev. Cancer *6*, 521–534.

Giles, F.J., Bellamy, W.T., Estrov, Z., O'Brien, S.M., Verstovsek, S., Ravandi, F., Beran, M., Bycott, P., Pithavala, Y., Steinfeldt, H., et al. (2006). The anti-angiogenesis agent, AG-013736, has minimal activity in elderly patients with poor prognosis acute myeloid leukemia (AML) or myelodysplastic syndrome (MDS). Leuk. Res. *30*, 801–811.

Granados-Principal, S., Liu, Y., Guevara, M.L., Blanco, E., Choi, D.S., Qian, W., Patel, T., Rodriguez, A.A., Cusimano, J., Weiss, H.L., et al. (2015). Inhibition of iNOS as a novel effective targeted therapy against triple-negative breast cancer. Breast Cancer Res. *17*, 25.

Gratton, J.P., Lin, M.I., Yu, J., Weiss, E.D., Jiang, Z.L., Fairchild, T.A., Iwakiri, Y., Groszmann, R., Claffey, K.P., Cheng, Y.C., and Sessa, W.C. (2003). Selective inhibition of tumor microvascular permeability by cavtratin blocks tumor progression in mice. Cancer Cell *4*, 31–39.

Grimwade, D., Hills, R.K., Moorman, A.V., Walker, H., Chatters, S., Goldstone, A.H., Wheatley, K., Harrison, C.J., and Burnett, A.K.; National Cancer Research Institute Adult Leukaemia Working Group (2010). Refinement of cytogenetic classification in acute myeloid leukemia: determination of prognostic significance of rare recurring chromosomal abnormalities among 5876 younger adult patients treated in the United Kingdom Medical Research Council trials. Blood *116*, 354–365.

Gur-Cohen, S., Itkin, T., Chakrabarty, S., Graf, C., Kollet, O., Ludin, A., Golan, K., Kalinkovich, A., Ledergor, G., Wong, E., et al. (2015). PAR1 signaling regulates the retention and recruitment of EPCR-expressing bone marrow hematopoietic stem cells. Nat. Med. *21*, 1307–1317.

Hanoun, M., Zhang, D., Mizoguchi, T., Pinho, S., Pierce, H., Kunisaki, Y., Lacombe, J., Armstrong, S.A., Duhrsen, U., and Frenette, P.S. (2014). Acute myelogenous leukemia-induced sympathetic neuropathy promotes malignancy in an altered hematopoietic stem cell niche. Cell Stem Cell *15*, 365–375.

Hatfield, K., Oyan, A.M., Ersvaer, E., Kalland, K.H., Lassalle, P., Gjertsen, B.T., and Bruserud, O. (2009). Primary human acute myeloid leukaemia cells increase the proliferation of microvascular endothelial cells through the release of soluble mediators. Br. J. Haematol. *144*, 53–68.

Hooper, A.T., Butler, J.M., Nolan, D.J., Kranz, A., Iida, K., Kobayashi, M., Kopp, H.G., Shido, K., Petit, I., Yanger, K., et al. (2009). Engraftment and reconstitution of hematopoiesis is dependent on VEGFR2-mediated regeneration of sinusoidal endothelial cells. Cell Stem Cell *4*, 263–274.

Horton, S.J., Grier, D.G., McGonigle, G.J., Thompson, A., Morrow, M., De Silva, I., Moulding, D.A., Kioussis, D., Lappin, T.R., Brady, H.J., and Williams, O. (2005). Continuous MLL-ENL expression is necessary to establish a "Hox Code" and maintain immortalization of hematopoietic progenitor cells. Cancer Res. *65*, 9245–9252.

Horton, S.J., Walf-Vorderwulbecke, V., Chatters, S.J., Sebire, N.J., de Boer, J., and Williams, O. (2009). Acute myeloid leukemia induced by MLL-ENL is cured by oncogene ablation despite acquisition of complex genetic abnormalities. Blood *113*, 4922–4929.

Hussong, J.W., Rodgers, G.M., and Shami, P.J. (2000). Evidence of increased angiogenesis in patients with acute myeloid leukemia. Blood *95*, 309–313.

Itkin, T., Gur-Cohen, S., Spencer, J.A., Schajnovitz, A., Ramasamy, S.K., Kusumbe, A.P., Ledergor, G., Jung, Y., Milo, I., Poulos, M.G., et al. (2016). Distinct bone marrow blood vessels differentially regulate haematopoiesis. Nature *532*, 323–328.

Jain, R.K. (2005). Normalization of tumor vasculature: an emerging concept in antiangiogenic therapy. Science *307*, 58–62.

Kampen, K.R., Ter Elst, A., and de Bont, E.S. (2013). Vascular endothelial growth factor signaling in acute myeloid leukemia. Cell Mol. Life Sci. *70*, 1307–1317.

Karolchik, D., Hinrichs, A.S., Furey, T.S., Roskin, K.M., Sugnet, C.W., Haussler, D., and Kent, W.J. (2004). The UCSC Table Browser data retrieval tool. Nucleic Acids Res. *32*, D493–D496.

Kim, Y.W., Koo, B.K., Jeong, H.W., Yoon, M.J., Song, R., Shin, J., Jeong, D.C., Kim, S.H., and Kong, Y.Y. (2008). Defective Notch activation in microenvironment leads to myeloproliferative disease. Blood *112*, 4628–4638.

Kode, A., Manavalan, J.S., Mosialou, I., Bhagat, G., Rathinam, C.V., Luo, N., Khiabanian, H., Lee, A., Murty, V.V., Friedman, R., et al. (2014). Leukaemogenesis induced by an activating beta-catenin mutation in osteoblasts. Nature *506*, 240–244.

Kolluru, G.K., Siamwala, J.H., and Chatterjee, S. (2010). eNOS phosphorylation in health and disease. Biochimie *92*, 1186–1198.

Kopp, H.G., Avecilla, S.T., Hooper, A.T., Shmelkov, S.V., Ramos, C.A., Zhang, F., and Rafii, S. (2005). Tie2 activation contributes to hemangiogenic regeneration after myelosuppression. Blood *106*, 505–513.

Krause, D.S., Fulzele, K., Catic, A., Sun, C.C., Dombkowski, D., Hurley, M.P., Lezeau, S., Attar, E., Wu, J.Y., Lin, H.Y., et al. (2013). Differential regulation of myeloid leukemias by the bone marrow microenvironment. Nat. Med. *19*, 1513–1517.

Lassailly, F., Foster, K., Lopez-Onieva, L., Currie, E., and Bonnet, D. (2013). Multimodal imaging reveals structural and functional heterogeneity in different bone marrow compartments: functional implications on hematopoietic stem cells. Blood *122*, 1730–1740.

Lee, J.Y., and Kim, H.J. (2014). (Lymph)angiogenic influences on hematopoietic cells in acute myeloid leukemia. Exp. Mol. Med. *46*, e122.

Lewandowski, D., Barroca, V., Duconge, F., Bayer, J., Van Nhieu, J.T., Pestourie, C., Fouchet, P., Tavitian, B., and Romeo, P.H. (2010). In vivo cellular imaging pinpoints the role of reactive oxygen species in the early steps of adult hematopoietic reconstitution. Blood *115*, 443–452.

Li, B., and Dewey, C.N. (2011). RSEM: accurate transcript quantification from RNA-Seq data with or without a reference genome. BMC Bioinformatics *12*, 323.

Lo Celso, C., Fleming, H.E., Wu, J.W., Zhao, C.X., Miake-Lye, S., Fujisaki, J., Cote, D., Rowe, D.W., Lin, C.P., and Scadden, D.T. (2009). Live-animal tracking of individual haematopoietic stem/progenitor cells in their niche. Nature *457*, 92–96.

Lobov, I.B., Cheung, E., Wudali, R., Cao, J., Halasz, G., Wei, Y., Economides, A., Lin, H.C., Papadopoulos, N., Yancopoulos, G.D., and Wiegand, S.J. (2011). The Dll4/Notch pathway controls postangiogenic blood vessel remodeling and regression by modulating vasoconstriction and blood flow. Blood *117*, 6728–6737.

Love, M.I., Huber, W., and Anders, S. (2014). Moderated estimation of fold change and dispersion for RNA-seq data with DESeq2. Genome Biol. *15*, 550.

Mattison, R., Jumonville, A., Flynn, P.J., Moreno-Aspitia, A., Erlichman, C., LaPlant, B., and Juckett, M.B. (2015). A phase II study of AZD2171 (cediranib) in the treatment of patients with acute myeloid leukemia or high-risk myelodysplastic syndrome. Leuk. Lymphoma *56*, 2061–2066.

Mayer, R.J., Davis, R.B., Schiffer, C.A., Berg, D.T., Powell, B.L., Schulman, P., Omura, G.A., Moore, J.O., McIntyre, O.R., and Frei, E., 3rd (1994). Intensive postremission chemotherapy in adults with acute myeloid leukemia. Cancer and Leukemia Group B. N. Engl. J. Med. *331*, 896–903.

Mayhan, W.G. (1999). VEGF increases permeability of the blood-brain barrier via a nitric oxide synthase/cGMP-dependent pathway. Am. J. Physiol. *276*, C1148–C1153.

McIntosh, B.E., Brown, M.E., Duffin, B.M., Maufort, J.P., Vereide, D.T., Slukvin, I.I., and Thomson, J.A. (2015). Nonirradiated NOD,B6.SCID Il2rgamma-/- Kit(W41/W41) (NBSGW) mice support multilineage engraftment of human hematopoietic cells. Stem Cell Rep. *4*, 171–180.

Mendez-Ferrer, S., Scadden, D.T., and Sanchez-Aguilera, A. (2015). Bone marrow stem cells: current and emerging concepts. Ann. N. Y. Acad. Sci. *1335*, 32–44.

Mittal, M., Roth, M., Konig, P., Hofmann, S., Dony, E., Goyal, P., Selbitz, A.C., Schermuly, R.T., Ghofrani, H.A., Kwapiszewska, G., et al. (2007). Hypoxia-dependent regulation of nonphagocytic NADPH oxidase subunit NOX4 in the pulmonary vasculature. Circ. Res. *101*, 258–267.

Muether, P.S., Dell, S., Kociok, N., Zahn, G., Stragies, R., Vossmeyer, D., and Joussen, A.M. (2007). The role of integrin alpha5beta1 in the regulation of corneal neovascularization. Exp. Eye Res. *85*, 356–365.

Ozkor, M.A., Hayek, S.S., Rahman, A.M., Murrow, J.R., Kavtaradze, N., Lin, J., Manatunga, A., and Quyyumi, A.A. (2015). Contribution of endothelium-derived hyperpolarizing factor to exercise-induced vasodilation in health and hypercholesterolemia. Vasc. Med. *20*, 14–22.

Padera, T.P., Stoll, B.R., Tooredman, J.B., Capen, D., di Tomaso, E., and Jain, R.K. (2004). Pathology: cancer cells compress intratumour vessels. Nature *427*, 695.

Padro, T., Ruiz, S., Bieker, R., Burger, H., Steins, M., Kienast, J., Buchner, T., Berdel, W.E., and Mesters, R.M. (2000). Increased angiogenesis in the bone marrow of patients with acute myeloid leukemia. Blood *95*, 2637–2644.

Pan, H., Mostoslavsky, G., Eruslanov, E., Kotton, D.N., and Kramnik, I. (2008). Dual-promoter lentiviral system allows inducible expression of noxious proteins in macrophages. J. Immunol. Methods *329*, 31–44.

Potente, M., and Carmeliet, P. (2017). The link between angiogenesis and endothelial metabolism. Annu. Rev. Physiol. *79*, 43–66.

Poulos, M.G., Gars, E.J., Gutkin, M.C., Kloss, C.C., Ginsberg, M., Scandura, J.M., Rafii, S., and Butler, J.M. (2014). Activation of the vascular niche supports leukemic progression and resistance to chemotherapy. Exp. Hematol. *42*, 976–986.e3.

R Development Core Team. (2015). A Language and Environment for Statistical Computing (R Foundation for Statistical Computing).

Raaijmakers, M.H., Mukherjee, S., Guo, S., Zhang, S., Kobayashi, T., Schoonmaker, J.A., Ebert, B.L., Al-Shahrour, F., Hasserjian, R.P., Scadden, E.O., et al. (2010). Bone progenitor dysfunction induces myelodysplasia and secondary leukaemia. Nature *464*, 852–857.

Reddy, V., and Moreb, J. (2000). Prognostic correlation of increased angiogenesis in acute myeloid leukemia with cytogenetics. Blood *96*, 1617–1618.

Ribatti, D., Vacca, A., Rusnati, M., and Presta, M. (2007). The discovery of basic fibroblast growth factor/fibroblast growth factor-2 and its role in haematological malignancies. Cytokine Growth Factor. Rev. *18*, 327–334.

Roboz, G.J., Giles, F.J., List, A.F., Cortes, J.E., Carlin, R., Kowalski, M., Bilic, S., Masson, E., Rosamilia, M., Schuster, M.W., et al. (2006). Phase 1 study of PTK787/ZK 222584, a small molecule tyrosine kinase receptor inhibitor, for the treatment of acute myeloid leukemia and myelodysplastic syndrome. Leukemia *20*, 952–957.

Rodrigues, S.F., and Granger, D.N. (2015). Blood cells and endothelial barrier function. Tissue Barriers *3*, e978720.

Sant, M., Allemani, C., Tereanu, C., De Angelis, R., Capocaccia, R., Visser, O., Marcos-Gragera, R., Maynadie, M., Simonetti, A., Lutz, J.M., et al. (2010). Incidence of hematologic malignancies in Europe by morphologic subtype: results of the HAEMACARE project. Blood *116*, 3724–3734.

Schepers, K., Pietras, E.M., Reynaud, D., Flach, J., Binnewies, M., Garg, T., Wagers, A.J., Hsiao, E.C., and Passegue, E. (2013). Myeloproliferative neoplasia remodels the endosteal bone marrow niche into a self-reinforcing leukemic niche. Cell Stem Cell *13*, 285–299.

Schmidt, T., Kharabi Masouleh, B., Loges, S., Cauwenberghs, S., Fraisl, P., Maes, C., Jonckx, B., De Keersmaecker, K., Kleppe, M., Tjwa, M., et al. (2011). Loss or inhibition of stromal-derived PlGF prolongs survival of mice with imatinib-resistant Bcr-Abl1(+) leukemia. Cancer Cell *19*, 740–753.

Shirota, T., and Tavassoli, M. (1991). Cyclophosphamide-induced alterations of bone marrow endothelium: implications in homing of marrow cells after transplantation. Exp. Hematol. *19*, 369–373.

Sipkins, D.A., Wei, X., Wu, J.W., Runnels, J.M., Cote, D., Means, T.K., Luster, A.D., Scadden, D.T., and Lin, C.P. (2005). In vivo imaging of specialized bone marrow endothelial microdomains for tumour engraftment. Nature *435*, 969–973.

Stenzel, D., Lundkvist, A., Sauvaget, D., Busse, M., Graupera, M., van der Flier, A., Wijelath, E.S., Murray, J., Sobel, M., Costell, M., et al. (2011). Integrin-dependent and -independent functions of astrocytic fibronectin in retinal angiogenesis. Development *138*, 4451–4463.

Stylianopoulos, T., and Jain, R.K. (2013). Combining two strategies to improve perfusion and drug delivery in solid tumors. Proc. Natl. Acad. Sci. USA *110*, 18632–18637.

Subramanian, A., Tamayo, P., Mootha, V.K., Mukherjee, S., Ebert, B.L., Gillette, M.A., Paulovich, A., Pomeroy, S.L., Golub, T.R., Lander, E.S., and Mesirov, J.P. (2005). Gene set enrichment analysis: a knowledge-based

approach for interpreting genome-wide expression profiles. Proc. Natl. Acad. Sci. USA *102*, 15545–15550.

Thomas, S.R., Chen, K., and Keaney, J.F., Jr. (2002). Hydrogen peroxide activates endothelial nitric-oxide synthase through coordinated phosphorylation and dephosphorylation via a phosphoinositide 3-kinase-dependent signaling pathway. J. Biol. Chem. *277*, 6017–6024.

Ulyanova, T., Priestley, G.V., Nakamoto, B., Jiang, Y., and Papayannopoulou, T. (2007). VCAM-1 ablation in nonhematopoietic cells in MxCre+ VCAM-1f/f mice is variable and dictates their phenotype. Exp. Hematol. *35*, 565–571.

Wang, Z.Y., and Chen, Z. (2008). Acute promyelocytic leukemia: from highly fatal to highly curable. Blood *111*, 2505–2515.

Weis, S.M., and Cheresh, D.A. (2011). alphaV integrins in angiogenesis and cancer. Cold Spring Harbor Perspect. Med. *1*, a006478.

Wong, P.P., Demircioglu, F., Ghazaly, E., Alrawashdeh, W., Stratford, M.R., Scudamore, C.L., Cereser, B., Crnogorac-Jurcevic, T., McDonald, S., Elia, G., et al. (2015). Dual-action combination therapy enhances angiogenesis while reducing tumor growth and spread. Cancer Cell *27*, 123–137.

Zahiragic, L., Schliemann, C., Bieker, R., Thoennissen, N.H., Burow, K., Kramer, C., Zuhlsdorf, M., Berdel, W.E., and Mesters, R.M. (2007). Bevacizumab reduces VEGF expression in patients with relapsed and refractory acute myeloid leukemia without clinical antileukemic activity. Leukemia *21*, 1310–1312.

Zhang, Q., Malik, P., Pandey, D., Gupta, S., Jagnandan, D., Belin de Chantemele, E., Banfi, B., Marrero, M.B., Rudic, R.D., Stepp, D.W., and Fulton, D.J. (2008). Paradoxical activation of endothelial nitric oxide synthase by NADPH oxidase. Arterioscler. Thromb. Vasc. Biol. *28*, 1627–1633.

Zhang, B., Ho, Y.W., Huang, Q., Maeda, T., Lin, A., Lee, S.U., Hair, A., Holyoake, T.L., Huettner, C., and Bhatia, R. (2012). Altered microenvironmental regulation of leukemic and normal stem cells in chronic myelogenous leukemia. Cancer Cell *21*, 577–592.

Zhu, Z., Hattori, K., Zhang, H., Jimenez, X., Ludwig, D.L., Dias, S., Kussie, P., Koo, H., Kim, H.J., Lu, D., et al. (2003). Inhibition of human leukemia in an animal model with human antibodies directed against vascular endothelial growth factor receptor 2. Correlation between antibody affinity and biological activity. Leukemia *17*, 604–611.

STAR★METHODS

KEY RESOURCES TABLE

REAGENT or RESOURCE	SOURCE	IDENTIFIER
Antibodies		
CD45 30-F11 1 in 400 Mouse	eBioscience	Cat: 45-0451-82; RRID: AB_1107002
TER119 Ter-119 1 in 400 Mouse	eBioscience	Cat:12-5921-82; RRID: AB_466042
CD31 390 1 in 400 Mouse	eBioscience	Cat: 25-0311-82; RRID: AB_469616
Endomucin V.7C7 1 in 100 Mouse	Santa Cruz Biotechnology	Cat: sc-65495; RRID: AB_2100037)
Sca-1 D7 1 in 400 Mouse	eBioscience	Cat: 17-5981-83 RRID: AB_469488
Lin - 1 in 400 Mouse	Biolegend	Cat: 133311; RRID: AB_11203535
Kit 2B8 1 in 400 Mouse	BD Bioscience	Cat: 553354; RRID: AB_394805
CD48 HM48-1 1 in 400 Mouse	Biolegend	Cat: 103432; RRID: AB_256163
CD150 TC15-12F12.2 1 in 400 Mouse	Biolegend	Cat: 115914; RRID: AB_439797
IB4 - 100ug/mouse Mouse	Life Technology	Cat: I21411; RRID: AB_2314662
Sca-1/Ly6A/E EPR3355 1 in 100 Mouse	Abcam	Cat: ab109211; RRID: AB_10862573
Ki67 SolA15 2 in 100 Mouse	eBioscience	Cat: 12-5698-82; RRID: AB_11150954
Ki67 20Raj1 2 in 100 Human	eBioscience	Cat: 50-5699-82; RRID: AB_2574237
rAnnexin V - 2 in 100 Mouse/Human	eBioscience	Cat: BMS306; RRID: AB_2687874
CD45 PD7/26 + 2B11 1 in 100 Human	Dako	Cat: M0701; RRID: AB_2314143
CD34 581 1 in 25 Human	BD Bioscience	Cat: 555821; RRID: AB_396150
CD45 2D1 1 in 25 Human	BD Bioscience	Cat: 47-0459-42; RRID: AB_1944368
CD38 HIT2 1 in 25 Human	BD Bioscience	Cat: 555462; RRID: AB_1994368
Ki67 20Raj1 1 in 25 Human	eBioscience	Cat: 50-5699; RRID: AB_2574237
CD45 HI30 20ug/mouse Human	Biolegend	Cat: 304017; RRID: AB_389314
NOS3-pSER1177 C9C3 1 in 100 Mouse/Human	Cell Signaling	Cat: 9570S; RRID: AB_823493
NOS3 33/eNOS 1 in 20 Mouse/Human	BD Bioscience	Cat: 560103; RRID: AB_1645506
NOS2 CXNFT 1 in 100 Mouse/Human	eBioscience	Cat: 17-5920-80; RRID: AB_2573243
Bacterial and Virus Strains		
pHAGE2-EF1aFull-MCS-IRES-Tomato	Kind gift of G. Mostoslavsky; modified from J Immunol Methods. 2008;329:31–44	N/A
pTWG-CMV-LUC-eGFP	Modified from pTWG-eGFP (Addgene) and N/A pGL4-50 luciferase reporter (Promega)	N/A
Biological Samples		
See Tables 1, S1, and S2	N/A	N/A
Chemicals, Peptides, and Recombinant Proteins		
Hypoxyprobe Plus Kit	Natural Pharmacia International	HP2-1000Kit
HypoxiSense 680 Fluorescent Imaging Agent	PerkinElmer	NEV11070
DAF-FM DA solution	Sigma-Aldrich	D2321
CellROX Deep Red Reagent	Thermo Fisher Scientific	C10422
N^G-Methyl-L-arginine acetate salt (NMMA)	Sigma-Aldrich	M7033
Caveolin-1 scaffolding domain peptide (Cavtratin)	Enzolifesciences	ALX-153-064-M005
S-Nitroso-N-acetyl-DL-penicillamine (SNAP)	Sigma-Aldrich	N3398

(*Continued on next page*)

Continued		
REAGENT or RESOURCE	SOURCE	IDENTIFIER
**Deposited Data**		
RNA-sequencing data in CD31^{+} BM-derived endothelial cells	GSE88905	http://www.ncbi.nlm.nih.gov/geo/query/acc.cgi?acc=GSE88905
**Experimental Models: Cell Lines**		
HL60	ATCC	Cell Service, The Francis Crick Institute
ML1	ATCC	Cell Service, The Francis Crick Institute
U937	ATCC	Cell Service, The Francis Crick Institute
**Experimental Models: Organisms/Strains**		
NOD-SCID IL2Rg^{null} (NSG)	Jackson Laboratory	005557
NSG-*NESTIN-GFP*	The original strain is a kind gift from Dr G. Enikolopov. Back-crossed to NSG background at the BRF, The Francis Crick Institute (generation 8)	N/A
NSG-*NG2-DSRED*	Original strain from Jackson Laboratory (008241). Back-crossed to NSG background at the BRF, The Francis Crick Institute (generation 8)	N/A
C57BL/6	Jackson Laboratory	000664
C57BL/6 *Nos3*-KO	Jackson Laboratory	002684
NSG- Kit$^{W41/W41}$ (NBSGW)	Jackson Laboratory	026622
C57BL/6-*Rag2*-KO-*GammaC*-KO	BRF, The Francis Crick Institute	N/A
**Oligonucleotides**		
Human VEGFA REV	Sigma-Aldrich	5’-TGGCCTTGGTGAGGTTTGATCC-3’
Human NOS2 FW	Sigma-Aldrich	5’-GGTGCTGTATTTCCTTACGAGGCG-3’
Human NOS2 REV	Sigma-Aldrich	5’-CTTGTTAGGAGGTCAAGTAAAGGGC-3’
Human NOS3 FW	Sigma-Aldrich	5’-CGGCATCACCAGGAAGAAGA-3’
Human NOS3 REV	Sigma-Aldrich	5’-CATGAGCGAGGCGGAGAT-3’
Human GAPDH FW	Sigma-Aldrich	5’-GGGAAGGTGAAGGTCGGAG-3’
Human GAPDH REV	Sigma-Aldrich	5’-GGGTCATTGATGGCAACAA-3’
Murine NOS3 FW	Sigma-Aldrich	5’-CCTTCCGCTACCAGCCAGA-3’
Murine NOS3 REV	Sigma-Aldrich	5’-CAGAGATCTTCACTGCATTGGCTA-3’
Murine NOX4 FW	Sigma-Aldrich	5’-ACTTTTCATTGGGCGTCCTC-3’
Murine NOX4 REV	Sigma-Aldrich	5’-AGAACTGGGTCCACAGCAGA-3’
Murine GAPDH FW	Sigma-Aldrich	5’-AACTTTGGCATTGTGGAAGG-3’
Murine GAPDH REV	Sigma-Aldrich	5’-CACATTGGGGGTAGGAACA-3’
**Software and Algorithms**		
ZEN microscope software	Zeiss	N/A
IMARIS 8.3.1 image analysis software	Bitplane	N/A

CONTACT FOR REAGENT AND RESOURCE SHARING

Further information and requests for resources and reagents should be directed to the Lead Contact Dominique Bonnet at dominique.bonnet@crick.ac.uk

Human Primary Samples

Umbilical Cord Blood (CB) samples were obtained from normal full-term deliveries after signed informed consent. AML samples used for xenografts were obtained after informed consent at St Bartholomew’s Hospital (London, UK). Details are listed in Table 1. BM specimens from 19 adult patients with newly diagnosed, untreated AML were also obtained after informed consent at St Bartholomew’s Hospital (London, UK). Patient characteristics are depicted in Table S1. Additional BM biopsies were available from these same 19 patients following chemotherapy (details in Table S2). To establish controls, BM biopsies were obtained either from healthy donors for bone marrow transplantation or from patients with diffuse large cell lymphoma without BM infiltration (Table S2). The

collection and use of all human samples were approved by the East London Research Ethical Committee (REC:06/Q0604/110) and in accordance with the Declaration of Helsinki. Mononuclear cells (MNCs) were isolated by centrifugation using Ficoll-Paque TM PLUS (GE Healthcare Life Sciences, Buckinghamshire, UK). For CB samples, the cells were processed within 24 hours following collection. Cells were enriched for $CD34^+$, using an EasySep Human CD34 Positive Selection kit (StemCell Technologies, Vancouver, Canada) according to the manufacturer's instructions, with a purity of 85 to 99%. T cells were depleted from all AML samples using the anti-CD3 mAb OKT-3 (West Lebanon, USA).

Mouse Models

All animal experiments were performed under the project license (PPL 70/8904) approved by the Home Office of UK and in accordance to The Francis Crick institute animal ethics committee guidelines. *NESTIN-GFP* mice were a kind gift from Dr G. Enikolopov. NOD-SCID $IL2Rg^{null}$ (NSG), C57BL/6, *NG2-DSRED*, *Nos3*-KO, *Rag2*-KO/*GammaC*-KO and NOD.B6-SCID c-$Kit^{W41/W41}$ (NBSGW) strains were obtained from Jackson Laboratory, Bar Harbor, Maine, USA) and were bred at The Francis Crick Institute Biological Resources Facility in individually vented cages (IVCs) under Specific Pathogen Free (SPF) conditions. NSG-*NESTIN-GFP* and NSG-*NG2-DSRED* mice were obtained by back-crossing the original lines into the NSG background (generation 8 or more).

Drug Treatments *In Vivo*

AraC was administrated subcutaneously at the indicated doses (10 mg/kg/day for NSG mice and 60 mg/kg/day for C57BL6 mice) at specific time points, for 7 days, as depicted. To inhibit NOS activity, mice received intraperitoneal injection of NMMA (20 mg/kg/day; Sigma-Aldrich, Gillingham, Dorset, UK) or Cavtratin (2.5 mg/kg/day, Enzo LifeScience), or vehicle, for 2 weeks, 5 days/week. All animal work was in accordance to the Home Office and The Francis Crick guidelines.

Intravital Two-Photon Microscopy

For intravital imaging in time-lapse, mice were anesthetized with 2.5% Isoflurane, the head shaved and held in a stereotaxic skull holder. A skin incision revealed the calvaria and methylcellulose (4%, Sigma-Aldrich, Gillingham, Dorset, UK) was applied to prevent the tissue drying. For imaging at a single time point, mice were euthanized by cervical dislocation, the skin was removed, and the intact skull was immerged in PBS for direct examination of the calvarium under the microscope. Images were obtained on a Zeiss 710 NLO laser scanning multiphoton microscope with a 20x 1.0 NA water immersion lens. The microscope was equipped with a MaiTai "High Performance" fully automated 1-box 517 mode-locked Ti:Sapphire laser with DeepSee dispersion compensation (Spectra-Physics), tuned to 890 nm excitation wavelength. To assess the hypoxia with hypoxisense probe, mice received an i.v. injection of 0.4 mg of human normal immunoglobulin twice (48 and 24 hours prior to imaging) (Gammaplex, Bio Products Laboratory, Hertfordshire, UK). The HypoxySense 680 probe (ref: NEV11070, PerkinElmer, Massachusetts, US) was injected 24 hours prior imaging (according to the manufacturer instructions), and on the day of imaging each mouse was intravenously injected with 100 µl of human CD45 AF488 antibody (Biolegend, Cat:304017) 30 minutes prior sacrifice, or with 150 kDa TRITC-dextran in case of NSG-*Nestin-GFP* mice. Mice were imaged as described above. Bone signal (Second Harmonic Generation, SHG) was collected at 380-485nm, hCD45 or Nestin-GFP at 500-550nm, TRITC-dextran at 555-625nm and Hypoxisense at 640-690nm with a 750 nm Infra Red filter by not descanned detectors. To assess vascular perfusion, 100 ug of IB4-AF488 (Life Technology, Cat: I21411) were i.v. injected into mice 30 min prior imaging. 15 µl of Qtracker® 655 Vascular Labels (Thermo Fisher Scientific, Cat: Q21021MP) were injected i.v. 1 min prior imaging. SHG was collected at 380-485nm, IB4-AF488 at 500-550nm, and Qtracker at 640-690nm by not descanned detectors. To assess the hypoxic state of the vasculature, NSG-*Nestin-GFP* mice were used, and TRITC-dextran (150 kDa) was intravenously injected 1 min before imaging to label the vascular tree. SHG was collected at 380-485nm, Nestin-GFP at 500-550nm, TRITC dextran at 555-625nm and Hypoxisense at 640-690nm with a 750 nm Infra Red filter by not descanned detectors. To image the vascular leakiness, we used the strategy detailed in Figure S2E. Briefly, two vessel-pooling agents of different molecular weights were intravenously injected simultaneously: Qtracker® 655 Vascular Labels (Thermo Fisher Scientific, Cat: Q21021MP), high molecular weight and 65-85 kDa TRITRC dextran, medium molecular weight (Sigma-Aldrich, Gillingham, Dorset, UK). Mice were imaged at different time points (Figure 2C) after probes injection, or 10 min after probes administration in all other cases. Calvarium was imaged. SHG was collected at 380-485nm, GFP, 65-85 kDa TRITC dextran at 555-625nm, and 655- Qtracker at 640-690nm by not descanned detectors. Each z stack of images (100-150 µM) was rendered in 3D using Imaris software (Bitplane). At least 9 images representing different areas of bone were taken per group.

Images Processing

To measure vessel mean diameter, the 'filament" algorithm was applied using the signal intensity derived from the NT-Qdot in the BM vasculature (Imaris, Bitplane). To measure the distance to hypoxic areas of BM vessels, a volumetric iso-surface was generated using the signal intensity derived from the Hypoxisense. A map of distances from the Hypoxisense iso-surface was calculated with "Distance Transformation" tool using Imaris XT extension. A volumetric iso-surface to define the vessels was then generated using the intensity derived from the dextran. The distance between each vessel and the closest hypoxiprobe pixel was measured (Bitplane). To assess the vascular leakiness, the signal intensity derived from the Qtracker was used to generate a volumetric iso-surface of the BM vasculature, using the standard surface algoritm of IMARIS. "Background Subtraction" (5-10% based on local contrast) was performed with manual threshold. The iso-surface was then used to segment the signal intensity (SUM) derived from the dextran in "IN" and "OUT" the vasculature, using the "Mask Settings" function. The ratio between these two intensities (OUT/IN) represents

the leakiness of the vasculature at a given time point (Imaris, Bitplane). To measure the distance of AML cells to vessels, a volumetric iso-surface was generated using the signal intensity derived from the dextran. A map of distances from the Hypoxisense iso-surface was calculated with ''Distance Transformation'' tool using Imaris XT extension. The spot function was used to identify hCD45$^+$ cells. The distance between each spot and the closest vascular pixel was measured (Bitplane). To quantify the hypoxia, in each z-stack the hypoxisense signal intensity and the total volume of the z-stack (voxel) were calculated. The hypoxia was expressed as signal intensity/voxel (Imaris, Bitplane).

Bioluminescence Imaging

Before imaging, each mouse was given an intraperitoneal injection of 125 mg/kg luciferin (Caliper Life Science). General anesthesia was induced with 2% isoflurane and the mouse was placed in a light-tight heated chamber. After acquiring bright-field images of each mouse, anterior and posterior luminescent images were then successively acquired with 1- to 5-min exposure time. Optical images were displayed and analyzed with the IVIS Living Image software (Caliper Life Science). Regions of interest were manually drawn around the bodies of the mice to assess the intensity of the bioluminescence signal for each mouse. Optical signal was expressed as photon count.

Cell Lines

293 HEK were grown in DMEM; HL60, ML1 and U937 were grown in RPMI1640, MS5 in IMDM. All cell lines were tested for mycoplasma prior to commencing experiments. All cell lines came originally ATCC (distributor LGC standards, UK) and were grown by our cell service at the Institute. Before use these lines, they were authenticated using the Short Tandem Repeat (SRF) profiling. All media were supplemented with 10% FBS and 1x Penicillin-Streptomycin and all reagents were from Gibco®-Life Technologies (Paisley, UK).

Transduction and Culture of Human Primary Samples

T-cell depleted hAML MNCs were pre-stimulated in StemSpam SFEM (StemCell Technologies, Vancouver, Canada) supplemented with 25 ng/ml G-CSF or 100ng/ml SCF, 100ng/ml Flt3-L, 60ng/ml IL-3 and 10ng/ml TPO (PrepoTech, London, UK), respectively for 4 to 6 hours. CD34-enriched HSPCs were pre-stimulated in StemSpam SFEM (StemCell Technologies, Vancouver, Canada) supplemented with 25 ng/ml G-CSF, 150ng/ml SCF, 150ng/ml Flt3-L, 10ng/ml IL-6 and 20ng/ml TPO (PrepoTech, London, UK) for 4 to 6 hours. Lentiviral supernatant (GFP-Luciferase vector for human AML MNCs and tomato vector (Pan et al., 2008) for HSPCs) was added at a multiplicity of infection (MOI) of 30 in the presence of 5μg/mL polybrene for 12 hours. The cells were then washed and expanded in culture. The efficiency of transduction was analyzed after 4 days by GFP expression. Primary hAML cells were co-cultured on a layer of primary human MSC for one week. Transduced AML cells were co-cultured on MS-5 cells for 4 days before sorting on the human CD45 and GFP fraction and injected into mice. Cells were grown in Myelocult H5100 medium (StemCell Technologies, Vancouver) supplemented with 20ng/ml IL-3, 20ng/ml G-CSF and 20ng/ml TPO (PeproTech, London, UK). Transduced HSPC cells were grown in StemSpam SFEM supplementer with 150 ng/ml SCF, 150 ng/ml Flt-3 and 20 ng/ml TPO for 4 days before sorting on the human CD45 and tomato fraction and injected into mice.

Transduction and Culture of Murine Primary Samples

Primary murine AML carrying the MLL-AF9 or MLL-ENL translocations were generated as reported (Horton et al., 2009). Leukemic cells retrieved from BM or spleen of primary leukemic mice (MLL-AF9 #1, MLL-ENL #1 and #2) were cultured in RPMI supplemented with 10% FCS, 10 ng/ml IL3, 10 ng/ml IL6, 100 ng/ml SCF (Sigma-Aldrich, Gillingham, Dorset, UK). Cells were infected by spinoculation with a lentivirus expressing GFP-Luciferase (MLL-AF9 #1 and MLL-ENL #1) or tomato (MLL-ENL #2) (centrifugation at 700*g*, 25°C, 45 minutes) in the presence of 5μg/mL polybrene (Sigma-Aldrich, Gillingham, Dorset, UK), as previously described (Horton et al., 2005).

AML and HSPCs Transplantation Assay *In Vivo*

Murine AML

Murine AML cells from culture were transplanted into sub-lethally irradiated C57BL6 mice. Secondary AML cells retrieved from these animals were transplanted into unconditioned WT or *Nos3*-KO mice at the indicated concentrations.

Murine Normal HSC

For HSC *in vivo* functionality assays, murine CD45.1 cells retrieved from NSG mice were isolated using an EasySep murine CD45 Positive Selection kit (StemCell Technologies, Vancouver, Canada) and 250000 cells were transplanted i.v. into sub-lethally irradiated *Rag2*-KO/*GammaC*-KO secondary recipients.

Xenografts

For xenografts assays, human AML cell lines, AML patient-derived samples and HSPC were transplanted into 8- to 12-week old unconditioned NSG or NSG-*NESTIN-GFP* and NSG-*NG2-DSRED* mice by i.v. injection, as indicated. For competitive transplantation experiments, 5,000 tomato$^+$ HSPCs were injected together with 100000 untransduced AML6 cells i.v. into unconditioned NBSGW mice (Jackson Laboratory, BarHarbor, Maine, USA). BM engraftment was assessed by BM aspirate or bioluminescence, when indicated.

Bone Marrow Cell Isolation

At the end of each experiment, animals were euthanized by cervical dislocation and the 6 rear bones collected in cold PBS. To retrieve BM endothelial and mesenchymal cells, bones were dissected in small pieces of 2-3 mm diameter and incubated 30 min at 37 degrees in a HBSS digestion solution containing 2% FBS, DNase I (10 μg/ml), Collagenase (1 mg/ml), Dispase II (5 mg/ml) and Heparin (20 U/ml), all from Sigma-Aldrich, Gillingham, Dorset, UK. Bone pieces were next crashed with mortar and pestle in the same digestion solution and incubated at 37 degrees for 30 min. Cell suspension was then homogenized with a micropipette and filtered with a 100 micron cell strainer.

HO Diffusion

Mice were administrated with two injections of 0.4 mg HO (Sigma-Aldrich, Gillingham, Dorset, UK) at 5 minutes interval, and euthanized 5 minutes later. After cervical dislocation, bones were quickly dissected and immediately immersed in ice-cold PBS containing inhibitors of the APC-efflux pump (Reserpine, Verapamil; Sigma-Aldrich, Gillingham, Dorset, UK) to avoid HO extrusion. Cells were recovered and processed as described above and stained with specific surface antibodies. HO uptake was calculated by dividing the geometric mean fluorescence intensity of the HO blue channel by the same analysis carried out on the same population of cells from the same organ but from an animal not injected with HO (negative control). This corresponds to the specific fluorescence intensity and reflects the actual uptake of HO by the population of interest.

Hypoxiprobe Assessment

Pimonidazole (PIM; Hypoxyprobe) was purchased from Natural Pharmacia International, Inc. Mice were i.v. injected with 125 mg/kg PIM or saline solution and euthanized after 2 hours. For flow cytometry analysis, bones were rapidly harvested and kept in PBS at 4°C. Cells were recovered and processed as described above, and stained with specific surface antibodies. Next, they were permeabilized (BD Cytofix/Cytoperm kit) and stained with specific FITC-MAb1, following manufacturer instructions. Cells from PIM non-injected animals but stained with the same concentration of relevant antibody after permeabilization were used as a negative control to calculate the specific geometric mean fluorescence intensity as previously reported (Lassailly et al., 2013). For IHC analysis, samples were processed as described below, and stained with primary FITC-MAb1 and secondary anti-FITC-HRP following manufacturer instructions.

Flow Cytometry Analysis and Cell Sorting

Flow cytometry analysis was performed using a Fortessa flow cytometer (BD Biosciences, Oxford, UK). AML transduced cells were identified based on their GFP expression. Dead cells and debris were excluded from the analysis using 4,6, diamidino-2-phenylindole (DAPI) staining. Murine HSC (SLAM) were defined by the expression of these surface markers: $CD45^{+}LIN^{-}Kit^{+}CD48^{-}CD150^{+}$, with the exclusion of Tomato+ murine AML cells or hCD45+ human AML cells. DAF-FM probe was used to measure intracellular NO, using manufacturer instructions (D2321, Sigma-Aldrich, Gillingham, Dorset, UK). In particular, bone marrow cells were incubated for 30 min at 37 degrees with the probe at 5 uM, and extensively washed afterwards before analysis. CellROX Deep Red Reagent was used to measure cellular ROS, following manufacturer instructions (C10422 – ThermoFisher Scientific). For intracellular staining, cells were fixed and permeabilized after surface epitope staining using BD Cytofix/Cytoperm kit, following manufacturer instruction (BD Bioscience, Oxford, UK). Cell sorting was performed using a FACS Aria SORP (BD Biosciences, Oxford, UK). Human AML cell lines and primary patient-derived AML cells were sorted according to the expression of GFP and hCD45. Murine endothelial and mesenchymal cells were analyzed and sorted based on the absence of expression of CD45 and Ter119 makers, and the positive expression of CD31 and Nestin-GFP, respectively. Flow cytometry plots displayed in this manuscript represent one set of data points from at least 3 replicates. Antibodies informations are included in the Key Resources Table.

Drug Treatments *In Vitro*

Human ML1 cells were seeded alone or on a confluent layer of MS-5 cells for 24 hours before starting the treatments. NMMA was used at a concentration of 5 mM for 48 hours before assessing apoptosis. Primary AML-derived samples were seeded on a confluent layer of MS-5 cells for 4 days before starting the treatment. Drugs were added to the culture (NMMA at 5 mM and SNAP at 10 μm; Sigma-Aldrich, Gillingham, Dorset, UK) for 5 days before assessing apoptosis and cell cycle.

Reverse Transcriptase and Real Time Quantitative PCR (RT-qPCR)

Total RNA from sorted cell samples was isolated using the RNeasy Mini Kit (Qiagen, Crawley, UK) according to the manufacturer's instructions. mRNA was reverse transcribed by Superscript III Reverse Transcriptase (Invitrogen) with an oligoDT primer (Sigma-Aldrich, Gillingham, Dorset, UK). RT-qPCR was performed with an ABI 7500 FAST Thermal Cycler (Applied Biosystems) using SYBR Green dye (Applied Biosystems). RNA was quantified with the Comparative CT Method and GAPDH was used as a housekeeping gene. The CT values used were the result of triplicates. The primers used are described in the Key Resources Table.

Histological Processing and Immunostaining

Murine samples. Harvested humerus samples were fixed o/n in 10% neutral buffered formalin and then decalcified with 17% EDTA (Osteosoft, Millipore) during 7 days. Samples were processed, paraffin embedded and sectioned (5 μm) for histological studies. Hematoxylin/eosin was performed first to assess quality of the sections of BM samples and visualize vessel integrity. For

immunofluorescence (IF) studies heat antigen retrieval was performed in all cases. Primary antibodies are listed in the key resources table. Goat anti-mouse/rabbit/rat secondaries antibodies coupled to AF-488, AF555 and AF647 were used (all from Invitrogen). DAPI was included in the mounting media to label the nuclei. Images were obtained with a Zeiss LSM710 upright confocal microscope. At least 3 images were taken per sample group. To quantify micro-vascular density (MVD), the number of vessel sprouts/mm2 was counted (Bitplane), and data are show as fold over ctrl.

Sample Preparation for RNA-Sequencing

The quality and concentration of total RNA were determined on Agilent 2100 Bioanalyzer using the Eukaryote Total RNA Pico Assay. Most of the total RNA has average RIN number 5-8, with a concentration at least 4 pg/μl. Some of the samples were concentrated on a speedvac without heat to obtain a final volume of 5 μl. 3.5 ng of Total RNA in 5 μl volume was used to generate cDNA synthesis with Nugen Ovation® RNA-Seq System V2 kit (part No. 7102). The resulting SPIA-cDNA were normalized to 100 ng in 15 μl based on Qubit DNA HS assay. Fragmentation was done using 8 microTUBE-15 AFA Beads Strip V2 (PN 520159) on Covaris E-series at setting 20% DF, 18W, 200 burst, 20C tempt, 120s treatment time and no Intensifier. Only 10-30 ng in 10 μl of fragmented cDNA was used starting from the End Repair step of the Ovation® Ultralow Library Systems V2 (part No. 0344NB) protocol, with 10 cycles PCR. The RNAseq libraries were quality checked on Qubit DNA HS assay, Bioanalyser and Illumina Ecoreal QPCR followed by Illumina PE51 sequencing on Hiseq 2500 V3 reagents.

RNA-Sequencing Analysis Methods

FastQ files were generated using CASAVA BCL to FastQ (version 2.16). Sequencing yield was typically ~25 million strand-specific paired-end reads. The RSEM package (version 1.2.29) (Li and Dewey, 2011) in conjunction with the STAR alignment algorithm (version 2.5.1b) (Dobin et al., 2013) was used for the mapping and subsequent gene-level counting of the sequenced reads with respect to mm10 Ensembl genes downloaded from the UCSC Table Browser 15 on 14th April 2016 (Karolchik et al., 2004). The "–forward-prob" parameter was set to "0" and all other parameters were kept as default. Differential expression analysis was performed with the DESeq2 package (version 1.10.1) (Love et al., 2014) within the R programming environment (version 3.2.3) (R Development Core Team, 2015). Genes with FDR <0.05 were judged to be differentially expressed. Genes from each given pairwise comparison were ranked using the Wald statistic. GSEA pre-ranked analysis was performed with respect to MSigDB (version 5.1) (Subramanian et al., 2005) genesets C2 canonical pathways and C5 GO biological process. All parameters were kept as default except for enrichment statistic (weighted), min size (5) and max size (50000).

Statistical Analysis

Statistics were performed with Prism (GraphPad). Unpaired Student's t-test was used to compare two groups, except in Figure 5E and S3B and S5B, where paired Student's t-test was used. Log-rank test was used for survival curves. Details of each analysis are in figure legends. For probabilities distribution in our cohort of patients, we have considered the joint probability distribution of two Boolean variables: NO normalization and response to therapy. We applied Bayesian inference to estimate the probabilities of each of the four possible combinations of these variables from the cohort of patients. These probabilities are related to the occurrence of each combination of NO normalization and response to therapy, according to a multinomial distribution parameterized by the four probabilities. We estimated these parameters from their posterior distributions obtained by updating a uniform prior using the data available. The posterior distributions for the four probabilities have been obtained by generating a sample of the parameters of the multinomial (N=1 10^6), weighted according to importance sampling. The generated sample was then used to calculate relevant conditional probabilities and their corresponding posterior distributions, which allow the calculation of maximum a posteriori (MAP) estimates and 95% credible intervals (CI95). The analysis was performed using custom-made software written in R (R Development Core Team, 2015). A similar analysis was performed including only intermediate risk group patients.

DATA AND SOFTWARE AVAILABILITY

Data Resources

The accession number for the transcriptome sequencing data generated in this study are deposited on Geo bank (reference number: GSE88905).

Cancer Cell, Volume *32*

Supplemental Information

Increased Vascular Permeability in the Bone Marrow Microenvironment Contributes to Disease Progression and Drug Response in Acute Myeloid Leukemia

Diana Passaro, Alessandro Di Tullio, Ander Abarrategi, Kevin Rouault-Pierre, Katie Foster, Linda Ariza-McNaughton, Beatriz Montaner, Probir Chakravarty, Leena Bhaw, Giovanni Diana, François Lassailly, John Gribben, and Dominique Bonnet

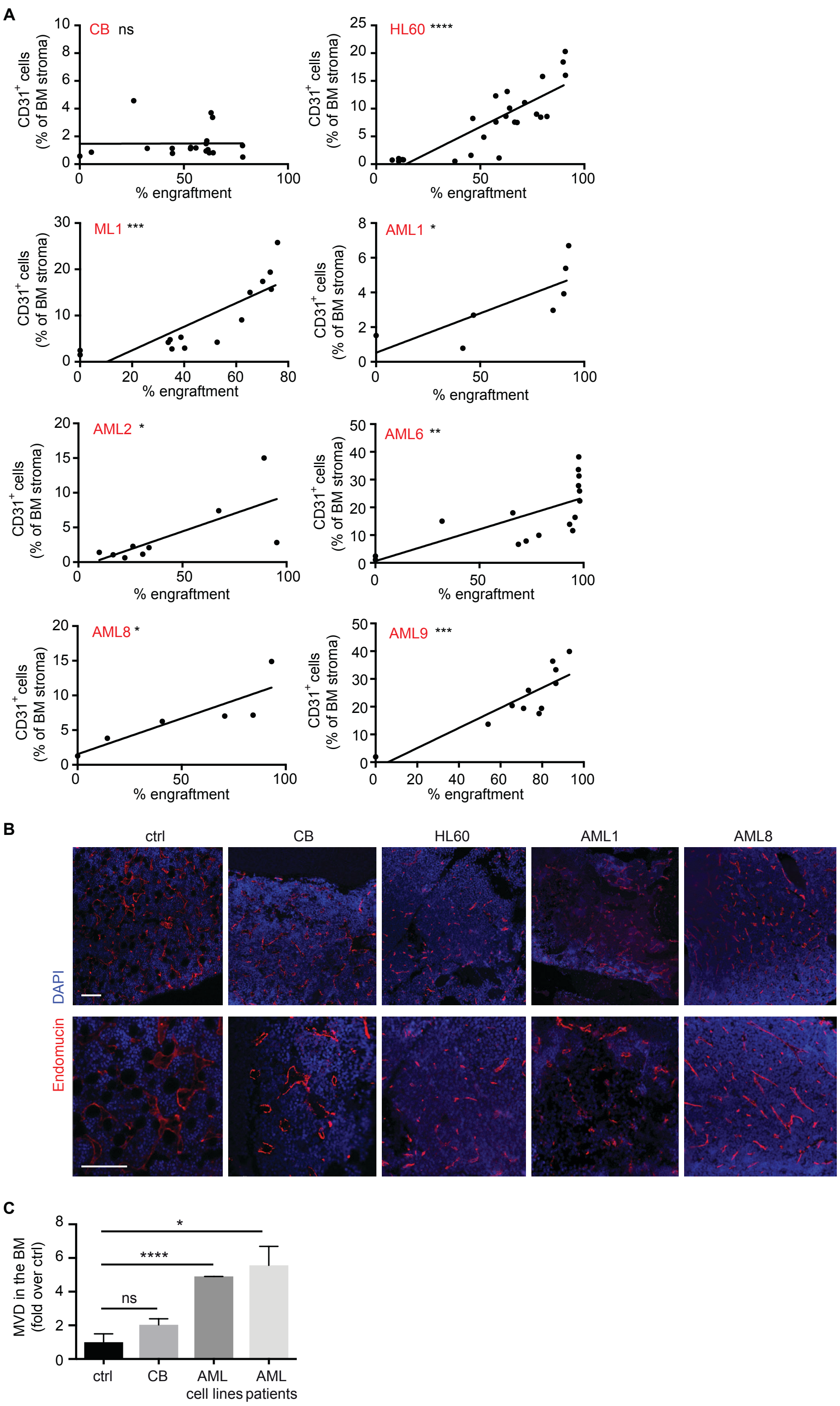
A
CB ns
HL60 ****
ML1 ***
AML1 *
AML2 *
AML6 **
AML8 *
AML9 ***
CD31+ cells (% of BM stroma)
% engraftment
B
ctrl
CB
HL60
AML1
AML8
DAPI
Endomucin
C
MVD in the BM (fold over ctrl)
ns
****
*
ctrl
CB
AML cell lines
AML patients

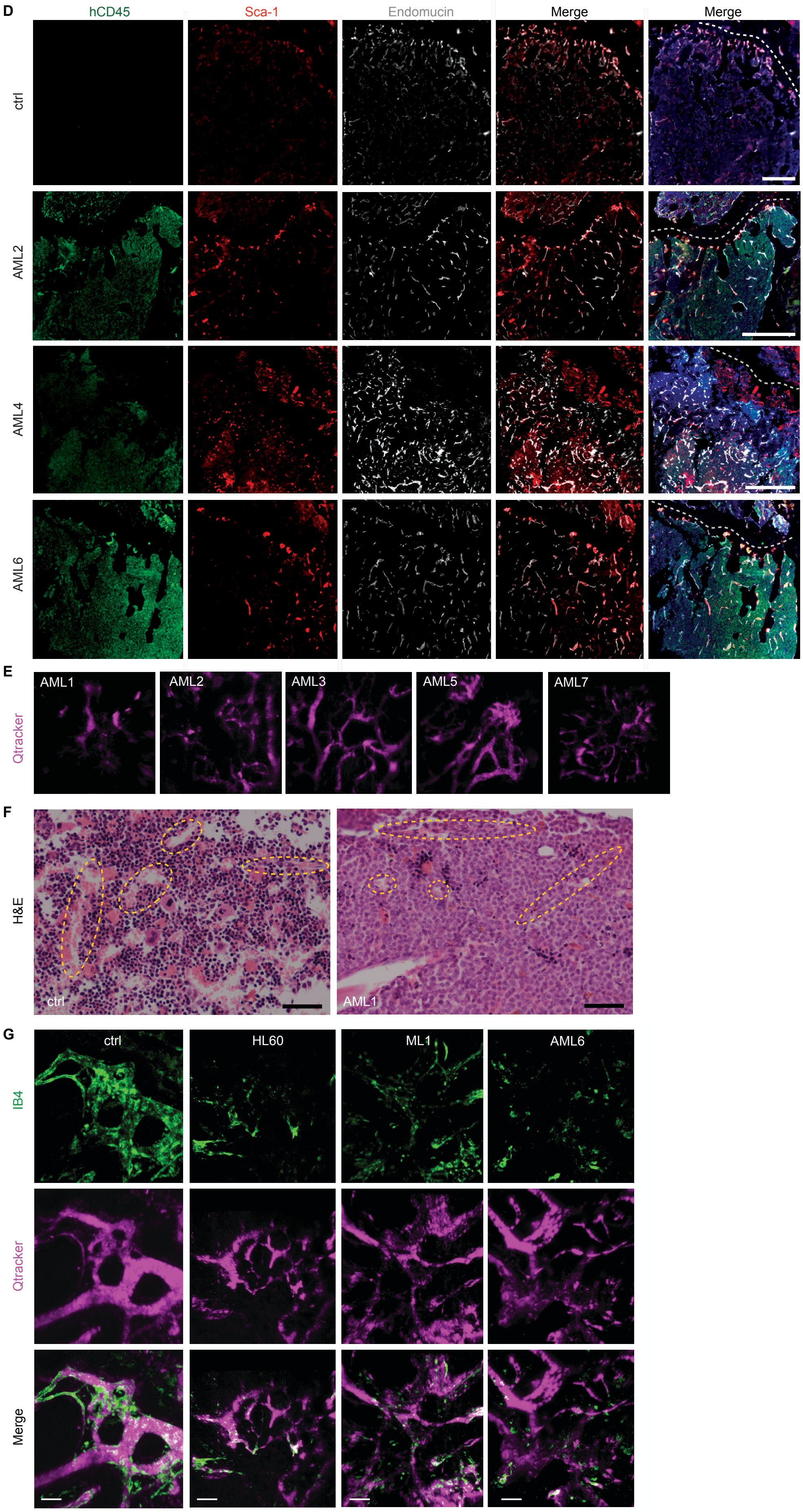
D
hCD45
Sca-1
Endomucin
Merge
Merge
ctrl
AML2
AML4
AML6
E
AML1
AML2
AML3
AML5
AML7
Qtracker
F
H&E
ctrl
AML1
G
ctrl
HL60
ML1
AML6
IB4
Qtracker
Merge

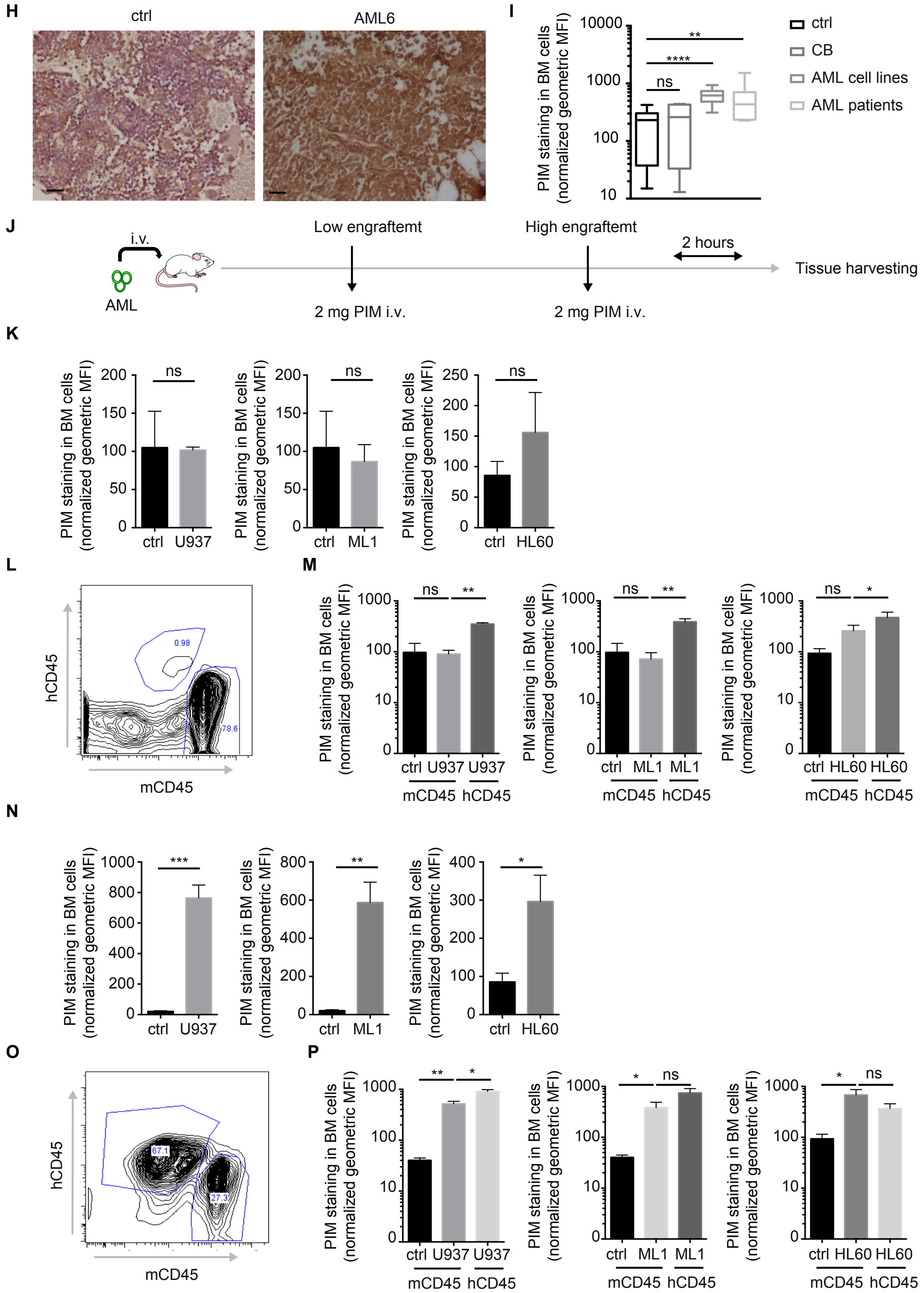

H
ctrl
AML6
I
PIM staining in BM cells (normalized geometric MFI)
10000
1000
100
10
**
****
ns
ctrl
CB
AML cell lines
AML patients
J
i.v.
AML
Low engraftemt
High engraftemt
2 hours
Tissue harvesting
2 mg PIM i.v.
2 mg PIM i.v.
K
PIM staining in BM cells (normalized geometric MFI)
ns
ctrl U937
ctrl ML1
ctrl HL60
L
hCD45
mCD45
0.98
78.6
M
ns **
ns **
ns *
ctrl U937 U937
ctrl ML1 ML1
ctrl HL60 HL60
mCD45 hCD45
N
***
**
*
ctrl U937
ctrl ML1
ctrl HL60
O
hCD45
mCD45
67.1
27.3
P
** *
* ns
* ns
ctrl U937 U937
ctrl ML1 ML1
ctrl HL60 HL60
mCD45 hCD45

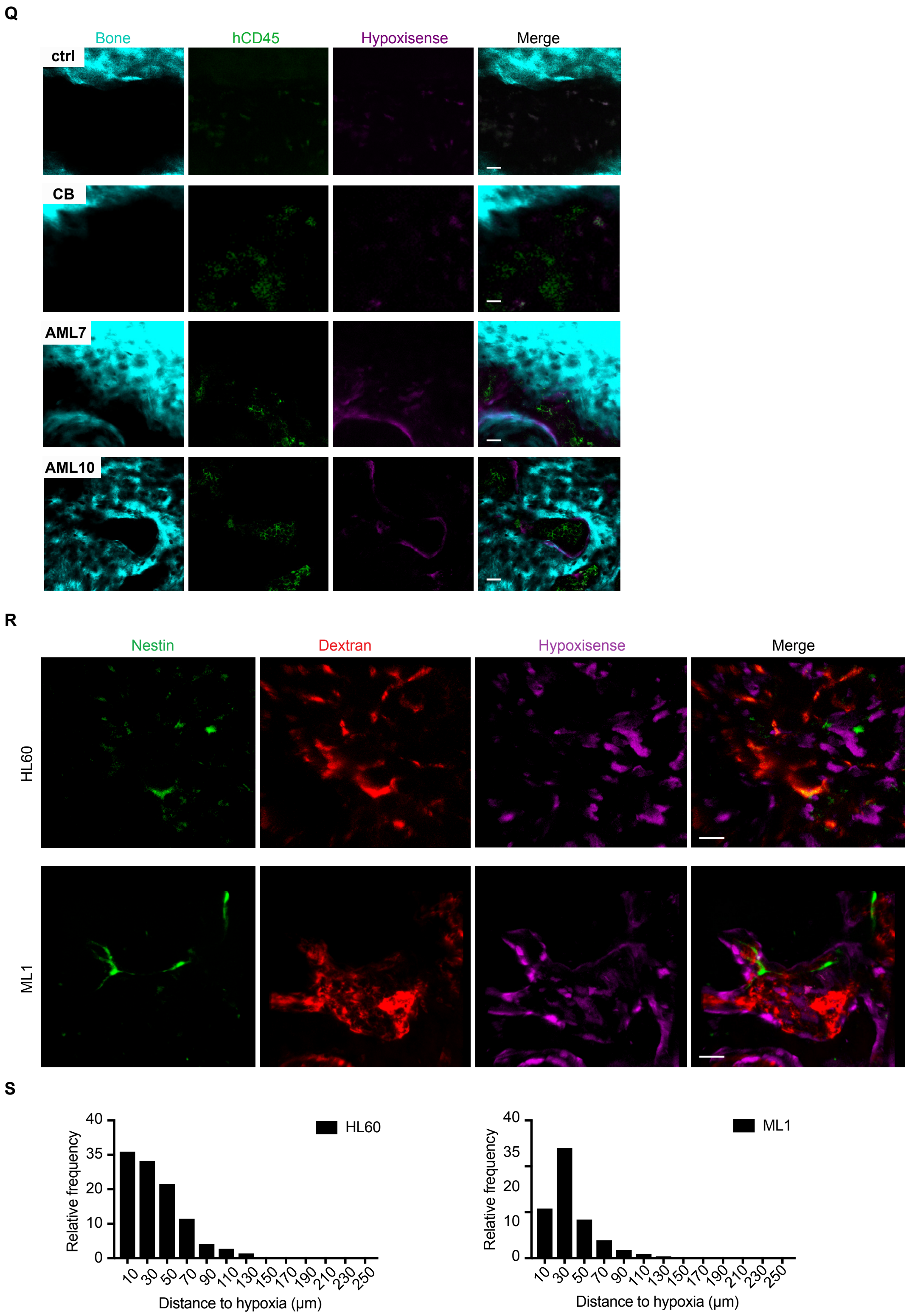

Figure S1. Related to Figure 1. AML derived alteration of the BM vascular architecture and function. (A) Quantification of $CD31^{+}$ endothelial cells in the BM (shown as % of $CD45^{-}Ter119^{-}$ BM cells) in correlation with the human normal or leukemic engraftment, as depicted. Linear regression is shown. **(B)** Immunofluorescence images of fixed long bones showing endomucin (red)

vasculature of non-transplanted mice or mice transplanted with CB derived HSPCs (CB), human HL60 cells or AML patient-derived cells, as depicted. Nuclei are stained with DAPI (blue) Bars represent 200 µm. **(C)** Microvascular density (MVD) quantified via endomucin staining in the BM of non-transplanted mice (ctrl) and mice transplanted with CB-derived HSPCs (CB) or human AML patients' derived samples. Ctrl n=5; CB n=11; AML cell lines n=7; AML patients (AML1, 6) n=9. Data are shown as mean ± s.e.m. **(D)** Immunofluorescence images of fixed long bones of non-transplanted mice or mice transplanted with AML patient-derived samples, showing endomucin (white) and Sca-1 (red) vasculature in association with leukemic cells (hCD45, green). Nuclei are stained with DAPI (blue). Bars represent 500 µm. **(E)** Representative 3D reconstruction of BM vasculature of the calvarium of mice transplanted with AML patient-derived samples, as depicted, imaged via 2P microscopy 1 min after injection of 655-conjugated NT-Qtracker as vessel pooling agent. Data are representative of 5 independent AML derived PDX done in triplicates. Bars represent 70 µm. **(F)** H&E staining of long bones of non-transplanted mice (ctrl) or mice transplanted with AML1 patient-derived cells. Yellow circles indicate vessel lumen. Bars represent 50 µm. **(G)** Representative 3D reconstruction of BM vasculature of the calvarium of non-transplanted mice (ctrl) or AML xenografts, as depicted, imaged via 2P microscopy. IB4 (green) labeling of endothelial cells represents vascular perfusion, and Qtracker (purple) labels the vascular tree. Data are representative of at least 9 replicates per group. Bars represent 70 µm. **(H)** Representative IHCs showing the hypoxic staining via hypoxiprobe of long bones of non-transplanted mice (ctrl) and mice transplanted with AML6 patient-derived cells, as depicted. Bars represent 50 µm. **(I)** Hypoxiprobe staining of BM cells isolated from non-transplanted mice (ctrl) and mice transplanted with CB-derived HSPCs (CB) or AML cell lines or patient-derived samples, as depicted. Ctrl n=14; CB n=6; AML cell lines n=9; AML patients n=11. Data are shown as Whiskers min to max plots, the line inside the box representing the mean, the top and the bottom line representing the 75% and 25% percentiles, respectively, and the lines above and below the box representing the S.D. **(J)** Schematic of the experiment: Mice engrafted with human AML cells were assessed for BM engraftment. At specific time points (low and high engraftment), 2 mg of hypoxiprobe were injected intravenously 2-hours prior sacrifice. Bones were retrieved and hypoxiprobe intracellular adducts were measured either via IHC or intracellular flow cytometry staining. **(K)** Low engraftment**:** hypoxiprobe staining in BM cells of non-transplanted mice or mice transplanted with human AML cell lines, as depicted; n=3 per condition. Data are shown as mean ± s.e.m. **(L)** Flow cytometry gating strategy. **(M)** Hypoxiprobe staining in different populations of BM cells in mice sacrificed at low leukemic engraftment; n=3 per condition. Data are shown as mean ± s.e.m. **(N)** High engraftment: Hypoxiprobe staining in BM cells of non-transplanted mice or mice transplanted with human AML cell lines, as depicted; n=3 per condition. Data are shown as mean ± s.e.m. **(O)** Flow cytometry gating strategy. **(P)** Hypoxiprobe staining in different populations of BM cells in mice sacrificed at high leukemic engraftment; n=3 per condition. Data are shown as mean ± s.e.m. **(Q)** Representative z-stacks of BM hypoxia imaged via intravital microscopy using the hypoxisense probe together with bone (SHG) and $hCD45^{+}$ cells in non-transplanted mice or mice transplanted with CB-derived HSPCs (CB) or AML patient-derived samples, as

depicted. Bars represent 30 µm. **(R)** Representative 3D reconstruction of BM hypoxia imaged via intravital microscopy using the hypoxisense probe together with vasculature (Dextran) and Nestin^{+} cells in mice transplanted with AML cell lines, as depicted. Bars represent 50 µm. **(S)** Distribution and relative frequency of vessel distances to hypoxic areas in the BM of non-transplanted mice (ctrl) or mice transplanted with AML cell lines, as depicted. P values: ns: non-significant; * p<0.05; ** p<0.01; *** p<0.001.

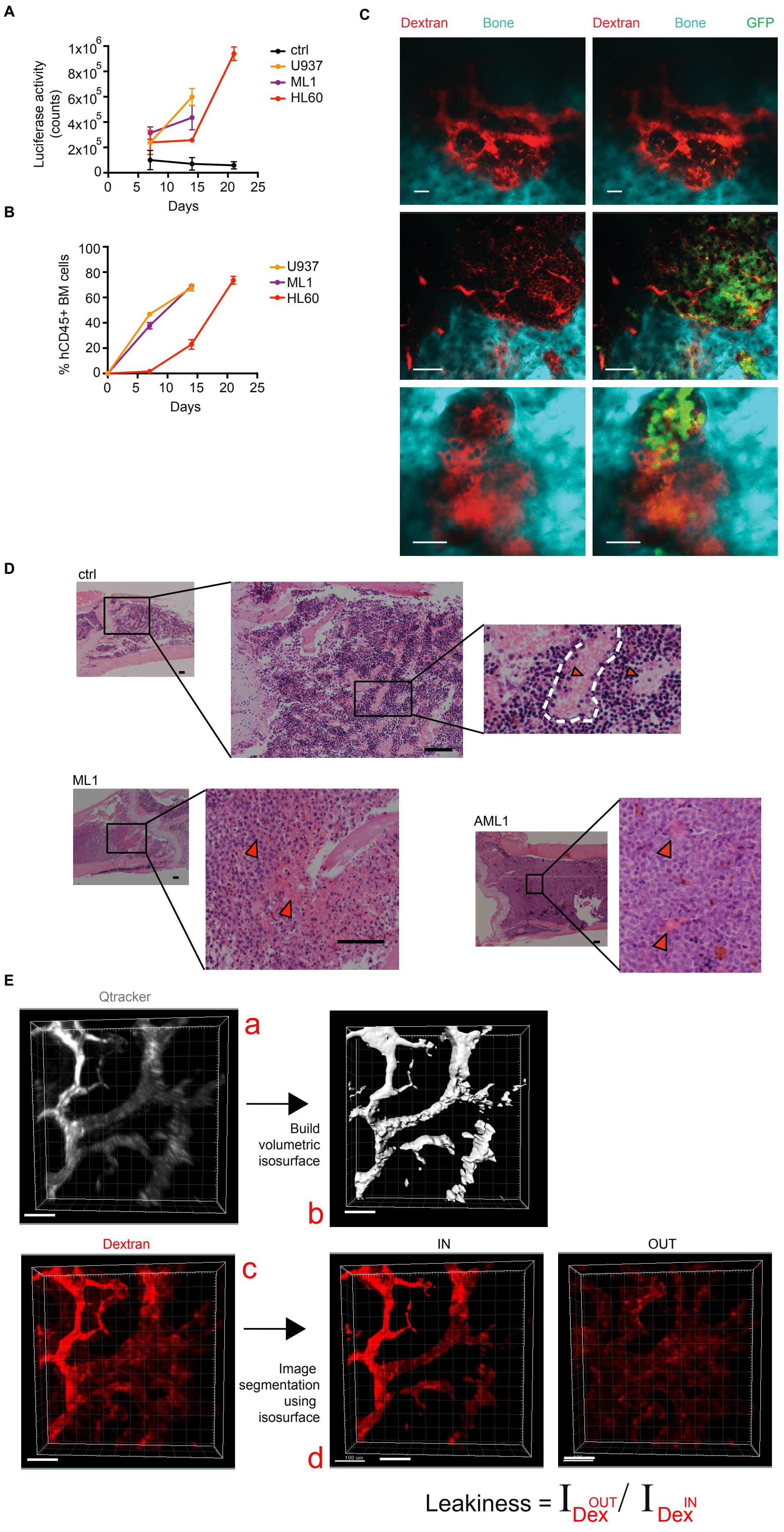

A
Luciferase activity (counts)
1x10^6
8x10^5
6x10^5
4x10^5
2x10^5
0
0 5 10 15 20 25
Days
ctrl
U937
ML1
HL60
B
% hCD45+ BM cells
100
80
60
40
20
0
0 5 10 15 20 25
Days
U937
ML1
HL60
C
Dextran
Bone
Dextran
Bone
GFP
ctrl
U937
ML1
D
ctrl
ML1
AML1
E
Qtracker
a
Build volumetric isosurface
b
Dextran
c
IN
OUT
Image segmentation using isosurface
d
Leakiness = I Dex OUT / I Dex IN

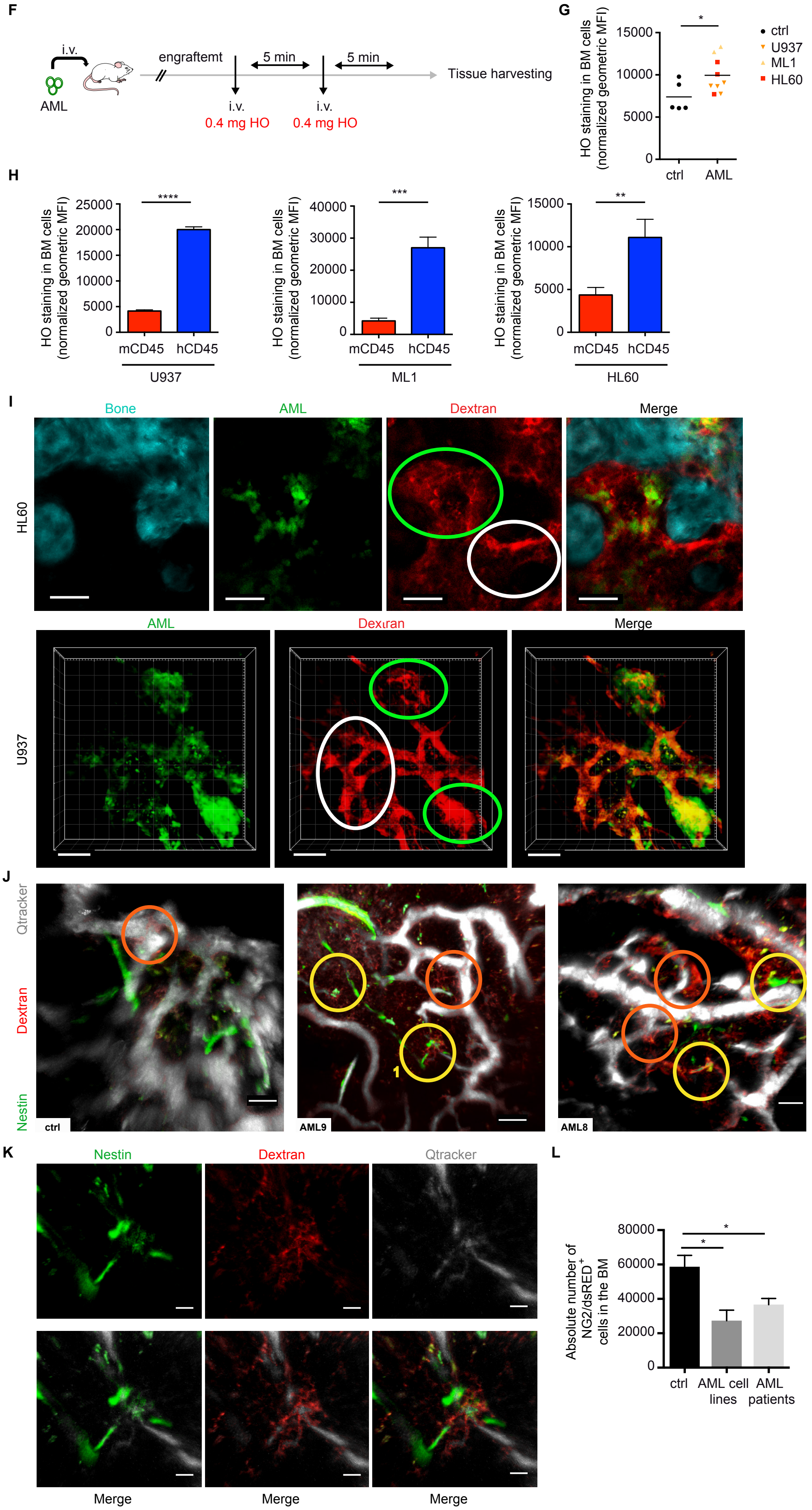
F
i.v.
AML
engraftemt
5 min
5 min
i.v.
0.4 mg HO
i.v.
0.4 mg HO
Tissue harvesting
G
HO staining in BM cells
(normalized geometric MFI)
15000
10000
5000
0
*
ctrl
AML
ctrl
U937
ML1
HL60
H
HO staining in BM cells
(normalized geometric MFI)
25000
20000
15000
10000
5000
0
****
mCD45
hCD45
U937
40000
30000
20000
10000
0
***
mCD45
hCD45
ML1
15000
10000
5000
0
**
mCD45
hCD45
HL60
I
Bone
AML
Dextran
Merge
HL60
AML
Dextran
Merge
U937
J
Qtracker
Dextran
Nestin
ctrl
1
AML9
AML8
K
Nestin
Dextran
Qtracker
Merge
Merge
Merge
L
Absolute number of
NG2/dsRED+
cells in the BM
80000
60000
40000
20000
0
*
*
ctrl
AML cell
lines
AML
patients

Figure S2. Related to Figure 2. AML engraftment increases vascular permeability in the BM. (A) Kinetics of BM engraftment shown as luciferase activity in mice (n=3 per condition) transplanted with human AML cell lines. Data are shown as mean ± s.e.m. **(B)** Kinetics of BM engraftment shown as percentage of hCD45$^+$ cells in the BM of mice (n=3 per condition) transplanted with human AML cell lines. Data are shown as mean ± s.e.m. **(C)** Representative z-stack of the TRITC-Dextran labeled BM vasculature in the calvarium of non-transplanted control or from mice transplanted with GFP-U937 and GFP-ML1 cells. Data are representative of triplicates. Bars represent 30, 70, 50 µm for ctrl, U937 and ML1, respectively. **(D)** H&E staining of the long bones of non-transplanted mice (ctrl) or mice transplanted with AML xenografts, as depicted. Red arrows point to erythrocytes. Bars represent 50 µm**. (E)** The quantification strategy of bone marrow vascular permeability is shown. The volumetric signal derived from the 655-conjugated Qtracker (a) is used to create an isosurface of the vascular tree in the BM (b). This isosurface is used to segment the volumetric signal derived from the Dextran (c) inside and outside the vasculature. The leakiness is shown as a ratio OUT/IN (d). The formula used to measure the leakiness is shown (I = intensity signal). Bars represent 100 µm. **(F)** Schematic of the experiment: HL60, ML1 and U937 human AML cells were injected intravenously into NSG mice. Once engraftment confirmed, two consecutive intravenous injections of 0.4 mg of Hoechst (HO) were performed at 5 mns interval. Mice were sacrificed 5 mns after the last injection, and BM cells retrieved in cold PBS containing inhibitors of HO expulsion. **(G)** HO signal was analyzed by flow cytometry in BM cells of non-transplanted mice or mice engrafted with AML cell lines, as depicted (normalized geometric MFI). Bars represent mean. **(H)** HO signal in murine CD45$^+$ and human CD45$^+$ cells in the BM of mice detailed in B; n=3 per condition. Data are shown as mean ± s.e.m. **(I)** Representative Z-stacks (HL60) and 3D reconstruction (U937) of the BM vasculature in the calvarium of mice after injection of TRITC-Dextran vessel pooling agent. SHG: Second harmonic signal generated by the bone. White ellipses indicate non-engrafted areas; green ellipses indicate AML engrafted areas. Bars represent 50 µm (HL60) and 100 µm (U937). **(J)** Representative 3D reconstruction of the BM vasculature in the calvarium of non-transplanted mice or AML xenografts, as depicted, after injection of Qtracker and Dextran. Nestin-GFP signal (green) labels arteriolar vsculature. Orange and yellow circles indicate Nestin$^-$ and Nestin$^+$, respectively, leaky vessels. Bars represent 50 µm. **(K)** Higher magnification of area n1 highlighted in (J). Bars represent 10 µm. **(L)** Absolute number of NG2-DSRED$^+$ cells in the BM of non-transplanted mice (ctrl, n=3) or mice engrafted with human AML cell lines (HL60 n=2; ML1 n=3) or patient-derived samples (AML6, n=6; AML9, n=5). Bars represent mean ± s.e.m. p values: *p<0.05; *** p< 0.001; **** p<0.0001.

Table S1. Related to Figure 3. BM biopsies obtained from AML patients and donors with normal BM

PATIENT ID	CYTOGENETICS	AGE	GENDER	NPM/FLT3	CLINICAL INFORMATIONS
AML11	T(4;17;15;7)	34	FEMALE	WT/MUT	n.d.
AML12	TRISOMY 8	44	MALE	WT/WT	M1
AML13	T(15;17)	22	FEMALE	WT/WT	M3
AML14	n.d.	46	FEMALE	WT/WT	M1
AML15	T(15;17) +	39	FEMALE	WT/WT	M3
AML16	PLUS X	30	FEMALE	WT/WT	M0
AML17	NORMAL	65	FEMALE	WT/WT	2ND AML
AML18	NORMAL	55	FEMALE	WT/WT	M0
AML19	NORMAL	37	FEMALE	WT/MUT	M5
AML20	NORMAL	62	MALE	WT/WT	2ND AML
AML21	NORMAL	43	MALE	WT/WT	TRIPHENOTYPIC: B,T, M
AML22	NORMAL	49	FEMALE	WT/MUT	M1
AML23	NORMAL	54	MALE	WT/WT	M2
AML24	NORMAL	63	MALE	WT/WT	M5
AML25	NORMAL	64	MALE	MUT/MUT	M0
AML26	T(15;17) +	41	FEMALE	WT/WT	M3
AML27	NORMAL	69	MALE	WT/WT	M2
AML28	INS(10;11)MLL(11Q23)REARRANGED	31	MALE	WT/MUT	M1
AML29	TRISOMY 8	68	MALE	WT/MUT	M2
HC1	-	34	MALE	-	DLBC - Stage IE
HC2	-	45	MALE	-	DLBC - Stage IE - chest wall
HC3	-	86	FEMALE		DLBC - Stage II
HC4	-	69	MALE	-	DLBC - Stage IIE - THYROID
HC5	-	78	MALE	-	DLBC - Stage IE - TESTES
HC6	-	40	FEMALE	-	BMT donor - B-thalassaemia trait
HC7	-	27	FEMALE	-	BMT donor - B-thalassaemia trait
HC8	-	37	FEMALE	-	BMT donor - Sickle cell trait

HC1 to 5 were used as healthy biopsies as they show no BM infiltration of malignant cells.

Table S2. Related to Figure 3. Treatment protocols of patients in Table S1

AML ID	Treatment protocol	Alive
AML11	Induction: AraC, daunorubicin and etoposide	yes
AML12	Induction: ifosfomide, carboplatin and etoposide (ICE) and AraC	yes
AML13	Induction: AraC, daunorubicin and etoposide	no
AML14	Induction: AraC, daunorubicin and etoposide	yes
AML15	Induction: AraC, daunorubicin and etoposide<br>Consolidation: all trans retinoic acid, 6-mercaptopurine and methotrexate	yes
AML16	Induction 1: ifosfomide, carboplatin and etoposide (ICE)<br>Induction 2: daunorubicin and AraC	no
AML17	Induction: idarubicin and AraC	no
AML18	Induction: ifosfomide, carboplatin and etoposide (ICE)	yes
AML19	Induction: AraC, daunorubicin and etoposide	no
AML20	Induction: Daunorubicin and AraC + haematopoetic stem cell transplant	yes
AML21	Induction 1: German multicentre acute lymphoblastic leukeamia trial<br>Induction 2: etoposide and AraC<br>Relapse: intrathecal chemotherapy to treat central nervous system relapse	no
AML22	Induction: AraC, daunorubicin and etoposide + Gemtuzumab ozogamacin<br>Relapse 1: Low dose AraC<br>Replapse 2: daunorubicin and AraC	no
AML23	Induction: AraC, daunorubicin and etoposide	no
AML24	Induction: daunorubicin and AraC + all trans retinoic acid	yes
AML25	Induction: daunorubicin and AraC + Gemtuzumab ozogamacin	yes
AML26	Induction: all trans retinoic acid and arsenic trioxide	yes
AML27	Induction: Azacytidine + low dose AraC<br>Consolidation: low dose AraC	no
AML28	Induction 1: AraC, daunorubicin and etoposide	no
AML29	Induction: Azacytidine	no

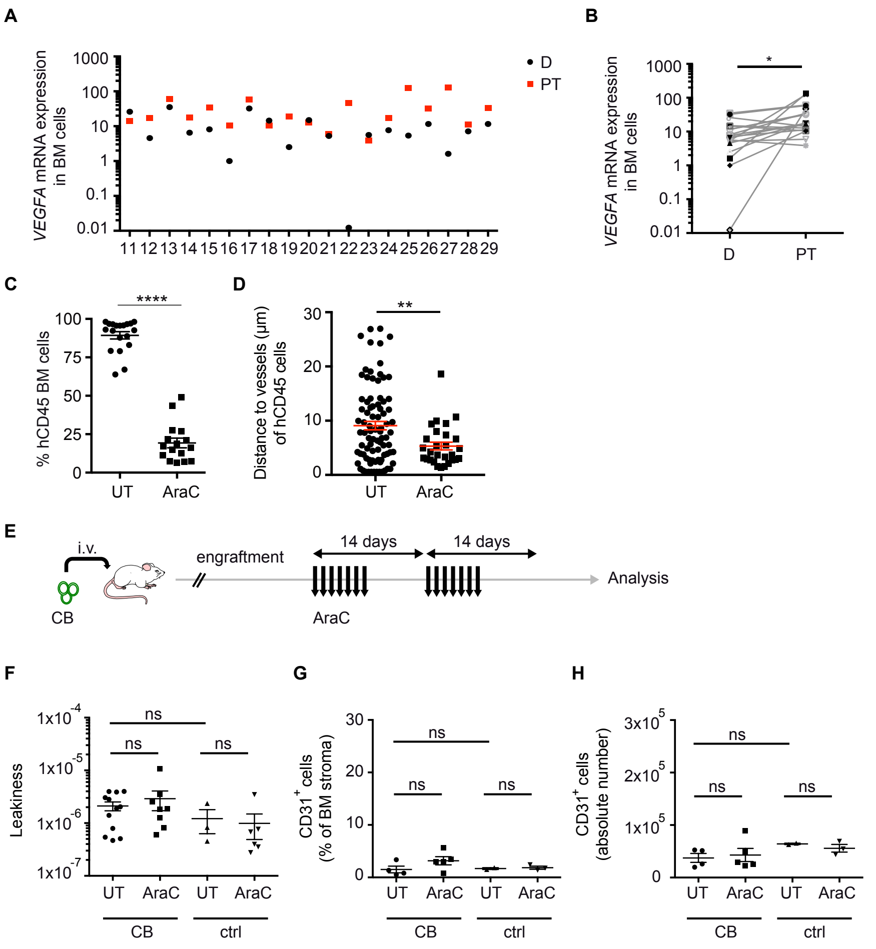

Figure S3. Related to Figure 3. Persistence of increased vascular permeability after chemotherapy. (A) *VEGFA* mRNA relative expression in BMC derived from AML patients at diagnosis (D, black circles) and post-treatment (PT, red squares). **(B)** Comparison of the level of *VEGFA* mRNA relative expression in BMC of each AML patients at diagnosis and post-treatment. **(C)** Percentage of human CD45+ leukemic cells in the BM of mice described, treated with AraC or control solvent. AML patients tested: AML6, 7, 8, 9. UT n=17; AraC n=16. Each dot represents one mouse. Bars represent mean ± s.e.m. **(D)** Measurement of hCD45+ cell distribution in relation to vasculature in the calvaria BM of mice transplanted with AML6 patient-derived cells, and treated or not with AraC, as depicted. Each dot represents one cell. Bars represent mean± s.e.m. UT n=86; AraC n=28. **(E)** Schematic of the experiment. Non-transplanted mice or mice engrafted with human CD34+ cells were treated with 10 mg/kg/day of AraC or solvent, administrated intraperitoneally for two alternated weeks. Vessel-pooling agents were administrated intravenously. Mice were sacrificed 10 min later and calvaria imaged via 2P microscopy. **(F)** Vascular leakiness in the BM of mice detailed in A; n=3 or more replicates. Bars represent mean ± s.e.m. **(G)** Quantification of CD31+ endothelial cells in the BM (shown as % of CD45- Ter119- BM cells) of mice detailed in A. Bars represent mean ± s.e.m. **(H)** Absolute number of CD31+ endothelial cells in the BM (2 femurs, 2 tibias and 2 iliac crests) of mice detailed in A, as depicted. Bars represent mean ± s.e.m. p values: ns: non-significant; * p<0.05; ** p<0.01;*** p<0.001.

A

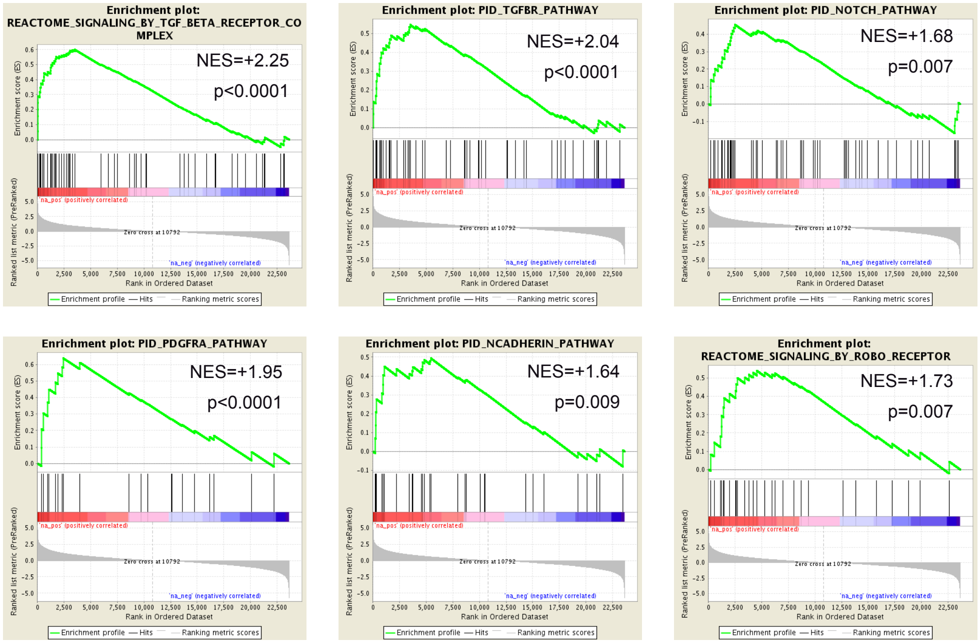

B

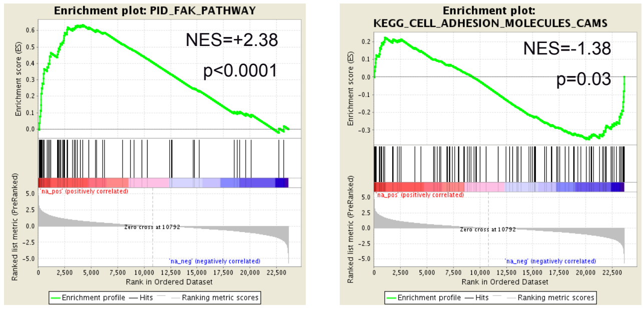

Itgb1	↑
Itga5	↑
Itgav	↑
Itgb5	↑
Itgb7	↓
Cldn1	↓
Itgal	↓
Cdh1	↓
Vcam1	↓
Itga4	↓

Figure S4. Related to Figure 4. AML-induced transcriptional signature in BM ECs. (A) Enrichment plots for Pathways from GSEA between CB and AML groups using RNA-seq gene expression data indicate enrichment of different pro-angiogenic pathways in endothelial cells from AML group. Normalized Enrichment Score (NES) and nominal p value are shown. **(B)** Enrichment plots for Pathways from GSEA between CB and AML groups using RNA-seq gene expression data indicate upregulation of FAK pathway (top) and downregulation of cell adhesion molecules (CAMs) pathway (bottom) in endothelial cells from the AML group. Normalized Enrichment Score (NES) and nominal p values are shown. On the right, specific deregulated adhesion molecules.

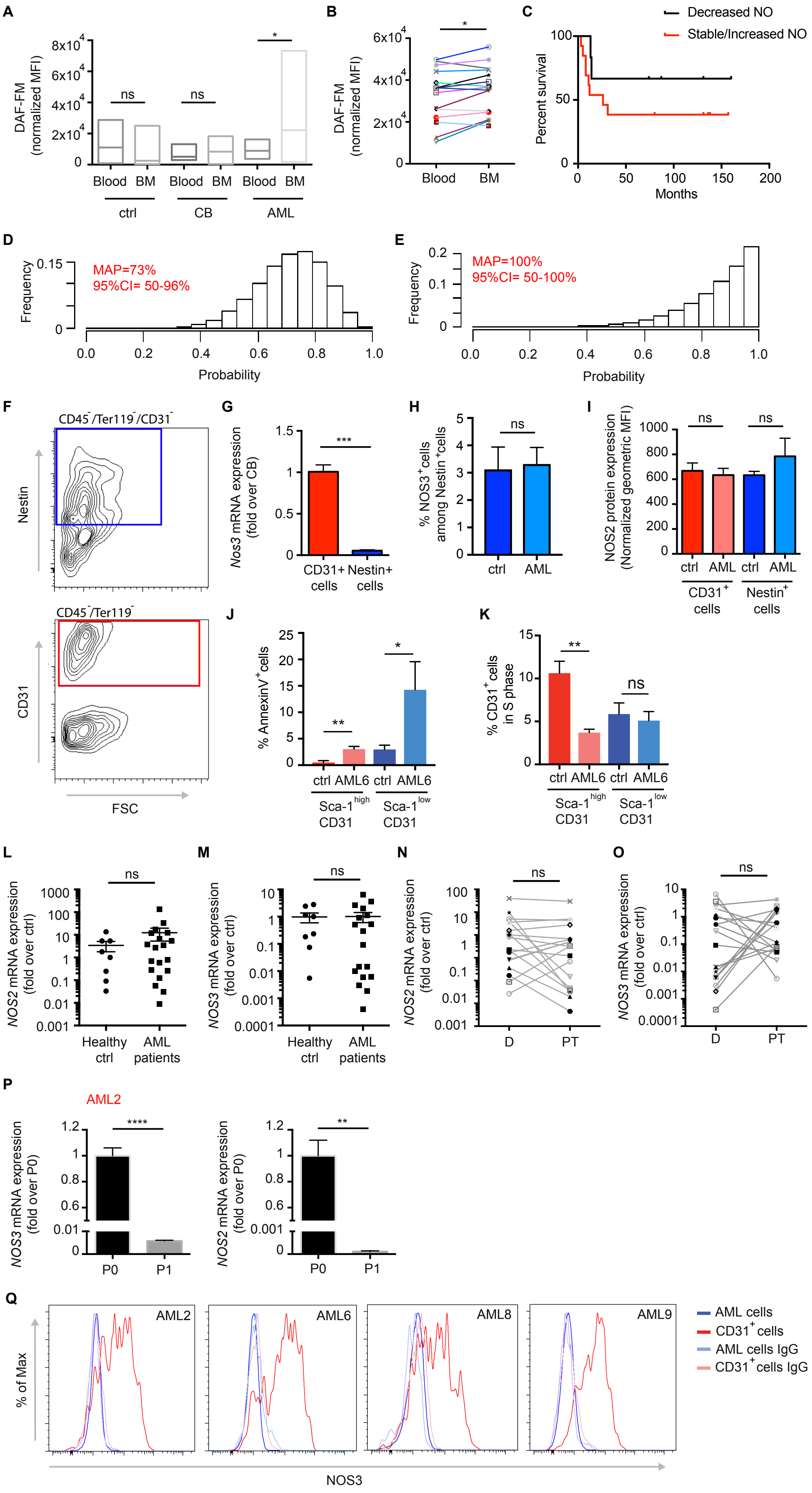
A
DAF-FM (normalized MFI)
ns
ns
*
Blood BM Blood BM Blood BM
ctrl
CB
AML
B
Blood BM
C
Decreased NO
Stable/Increased NO
Percent survival
Months
D
Frequency
MAP=73%
95%CI= 50-96%
Probability
E
MAP=100%
95%CI= 50-100%
F
CD45-/Ter119-/CD31-
Nestin
CD45-/Ter119-
CD31
FSC
G
Nos3 mRNA expression (fold over CB)
***
CD31+ cells
Nestin+ cells
H
% NOS3+cells among Nestin+cells
ctrl AML
I
NOS2 protein expression (Normalized geometric MFI)
CD31+ cells
Nestin+ cells
J
% AnnexinV+cells
ctrl AML6 ctrl AML6
Sca-1high CD31
Sca-1low CD31
K
% CD31+ cells in S phase
**
L
NOS2 mRNA expression (fold over ctrl)
Healthy ctrl
AML patients
M
NOS3 mRNA expression (fold over ctrl)
N
D PT
O
P
AML2
NOS3 mRNA expression (fold over P0)
****
P0 P1
NOS2 mRNA expression (fold over P0)
Q
AML2
AML6
AML8
AML9
% of Max
NOS3
AML cells
CD31+cells
AML cells IgG
CD31+cells IgG

Figure S5. Related to Figure 5. AML engraftment is associated to increased nitric oxide levels in the BM. (A) Nitric oxide levels in the BM and peripheral blood cells of non-transplanted mice (ctrl, n=13 and n=9 respectively), mice transplanted with CB derived $CD34^+$ cells (CB, n=24 and n=7 respectively) and AML patients' derived samples (AML2, 6, 9; n=10 and n=8 respectively), as depicted. Data are shown as min to max boxplots, the line inside the box representing the mean. **(B)** Nitric oxide levels in peripheral blood and BM cells of AML patients at diagnosis. Each point represents one patient; n=19. **(C)** Klapan-Meier survival curve of patients stratified based on the NO level normalization parameter. **(D)** Estimated distribution of probabilities of stable/increased NO levels in the BM in case of therapy failure, analyzed in our entire patient cohort (n=19) or **(E)** analyzed in intermediate risk group patients from our cohort (n=9). MAP= maximum a posteriori estimate. CI95= 95% credible interval. Bayesian inference. **(F)** Gating strategy in the $CD45^-Ter119^-$ BM stromal compartment for selecting $Nestin^+$ (top, blue box) and $CD31^+$ (down, red box) cells. **(G)** *Nos3* expression analyzed by qRT-PCR in $CD31^+$ and $Nestin^+$ cells BM cells, as depicted. Data are shown as mean ± s.e.m. **(H)** NOS3 protein expression in $Nestin^+$ mesenchymal cells in the BM of non-transplanted mice or AML xenografts, quantified by flow cytometry. Data are shown as mean ± s.e.m. **(I)** NOS2 protein expression analyzed by flow cytometry (Geometric MFI) in $CD31^+$ and $Nestin^+$ BM cells retrieved from non-transplanted mice or mice engrafted with AML patients-derived samples (AML6, 8 and 9). Data are shown as mean ± s.e.m. **(J)** % of apoptotic cells among different populations of BM-derived ECs, as depicted. Data are shown as mean ± s.e.m. **(K)** % cells in the S phase of cell cycle among different populations of BM-derived ECs, as depicted. Data are shown as mean ± s.e.m. **(L, M)** *NOS2* (L) and *NOS3* (M) expression analyzed by qRT-PCR in the BM of healthy controls (n=8) and in AML patients (n=19) from our cohort (Table S1). Data are shown as mean ± s.e.m. **(N, O)** *NOS2* (N) and *NOS3* (O) expression analyzed by qRT-PCR in the BM of AML patients (n=19) before and after therapy. **(P)** *NOS3* (left) and *NOS2* (right) expression analyzed by qRT-PCR in AML2 patient's derived cells (P0) and AML2 patient's derived xenografted cells (P1), as depicted. Data are shown as mean ± s.e.m. **(Q)** NOS3 protein expression measured via flow cytometry in $CD31^+$ and AML cells in the BM of AML xenografts, as depicted. Data are representative of triplicates. P values: ns: non-significant; $*p<0.05$; $** p<0.01$;$*** p<0.001$.

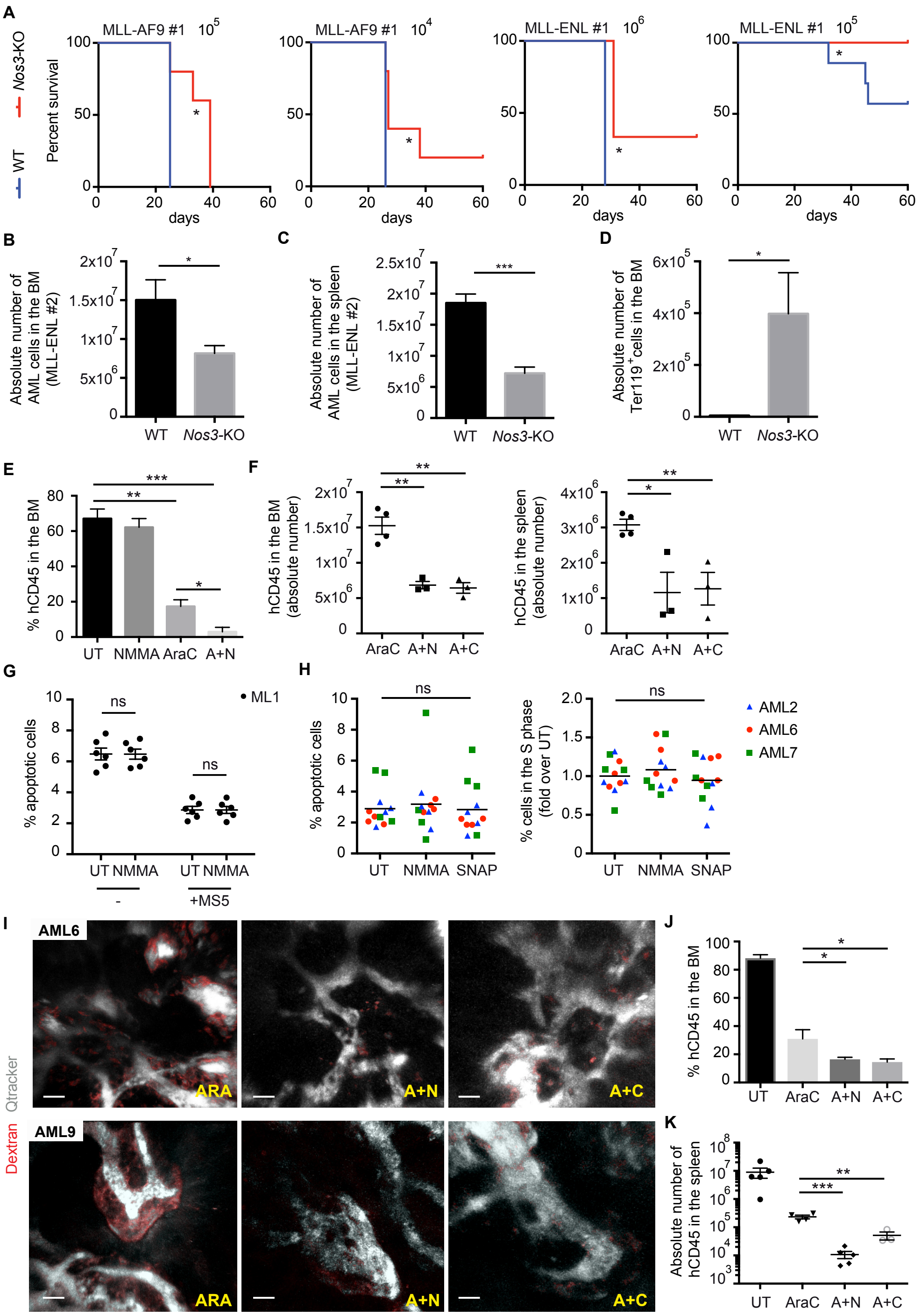

L

Patient sample	Treatment group	BM engraftment (% of hCD45 cells) ± s.e.m.	Spleen engraftment (% of hCD45 cells) ± s.e.m.
AML 6	UT	96.38 ± 0.423; n=3	71.53 ± 3.135; n=3
AML 6	NMMA	95.83 ± 0.088; n=3	56.2 ± 1.418; n=3
AML 6	CAV	95.08 ± 0.118; n=3	41.47 ± 0.393; n=3
AML 6	ARA	16.93 ± 1.01; n=3	10.56 ± 2.878; n=3
AML 6	A+N	7.043 ± 1.201; n=3	6.86 ± 3.164; n=3
AML 6	A+C	5.758 ± 0.126; n=3	3.183 ± 0.136; n=3
AML 9	UT	88.22 ± 2.463; n=5	70.56 ± 4.237; n=5
AML 9	NMMA	89.38 ± 2.453; n=5	77.48 ± 2.053; n=5
AML 9	CAV	89.36 ± 4.63; n=5	73.22 ± 4.475; n=5
AML 9	ARA	30.97 ± 6.527; n=3	48.87 ± 3.012; n=3
AML 9	A+N	16.48 ± 1.433; n=5	8.528 ± 2.541; n=5
AML 9	A+C	14.54 ± 2.158; n=4	25.85 ± 5.322; n=4

Figure S6. Related to Figure 6. Targeting vascular permeability cooperates with chemotherapy to improve AML treatment. (A) Survival curve of mice of depicted genotypes transplanted with murine MLL-AF9 #1 (top) or MLL-ENL #1 (bottom) AML cells at different doses. MLL-AF9 #1: 10^5 WT n=3; *Nos3*-KO n=5. 10^4 WT n=3; *Nos3*-KO n=5; MLL-ENL #1: 10^6 WT n=3; *Nos3*-KO n=3; 10^5 WT n=7; *Nos3*-KO n=4; 10^4 WT n=3; *Nos3*-KO n=3. **(B)** Absolute number of MLL-ENL #2 tomato$^+$ leukemic cells in the BM of mice of depicted genotypes. Data are shown as mean± s.e.m. **(C)** Absolute number of tomato$^+$ MLL-ENL leukemic cells in the spleen of mice of depicted genotypes. Data are shown as mean± s.e.m. **(D)** Absolute of Ter119$^+$ BMC in mice of depicted genotypes engrafted with tomato+ MLL-ENL leukemic cells. Data are shown as mean± s.e.m. **(E)** Human CD45$^+$ engraftment in the BM of mice engrafted with ML1 human AML cell line and treated with solvent (UT), NMMA alone, AraC alone, or combination of AraC with NMMA (A+N), as depicted; n=3. Data are shown as mean ± s.e.m. **(F)** Absolute number of human CD45$^+$ cells in the BM (left) and the spleen (right) of mice engrafted with ML.1 treated with AraC alone, or combination of AraC with NMMA (A+N) or Cavtatrin (A+C), as depicted; n=3 or more. Bars represent mean ± s.e.m. **(G)** % of apoptotic of ML1 cells grown alone or in co-culture with MS-5 and treated or not with NMMA. Bars represents mean ± s.e.m. Each dot represents a well. **(H)** % of apoptotic cells (left) and cells in the S phase (right) among AML primary patient-derived cells grown in co-culture with MS-5 and treated or not with NMMA and SNAP. Each dot represents one well. Bars represent mean. **(I)** Representative 3D reconstructions of the calvarium BM of mice transplanted with AML6 (top) and AML9 (bottom) patient-derived cells, treated with AraC alone or combination of AraC with NMMA or Cavtatrin, as depicted, and imaged via 2P microscopy using 655-conjugated Qtracker and TRITC-Dextran as vessel pooling agents. Bars respresent 30 μm. **(J)** Percentage of Human CD45$^+$ engraftment in the BM of mice engrafted with patient-derived sample AML9 and treated as in Figure 6D; n=5 per condition. Data are shown as mean ± s.e.m. **(K)** Absolute number of leukemic cells in the spleen of mice engrafted with AML9 patient-derived cells and treated or not with AraC, NMMA, Cavtatrin or combination of AraC and NMMA or Cavtatrin. Bars represent mean ± s.e.m. **(L)** Multi-organ analysis of PDX described in Figure 6 and S6. P values: ns: non-significant; *p<0.05; ** p<0.01; *** p<0.001; ****p<0.0001.

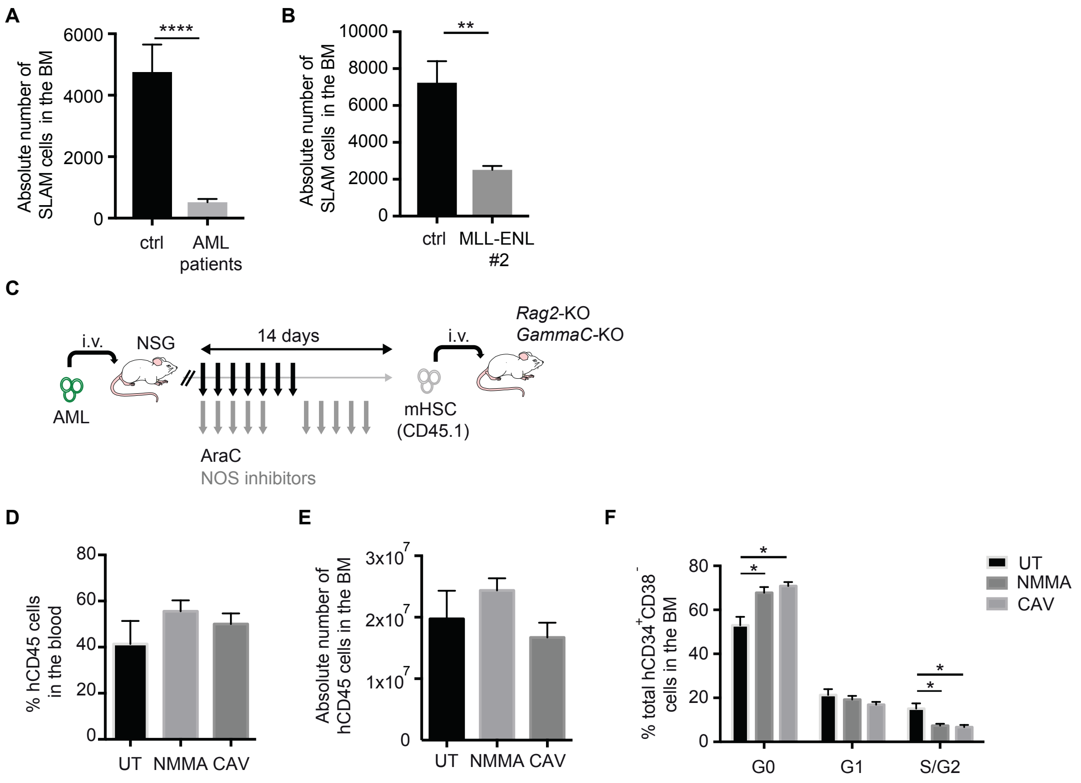

Figure S7. Related to Figure 7. Targeting vascular permeability potentiates HSPC function. (A) Absolute number of murine HSC in the BM of non-transplanted mice (n=5) or mice engrafted with AML patient-derived cells (AML6, n=4; AML9, n=5), as depicted. Data are shown as mean ± s.e.m. **(B)** Absolute number of murine HSC in the BM of non-transplanted mice (n=4) or mice engrafted with murine MLL-ENL leukemia (n=5), as depicted. Data are shown as mean ± s.e.m. **(C)** Schematic of the experiment. Residual murine CD45.1 cells in the BM of mice engrafted with AML6 patient-derived cells and treated with AraC or combination of AraC and NO inhibitors were transplanted i.v. in sub-lethally irradiated *Rag2*-KO/*GammaC*-KO CD45.2 recipient mice. Engraftment was assessed 8 weeks after transplantation (see Figure 7E-G). **(D)** Percentage of $hCD45^+$ cells in the peripheral blood of mice described in Figure 7H. Data are shown as mean ± s.e.m. **(E)** Absolute number of $hCD45^+$ cells in the BM of mice described in Figure 7H. Data are shown as mean ± s.e.m. **(F)** Percentage of HSPC in the different phase of cell cycle in the BM of mice in Figure 7H. Data are shown as mean ± s.e.m. p values: * $p<0.05$; **$p<0.01$; **** $p<0.0001$.
